# Repression of Virulence in *Staphylococcus aureus* by 2‐Aminothiol‐Mediated Quorum Quenching

**DOI:** 10.1002/anie.9489770

**Published:** 2026-07-23

**Authors:** Bengt H. Gless, Riccardo Milazzo, Martin S. Bojer, Benjamin S. Sereika‐Bejder, Hanne Ingmer, Christian A. Olsen

**Affiliations:** ^1^ Center For Biopharmaceuticals and Department of Drug Design and Pharmacology Faculty of Health and Medical Sciences University of Copenhagen Copenhagen Denmark; ^2^ Department of Veterinary and Animal Sciences Faculty of Health and Medical Sciences University of Copenhagen Frederiksberg Denmark

**Keywords:** autoinducing peptides, cysteamine, quorum sensing, *S. aureus*, skin infection

## Abstract

We present a novel strategy for the inhibition of quorum sensing in *Staphylococcus* bacteria. Previous work has focused on the development of peptides that can compete with native autoinducing peptides (AIPs), to antagonize the receptor histidine kinase responsible for activation of quorum sensing and thereby repress the expression of virulence genes. Here we report simple analogues of cysteine that can eliminate the native AIPs extracellularly, before they activate their receptor, and validate that this alternative strategy can also be applied to turn off quorum sensing in different *Staphylococcus aureus* reporter strain assays. Further, we show that two selected compounds can reduce skin infection caused by MRSA in a murine model. This work provides impetus for the development of simple small molecules for the topical treatment of staphylococcal skin infections. We anticipate that further studies, including delivery strategies, will help illuminate this potential.

## Graphical abstract

Simple analogues of cysteine that can eliminate the native autoinducing peptides (AIPs) of staphylococci extracellularly, before they activate their receptor, and turn off quorum sensing in different *Staphylococcus aureus*. The elimination of AIPs occurs through a mechanism similar to native chemical ligation (NCL). Further, this strategy showed promise for reducing skin infection caused by MRSA in a murine model.

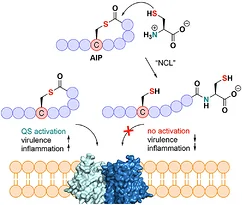

The Gram‐positive bacterium *Staphylococcus aureus* is an opportunistic human pathogen, causing skin and soft tissue infections, among others, and alarming rates of emerging methicillin‐resistant *S. aureus* (MRSA) strains are challenging the global health [1, 2, 3]. Thus, novel treatment options are needed. An alternative to conventional antibiotics that has received significant attention, is reducing virulence rather than eliminating the bacteria [4, 5, 6]. Expression of bacterial virulence factors is often regulated through quorum sensing (QS), which is the ability of the bacteria to change gene expression in response to cell density [7, 8]. In staphylococci, the QS machinery is encoded by the accessory gene regulator (*agr*) locus and relies on the secretion and detection of cyclic thiodepsipeptides, called autoinducing peptides (AIPs) that auto‐activate their AgrC receptor histidine kinase (Figure 1), which phosphorylates the response regulator (AgrA), in turn, leading to global changes in gene expression downstream, including expression of virulence factors [9]. However, crosstalk has also been observed, where AIPs of one species are inhibitory towards *agr* systems of other species and even between strains of the four different *S. aureus agr* types *(ag*r‐I–IV) [5, 10]. Such QS inhibition can repress virulence gene expression in *S. aureus*, which has been demonstrated to attenuate MRSA infection in murine skin models [11, 12, 13]. A recent pioneering study even investigated the disease outcome of *S. aureus* infection upon co‐colonization with the AIP producing commensal bacterium *Staphylococcus hominis* in a human clinical trial [14].

**FIGURE 1:**
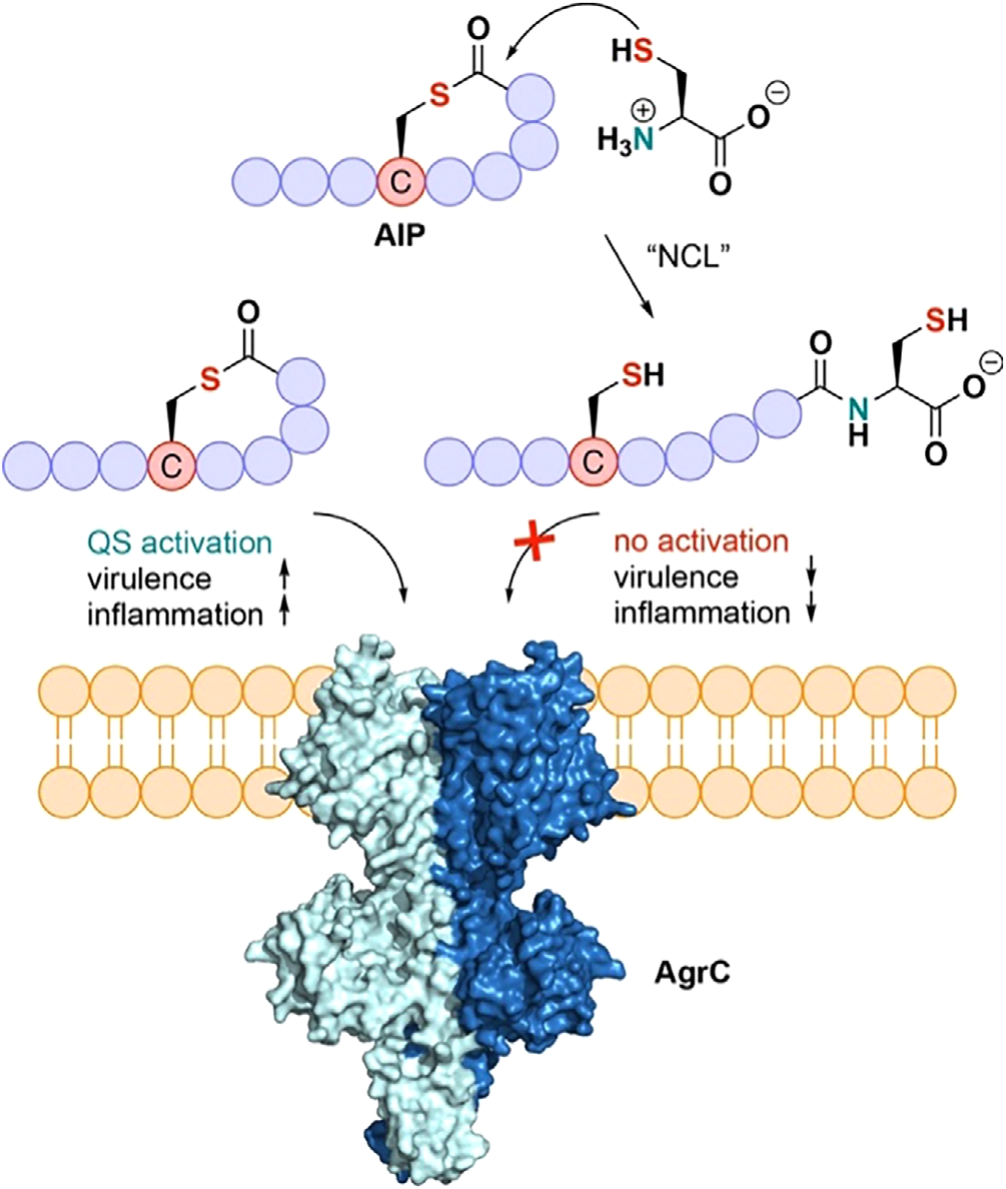
Cartoon representation of the quorum quenching strategy, relying on AIP destruction by native chemical ligation (NCL)‐like reaction.

Our laboratory has developed a platform for mapping the chemical messenger molecules (AIPs) responsible for QS, which is based on the covalent trapping of AIPs in a highly chemo‐selective manner, using solid supported cysteine [15]. In extension of this work, we hypothesized that cysteine in solution could likewise react with excreted AIPs through a native chemical ligation [16] type reaction to linearize the AIP and, in turn, inhibit QS (Figure 1). To test this hypothesis, we first incubated *S. aureus* AIP‐I with cysteine (**Cys**) at different concentrations and evaluated the destruction of the thiolactone ring over time in an HPLC‐based assay (Figure S1A,B). At 40 equivalents of added cysteine, the AIP was rapidly destroyed with close‐to‐full conversion at the 30‐min time point, and we therefore tested a series of additional aminothiol compounds from commercial sources.

The most efficacious compounds were cysteine (**Cys**) and cysteamine (**CA**), while homocysteine (**hCys**) was less effective and no elimination was observed for penicillamine (**PA**) and *N*‐acetylcysteine (**NAC**) after 30 min incubation (Figure 2A,B). In addition, we tested the destruction of AIP‐I in a differently designed UPLC assay, where the products were quenched with acid, resulting in appearance of potential transient thioester species (Figure S2). This assay revealed the presence of a minor ring‐opened species resulting from the NAC treatment, which agrees with the presence of an equilibrium between the AIP and its C‐terminal thioester species as suggested in Figure S2H. Because both species would give the same quenching product in the former UPLC assay format, these data are also in agreement with complete lack of conversion shown in said assay (Figure 2B). We further tested the potential effect of aminolysis by treatment with ethylenediamine or 1‐aminobutane. Not surprisingly, based on recent investigation of the reactivity of thioesters toward amine nucleophiles at neutral pH [17], only minor degrees of aminolysis were observed (Figure S2). Finally, we tested whether the AIP destruction would also occur in the more complex environment of a growth medium. We therefore spiked fresh medium with AIP and treated the solution with either **Cys** or **NAC** for 30 min, before applying our previously developed AIP trapping workflow (Figure S3A). The mass spectrometry, following enrichment of residual AIP showed the presence of AIP after treatment with **NAC** but not after treatment with cysteine, supporting that occurrence of AIP destruction by the 1,2‐aminothiol in the growth medium and showing that high concentrations of thiol‐containing species (i.e., 10 mM **NAC** in addition to cysteine‐containing peptides present in the growth medium) are not sufficient for destroying the AIP (Figure S3).

**FIGURE 2:**
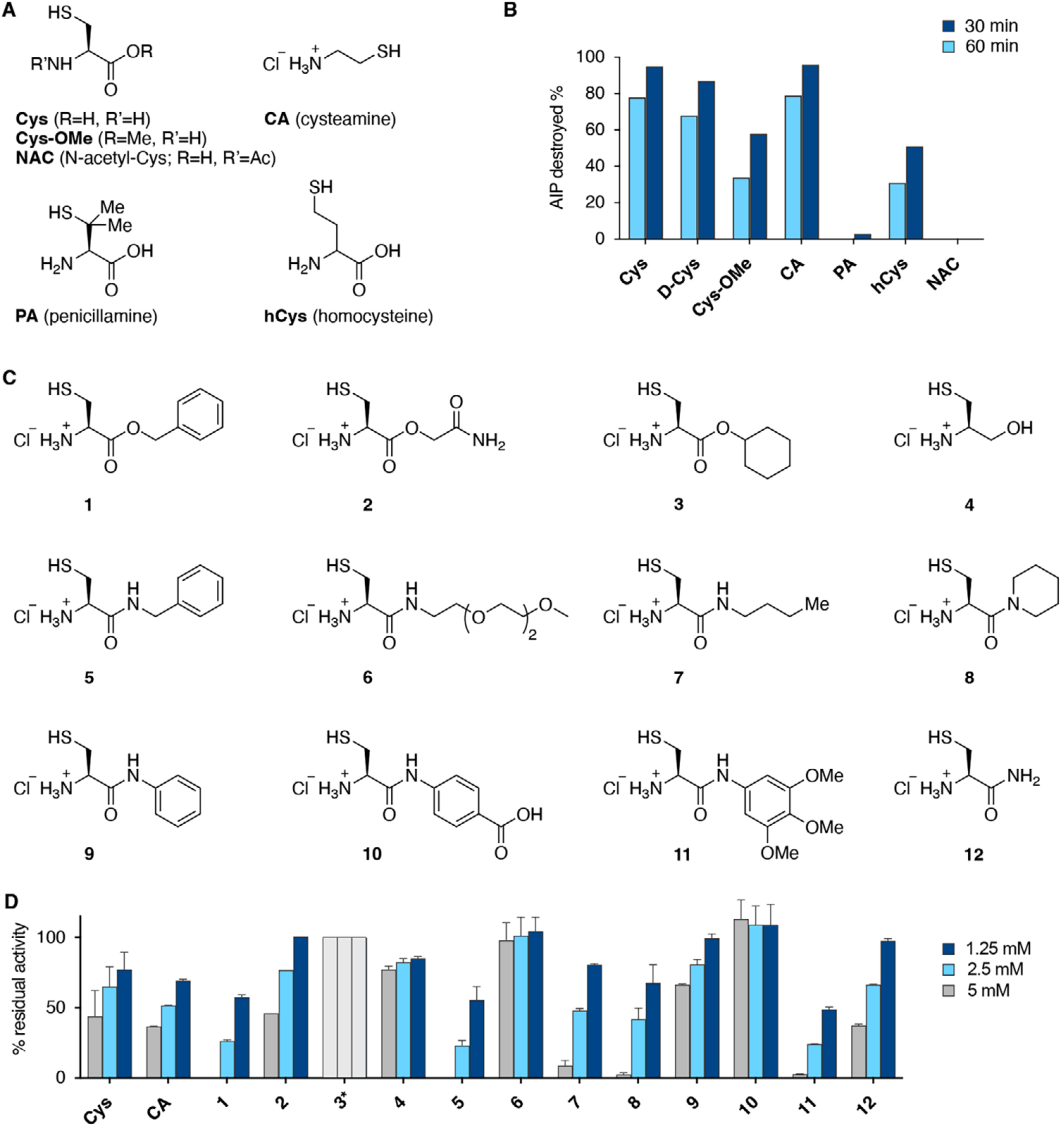
Compound collection. (A) Commercially obtained 2‐aminothiols. (B) Bar graph representation of the ability of the commercial compound collection to destroy the thiolactone moiety of *S. aurues* AIP‐I as measured in the UPLC assay described in Figure S1. (C) Synthesized compounds (see, Schemes S1–S4). (D) Inhibition of *S. aureus agr‐*I in fluorescent reporter assay. Note (*): Compound **3** visibly precipitated upon dilution in medium.

We then tested the effect of **Cys** and **NAC** in a reporter strain where the *agr*‐regulated P3 promoter of the central virulence regulator, RNAIII is fused to *yfp*, encoding the yellow fluorescent protein (YFP) [18]. These assays showed that the free 2‐aminothiol (**Cys**) indeed was able to inhibit QS as expected, because they can engage in the NCL‐like mechanism, while **NAC** exhibited no effect in agreement with its inability to cause the S‐to‐N acyl shift in the NCL reaction [16] (Figure S4). To address whether the rather high concentrations of compound added had any effect on the bacteria's fitness, which could indirectly affect QS, we performed independent CFU count experiments. No difference in CFU count were observed for **Cys** (5 mM) or **NAC** (5 mM), compared to vehicle control cultures after 24 h (Figure S5).

Next, we extended our series of 2‐aminothiol‐containing small molecules through chemical synthesis (compounds **1**–**12**) and screened all compounds for quorum quenching activity against the *S. aureus agr*‐I specificity group in the fluorescent reporter strain assay. The compound collection was designed to incorporate both esters (**1**–**3**), an amino alcohol (**4**), and amides (**5**–**12**) with various electronic properties and polarity to cover a span of p*K*a values of the thiol and amino groups as well as varying polarity (Figure 2C and Schemes S1–S4).

The compound screening against the *S. aureus agr*‐I YFP reporter strain revealed that most compounds could quench QS at 5 mM concentration; however, few exhibited >50% inhibition at the 2.5 mM concentration (**1**, **5**, **7**, **8**, and **11**), and only **1**, **5**, and **11** exhibited >40% inhibition at 1.25 mM (Figure 2D). We envisioned that the amide‐containing compounds would be less prone to hydrolysis than the ester‐containing (**1**) and therefore selected compounds **5** and **11** for further evaluation in dose–response experiments with all four *S. aureus agr* group I–IV reporter strains. We also included the parent compound cysteine (**Cys**) and the minimal analogue cysteamine (**CA**) for comparison. Across all four specificity groups, cysteine (**Cys**) was least potent and compounds **5** and **11** were equipotent, showing the highest activity (Figure 3). Interestingly, we observed the lowest degree of repression of *agr*‐IV, which is in line with the hyperactivity of this strain and the lowest inhibitory potential seen in our previous studies of inhibition with AIPs.

**FIGURE 3:**
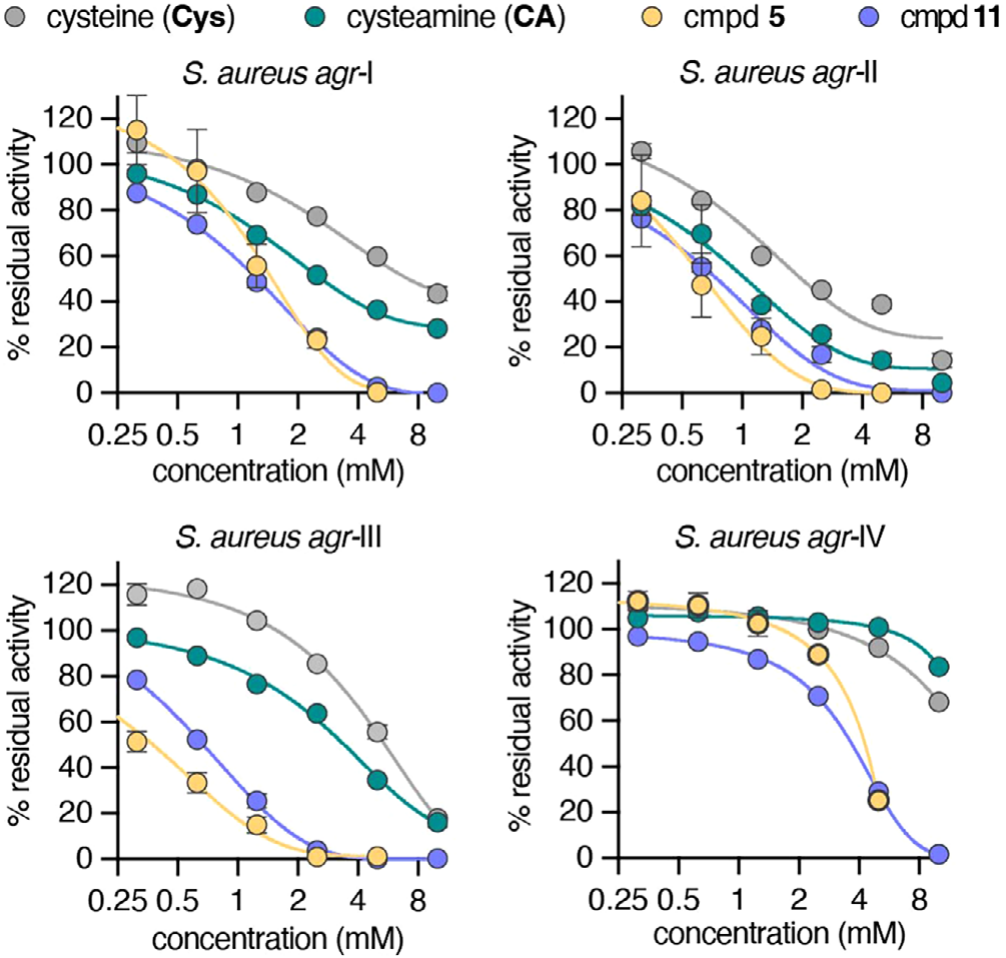
Dose‐response curves for quorum sensing inhibition in *S. aureus agr‐*I–IV P3 reporter strain assays. At least two individual assays were performed in technical triplicate. Each curve represents the two biological replicates with IC_50_ curves fitted by nonlinear regression (variable slope) in GraphPad Prism; data points and error bars represent the mean ± SD.

With the promising data relying on activation/inhibition of RNAIII expression in these reporter strains, we were interested in gaining further proof that the observed effect on QS was indeed due to destruction of native AIP. We therefore designed an experiment where we pre‐incubated activating AIP‐I with serial dilutions of either **NAC** or **Cys** for 30 min before performing *β*‐lactamase reporter assays, which showed no inhibition of QS for **NAC** and a dose‐dependent reduction in QS for **Cys** (Figure S6A,B). Further, we tested synthetic linearized AIP that contains an additional cysteine residue at the C‐terminus (i.e., the product of **AIP‐I**–**Cys** NCL reaction), which produced a minor level of activation (<10%) at µM concentrations, but no activation at the common activator concentration of 100 nM (Figure S6C). These assays provide further support of a mechanism of action involving NCL‐like destruction of the activating AIP to inhibit QS.

We also developed a reporter strain where the green fluorescent protein (GFP) gene is fused to the *spa* promoter, which is repressed during activated QS [10]. Thus, an **AIP‐I** activated culture is nonfluorescent, and fluorescence increases when QS is inhibited. In this newly developed *spa::gfp* plasmid‐based reporter assay [10], the potent inhibitor of *agr*‐I (**AIP‐III_D4A_**) [19] was able to inhibit an activated culture, while **NAC** showed no activity against the **AIP‐I** activated culture (Figure 4A), which is in agreement with its inability to engage in the NCL‐like AIP destruction as shown in our HPLC assays. We then selected cysteamine (**CA**) and compound **11** due to its high potency and higher aqueous solubility compared to compound **5**, for further testing in this assay. Both 2‐aminothiol compounds exhibited inhibition of QS in the *spa‐gfp* reporter assay, with the effect of **CA** at 20 mM concentration reaching the quenching level of the potent inhibitor **AIP‐III_D4A_** (Figure 4A). Thus, these data provide additional proof, supporting our hypothesis that the excreted native AIP is indeed eliminated by our 2‐aminothiol compounds to furnish the quorum quenching effect. Further, we performed dose–response experiments with **CA** and compound **11**, but experienced challenges with the solubility of compound **11** at concentrations above 5 mM in this assay, which prohibited determination of IC_50_ values for this otherwise potent compound (Figure 4B). The **CA** exhibited similar IC_50_ values regardless of whether the culture had been activated by **AIP‐I** at 50 nM (IC_50_ 6.9 ± 0.57 mM) or 100 nM (IC_50_ 7.0 ± 0.44 mM) concentration. This is different from the behavior of inhibitory AIPs in this assay but may reflect that these quenchers are not competing for binding to the receptor.

**FIGURE 4:**
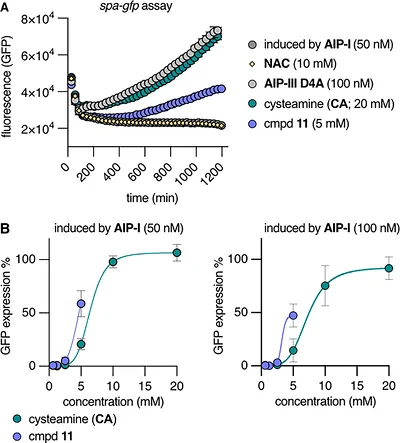
Evaluation of the ability of selected 2‐aminothiols to quench already turned on quorum sensing in a *spa‐gfp* reporter assay [10]. (A) Continuous assay with all cultures induced by **AIP‐I** (50 nM) and challenged with the selected compounds as noted. (B) Dose–response experiments for **CA** and compound **11** against cultures activated by **AIP‐I** at either 50 nM or 100 nM concentration. Each curve represents the two biological replicates with IC_50_ curves fitted by nonlinear regression (variable slope) in GraphPad Prism; data points and error bars represent the mean ± SD.

Finally, we tested the ability of compound **11** to treat a skin infection caused by MRSA in a previously reported murine skin infection model [10, 20]. We also included cysteamine (**CA**), because it is an approved drug substance for human clinical use in the treatment of cystinosis [21], with a well‐documented toxicity profile [22]. Gratifyingly, a significant reduction in the skin lesion size, measured on day 2, was observed after a single treatment with **CA** or **11** (10 mM), similar to that achieved by daily application of Fucidin (39 mM). On day 4, however, the effect was not statistically significant for any of the treatments, due to the reduced skin lesion area also observed for the vehicle control mice (Figure 5).

**FIGURE 5:**
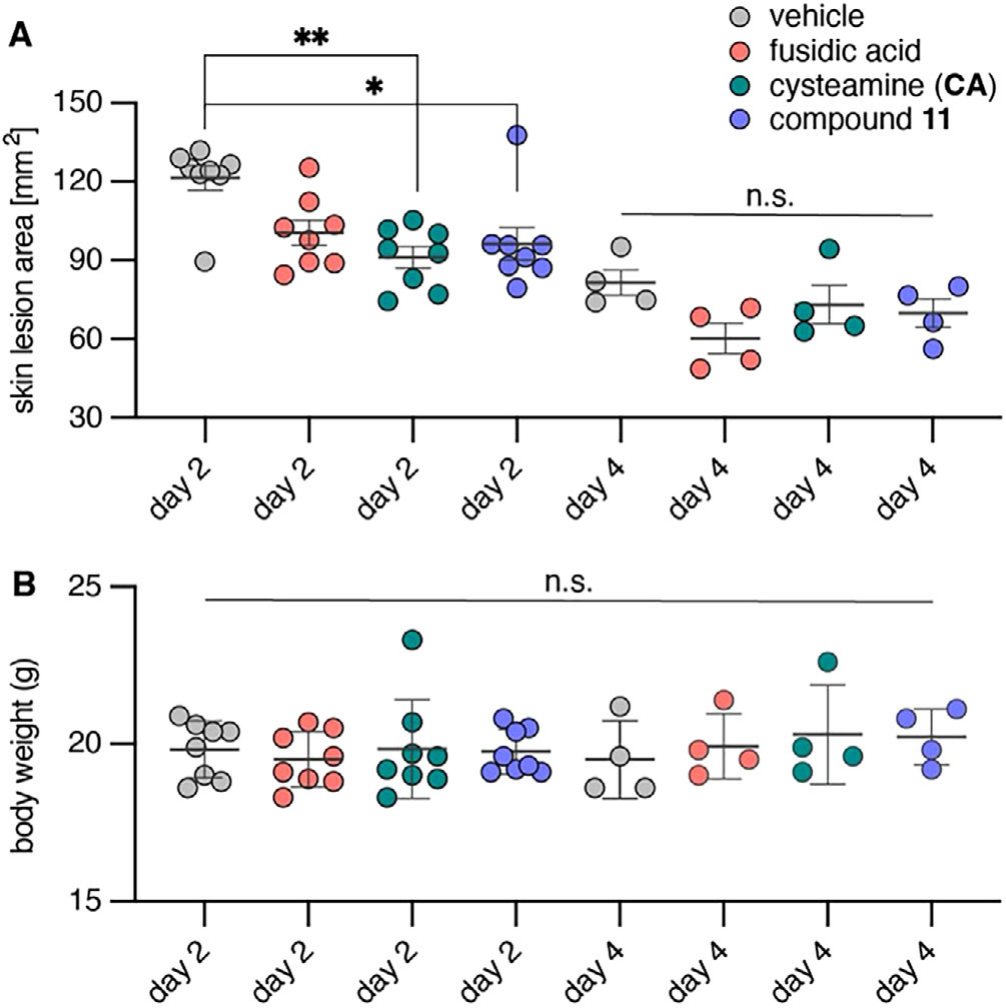
Murine skin infection model performed with vehicle control and fusidic acid groups. All groups were of eight animals and half were sacrificed on day two, ending up with four animals per group on day four. (A) Skin lesion size measured at days 2 and 4. (B) Body weight measurements. Data are presented as mean and error bars represent the standard deviation (SD) of the mean. Statistical significance between treatment and vehicle groups was assessed by one‐way analysis of variance (ANOVA) with multiple comparisons (Dunnett's test), *p* > 0.05 (ns), *p* < 0.05 (*), *p* < 0.01 (**). The murine model was performed under contract at Statens Serum Institut (DK) as previously described [20]. All animal experiments were approved by the National Committee of Animal Ethics, Denmark.

In conclusion, we provide evidence to support that elimination of AIPs secreted by *S. aureus* (and MRSA), using simple small molecules with a 2‐aminothiol motif, should be considered as a novel anti‐virulence strategy. We show that the thiolactone moiety of AIPs is destroyed by a native chemical ligation mechanism. Further, we show that this approach can be applied for QS inhibition in bacterial culture, using two fundamentally different reporter strain assays. Finally, promising data were obtained in a preliminary in vivo study, in a murine MRSA skin infection model.

It is our hope that these data will inspire further investigations of the in vivo potential of quorum quenching by AIP destruction, including additional dosing and delivery experiments, which may provide new avenues for the treatment of bacterial infections.

## Author Contributions

**Bengt H. Gless**: conceptualization, investigation, writing – review and editing, formal analysis, methodology, visualization, data curation. **Riccardo Milazzo**: investigation, writing – review and editing, formal analysis. **Martin S. Bojer**: investigation, writing – review and editing, methodology, formal analysis. **Benjamin S. Sereika‐bejder**: investigation, writing – review and editing. **Hanne Ingmer**: funding acquisition, writing – review and editing, resources, supervision. **Christian A. Olsen**: funding acquisition, writing – original draft, visualization, writing – review and editing, project administration, supervision, resources.

## Conflicts of Interest

The authors declare no conflicts of interest.

## Supporting information




**Supporting File**: The authors have cited additional references within the Supporting Information [23, 24, 25].

## Data Availability

The data that support the findings of this study are available from the corresponding author upon reasonable request.

## References

[1] M. Z. David and R. S. Daum , “Community‐Associated Methicillin‐Resistant *Staphylococcus aureus*: Epidemiology and Clinical Consequences of an Emerging Epidemic,” Clinical Microbiology Reviews 23 (2010): 616–687, 10.1128/CMR.00081-09. 20610826 PMC2901661

[2] F. R. DeLeo , M. Otto , B. N. Kreiswirth , and H. F. Chambers , “Community‐associated Meticillin‐resistant *Staphylococcus aureus* ,” Lancet 375 (2010): 1557–1568, 10.1016/S0140-6736(09)61999-1. 20206987 PMC3511788

[3] A. S. Lee , H. de Lencastre , J. Garau , et al., “Methicillin‐resistant *Staphylococcus aureus* ,” Nature reviews Disease Primers 4 (2018): 18033, 10.1038/nrdp.2018.33. 29849094

[4] G. Ji , R. C. Beavis , and R. P. Novick , “Cell Density Control of Staphylococcal Virulence Mediated by an Octapeptide Pheromone,” Proceedings of the National Academy of Sciences of the United States of America 92 (1995): 12055–12059, 10.1073/pnas.92.26.12055. 8618843 PMC40295

[5] G. Ji , R. Beavis , and R. P. Novick , “Bacterial Interference Caused by Autoinducing Peptide Variants,” Science 276 (1997): 2027–2030, 10.1126/science.276.5321.2027. 9197262

[6] S. W. Dickey , G. Y. C. Cheung , and M. Otto , “Different Drugs for Bad Bugs: Antivirulence Strategies in the Age of Antibiotic Resistance,” Nature Reviews Drug Discovery 16 (2017): 457–471, 10.1038/nrd.2017.23. 28337021 PMC11849574

[7] C. M. Waters and B. L. Bassler , “QUORUM SENSING: Cell‐to‐Cell Communication in Bacteria,” Annual Review of Cell and Developmental Biology 21 (2005): 319–346, 10.1146/annurev.cellbio.21.012704.131001. 16212498

[8] S. Mukherjee and B. L. Bassler , “Bacterial Quorum Sensing in Complex and Dynamically Changing Environments,” Nature Reviews Microbiology 17 (2019): 371–382, 10.1038/s41579-019-0186-5. 30944413 PMC6615036

[9] B. Wang and T. W. Muir , “Regulation of Virulence in *Staphylococcus aureus*: Molecular Mechanisms and Remaining Puzzles,” Cell Chemical Biology 23 (2016): 214–224, 10.1016/j.chembiol.2016.01.004. 26971873 PMC4847544

[10] B. H. Gless , B. S. Sereika‐Bejder , I. Jensen , et al., “Mapping of Quorum Sensing Interaction Network of Commensal and Pathogenic staphylococci,” mBio 16 (2025): e0096725, 10.1128/mbio.00967-25. 40667988 PMC12345137

[11] T. Nakatsuji , T. H. Chen , S. Narala , et al., “Antimicrobials From human Skin Commensal Bacteria Protect Against *Staphylococcus aureus* and Are Deficient in Atopic Dermatitis,” Science Translational Medicine 9 (2017): eaah4680, 10.1126/scitranslmed.aah4680. 28228596 PMC5600545

[12] A. E. Paharik , C. P. Parlet , N. Chung , et al., “Coagulase‐Negative Staphylococcal Strain Prevents *Staphylococcus aureus* Colonization and Skin Infection by Blocking Quorum Sensing,” Cell Host & Microbe 22 (2017): 746–756.e5, 10.1016/j.chom.2017.11.001. 29199097 PMC5897044

[13] M. M. Brown , J. M. Kwiecinski , L. M. Cruz , et al., “Novel Peptide From Commensal Staphylococcus Simulans Blocks Methicillin‐Resistant *Staphylococcus aureus* Quorum Sensing and Protects Host Skin From Damage,” Antimicrobial Agents and Chemotherapy 64 (2020): e00172–00120, 10.1128/AAC.00172-20. PMC7269516 32253213

[14] T. Nakatsuji , T. R. Hata , Y. Tong , et al., “Development of a Human Skin Commensal Microbe for Bacteriotherapy of Atopic Dermatitis and Use in a Phase 1 Randomized Clinical Trial,” Nature Medicine 27 (2021): 700–709, 10.1038/s41591-021-01256-2. PMC8052297 33619370

[15] B. H. Gless , M. S. Bojer , P. Peng , M. Baldry , H. Ingmer , and C. A. Olsen , “Identification of Autoinducing Thiodepsipeptides From staphylococci Enabled by Native Chemical Ligation,” Nature Chemistry 11 (2019): 463–469, 10.1038/s41557-019-0256-3. 31011175

[16] P. E. Dawson , T. W. Muir , I. Clark‐Lewis , and S. B. Kent , “Synthesis of Proteins by Native Chemical Ligation,” Science 266 (1994): 776–779, 10.1126/science.7973629. 7973629

[17] B. H. Gless , S. H. Schmied , B. S. Bejder , and C. A. Olsen , “Förster Resonance Energy Transfer Assay for Investigating the Reactivity of Thioesters in Biochemistry and Native Chemical Ligation,” JACS Au 3 (2023): 1443–1451, 10.1021/jacsau.3c00095. 37234128 PMC10207088

[18] C. L. Malone , B. R. Boles , K. J. Lauderdale , M. Thoendel , J. S. Kavanaugh , and A. R. Horswill , “Fluorescent Reporters for *Staphylococcus aureus* ,” Journal of Microbiological Methods 77 (2009): 251–260, 10.1016/j.mimet.2009.02.011. 19264102 PMC2693297

[19] Y. Tal‐Gan , D. M. Stacy , M. K. Foegen , D. W. Koenig , and H. E. Blackwell , “Highly Potent Inhibitors of Quorum Sensing in *Staphylococcus aureus* Revealed Through a Systematic Synthetic Study of the Group‐III Autoinducing Peptide,” Journal of the American Chemical Society 135 (2013): 7869–7882, 10.1021/ja3112115. 23647400

[20] C. Vingsbo Lundberg and N. Frimodt‐Møller , “Efficacy of Topical and Systemic Antibiotic Treatment of Meticillin‐resistant *Staphylococcus aureus* in a Murine Superficial Skin Wound Infection Model,” International Journal of Antimicrobial Agents 42 (2013): 272–275, 10.1016/j.ijantimicag.2013.05.008. 23837927

[21] A. Carneiro and D. H. Jones , “Advances in Pharmacological Treatments for Cystinosis: Cysteamine and Its Alternatives,” ACS Pharmacology and Translational Science 9 (2026): 272–281, 10.1021/acsptsci.5c00633. 41710743 PMC12910489

[22] M. T. Besouw , R. Bowker , J. P. Dutertre , et al., “Cysteamine Toxicity in Patients With Cystinosis,” Journal of Pediatrics 159 (2011): 1004–1011, 10.1016/j.jpeds.2011.05.057. 21784456

[23] L. I. Kupferwasser , M. R. Yeaman , C. C. Nast , et al., “Salicylic Acid Attenuates Virulence in Endovascular Infections by Targeting Global Regulatory Pathways in *Staphylococcus aureus* ,” Journal of Clinical Investigation 112 (2003): 222–233, 10.1172/JCI200316876. 12865410 PMC164286

[24] J. Canovas , M. Baldry , M. S. Bojer , et al., “Cross‐Talk Between *Staphylococcus aureus* and Other Staphylococcal Species via the Agr Quorum Sensing System,” Frontiers in Microbiology 7 (2016): 1733, 10.3389/fmicb.2016.01733. 27877157 PMC5099252

[25] J. Canovas , M. Baldry , M. S. Bojer , et al., “Corrigendum: Cross‐Talk Between *Staphylococcus aureus* and Other Staphylococcal Species via the Agr Quorum Sensing System,” Frontiers in Microbiology 8 (2017): 1949, 10.3389/fmicb.2017.01949. 29021792 PMC5634338

