# Supplementary material for: Repression of Virulence in Staphylococcus aureus by 2‐Aminothiol‐Mediated Quorum Quenching
Source: Angew Chem Int Ed Engl. 2026 Jul 23;65(37):e9489770. doi: 10.1002/anie.9489770 (PMC13548902; doi:10.1002/anie.9489770)

# Supporting Information

## Repression of Virulence in *S. aureus* by 2-Aminothiol-mediated Quorum Quenching

Bengt H. Gless,[a] Riccardo Milazzo,[a] Martin S. Bojer,[b] Benjamin S. Sereika-Bejder,[a] Hanne Ingmer,[b] and Christian A. Olsen*[a]

[a] Dr. B. H. Gless, R. Milazzo, Dr. B. S. Sereika-Bejder, Prof. Dr. C. A. Olsen
Center for Biopharmaceuticals and Department of Drug Design and Pharmacology
Faculty of Health and Medical Sciences, University of Copenhagen
Jagtvej 160, DK-2100, Copenhagen, Denmark.
E-mail: cao@sund.ku.dk

[b] Dr. M. S. Bojer, Prof. Dr. H. Ingmer
Department of Veterinary and Animal Sciences
Faculty of Health and Medical Sciences, University of Copenhagen
DK-1870 Frederiksberg C, Denmark

**Table of Contents**


Supporting Schemes ..... 3

Supporting Figures ..... 5

Materials and Methods ..... 9

- Reagents and materials ..... 9
- Analysis and purification ..... 9
- HPLC assays ..... 9
- Bacterial assays and murine skin model ..... 11
- Synthesis of protected 2-amino-thiol precursors ..... 13
- Synthesis of hydrochloride salts of 2-aminothiol compounds ..... 17
- Synthesis of peptide S13 ..... 20

Supporting references ..... 22

Copies of NMR spectra of final compounds ..... 23

Copies of HPLC chromatograms ..... 35

# Supporting Schemes

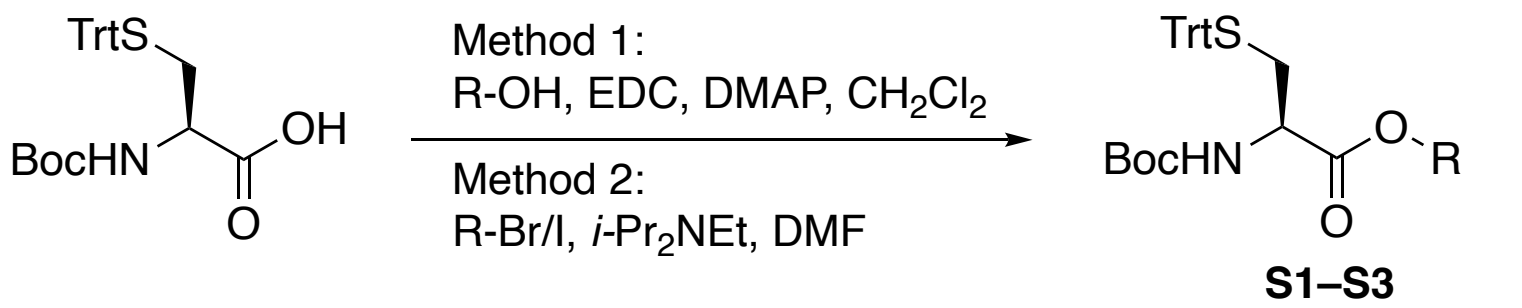


Supporting Scheme S1. Synthesis of protected cysteine ester analogues (S1–S3).[1]

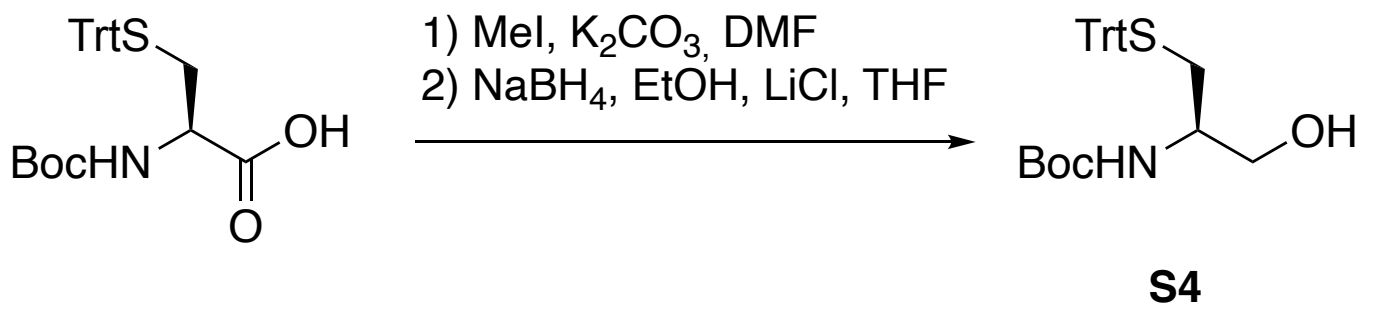


**Supporting Scheme S1.** Synthesis of protected 2-aminothiol (**S4**) from protected cysteine.[2]

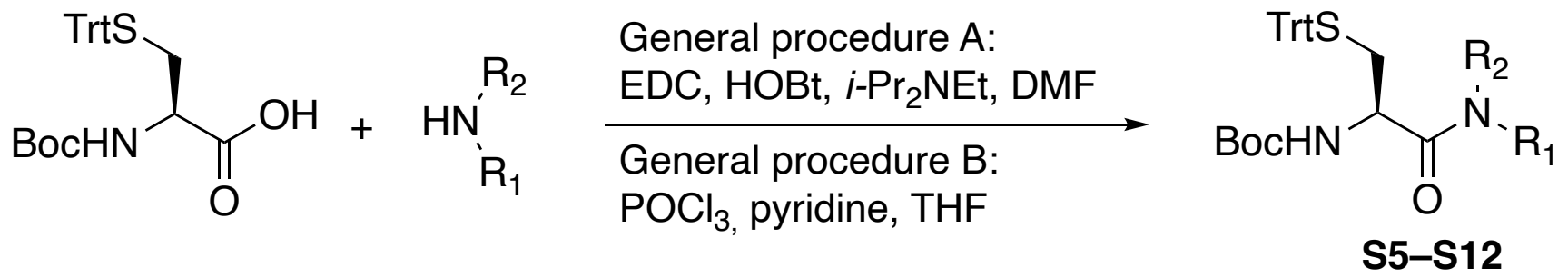


**Supporting Scheme S3.** Synthesis of protected cysteine amide analogues (**S5**–**S12**).[3]

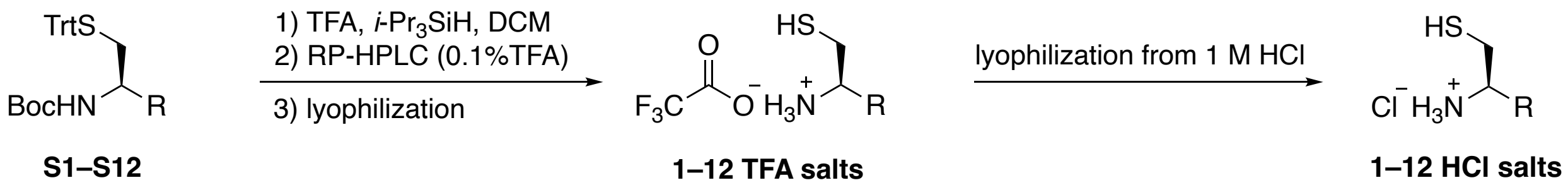


**Supporting Scheme S4**. Synthesis of hydrochloride salts (**1**–**12**) from protected 2-aminothiol precursors.[4]

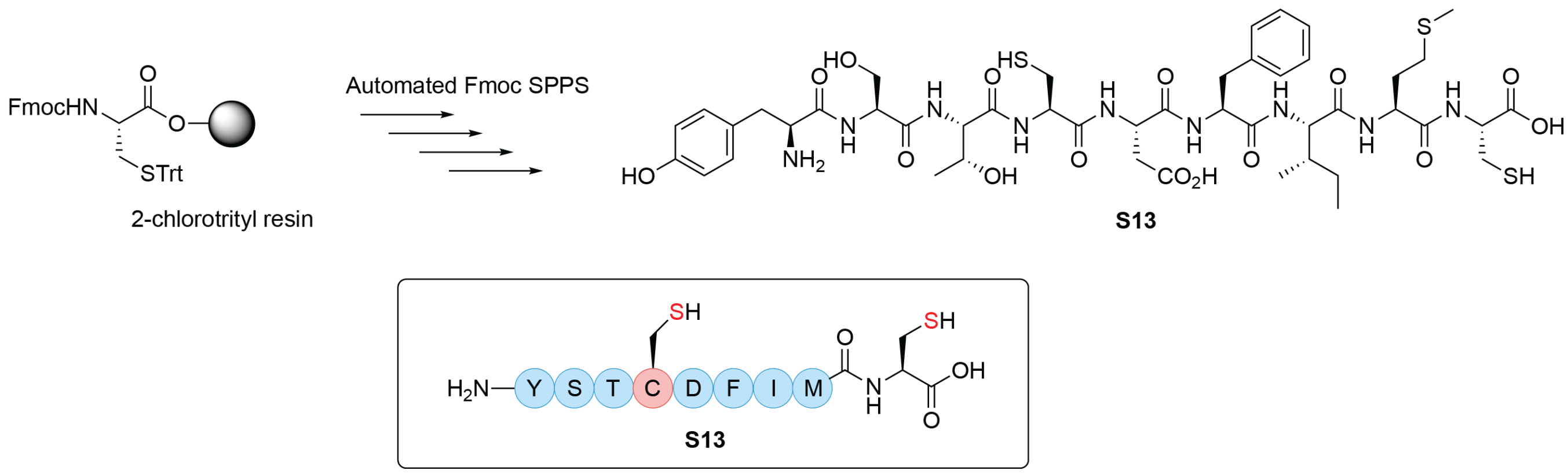


**Supporting Scheme S5**. Synthesis of peptide **S13** (for details and conditions, see the experimental section).

# Supporting Figures

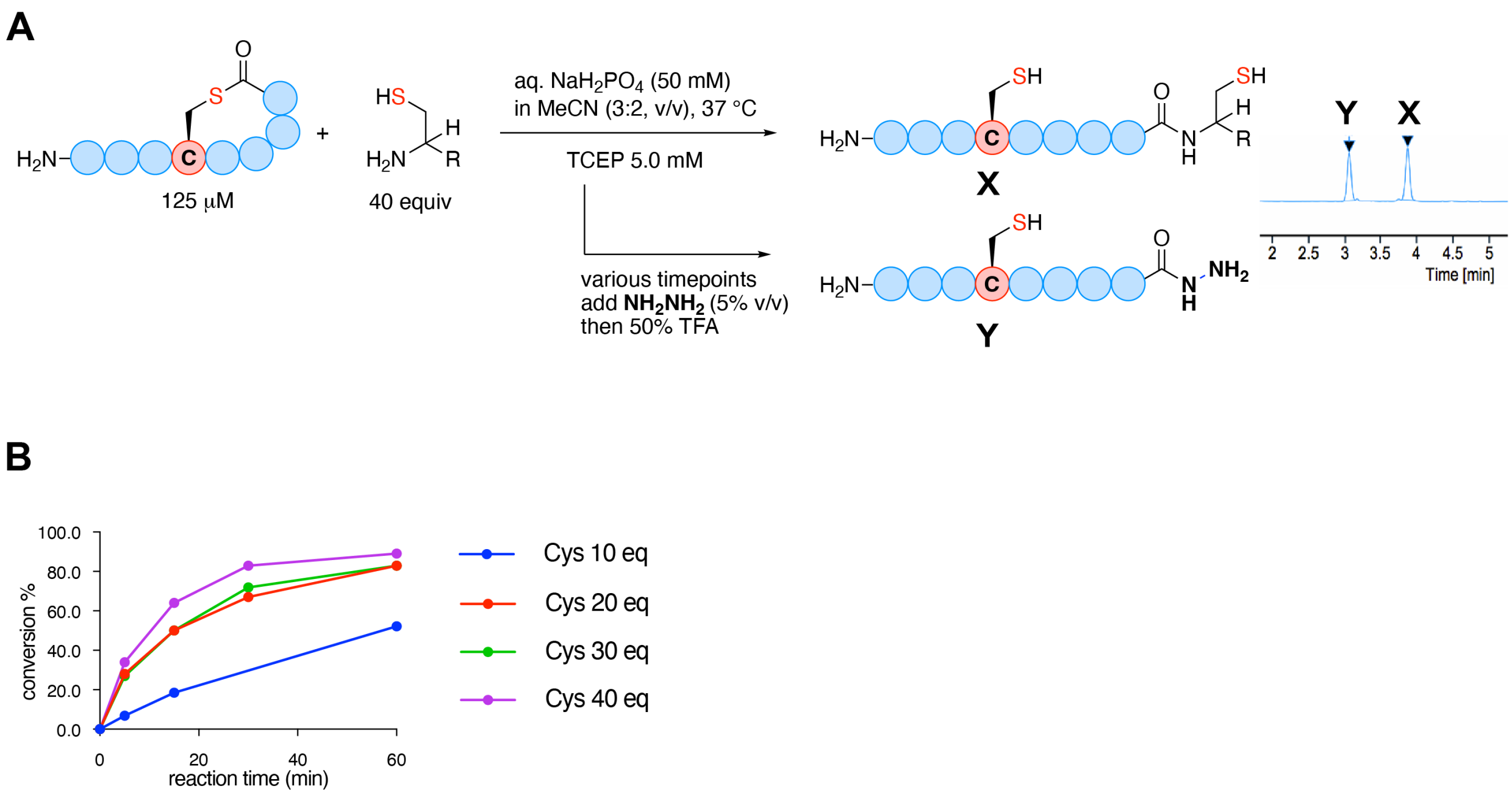


**Supporting Figure S1.** UPLC evaluation of AIP destruction by selected commercially available 2-aminothiols. A) Outline of the HPLC assay procedure. B) Destruction of the *S. aureus* AIP-I thiolactone by varying concentrations of cysteine over time.

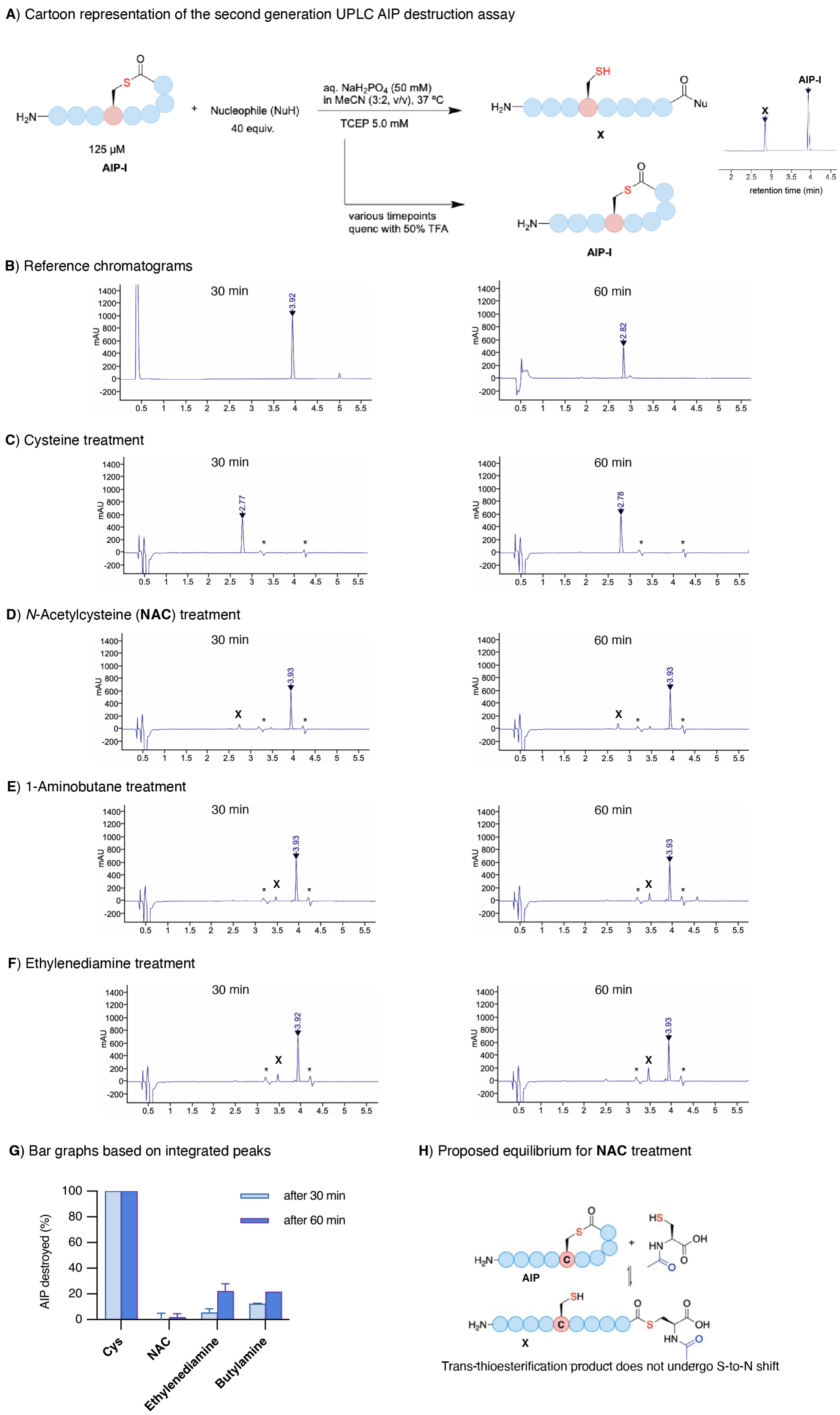


**Supporting Figure S2.** A) Cartoon representation of the second UPLC destruction assay set-up. B) Reference chromatograms of AIP-I thiolactone after incubation for 30 or 60 in buffer. B) Full conversion to linearized peptide **S13** after treatment with cysteine. C)–F) Chromatograms resulting from treatment with **NAC**, 1-aminobutane, or ethylenediamine (40 equiv) for 30 min (left) or after 60 min (right). G) Quantification of destroyed AIP based on integrals in the chromatograms. Mean ± SD, n = 2 replicates. *Artifacts resulting from our UV detector and baseline correction. H) Proposed reversible reaction of AIP with **NAC**, which cannot undergo the NCL-like S-to-N acyl shift.

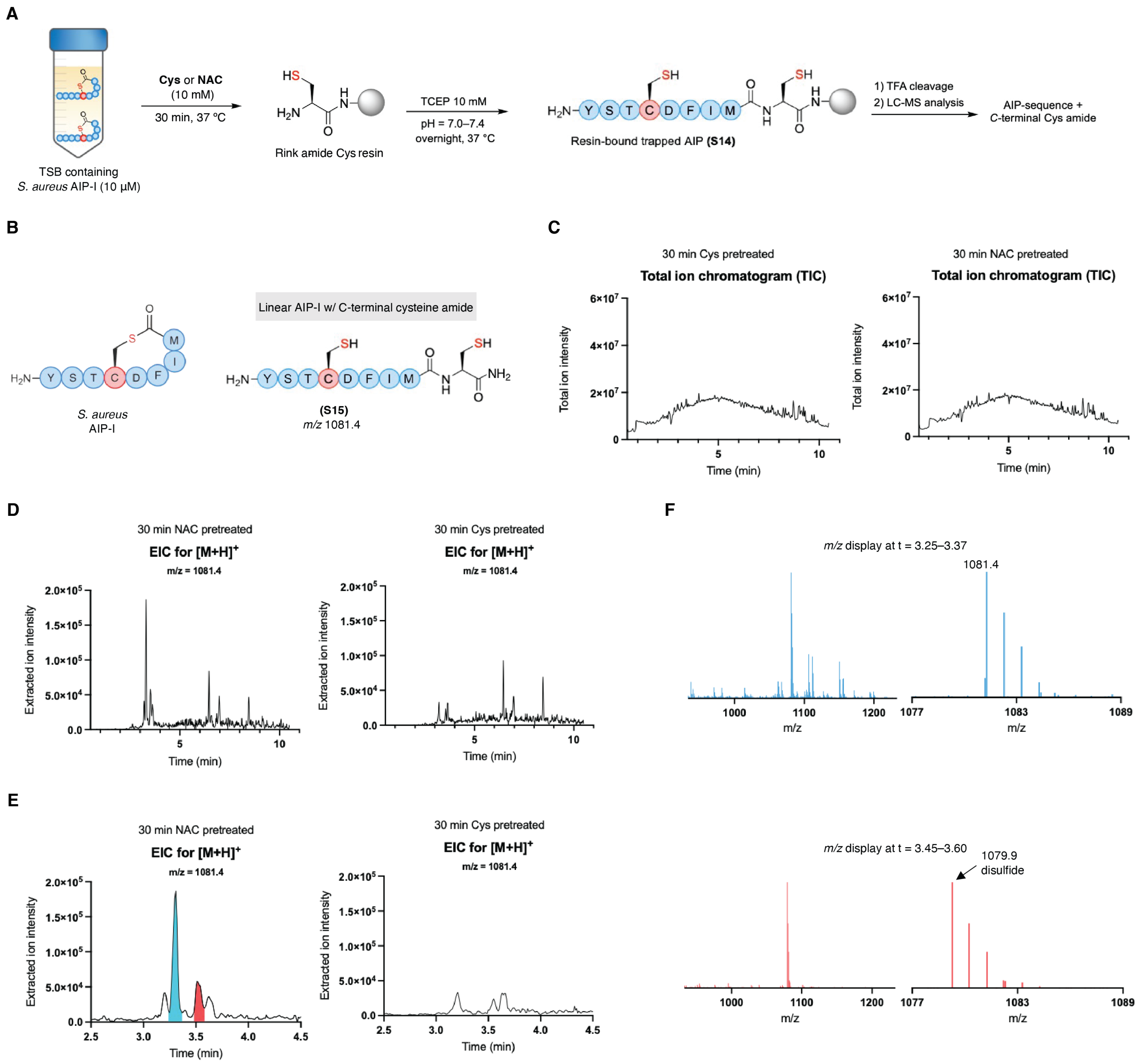


**Supporting Figure S3.** NCL trapping of *S. aureus* AIP-I from medium treated with **Cys** or **NAC**. A) TSB solution containing *S. aureus* AIP-I (10 µM) was incubated with **Cys** or **NAC** (10 mM) for 30 min at 37 ºC. The solutions were then incubated with Cys-resin and tris(2-carboxyethyl)phosphine (TCEP, 10 mM) to reduce disulfide formation for 16 hours at room temperature. The resins with the trapped AIP (**S14**) were then washed and treated with trifluoroacetic acid (TFA) to release the trapped AIP. The concentrated cleavage solutions were reconstituted in MeCN–water (1:1) and analyzed by liquid-chromatography mass-spectrometry (LC-MS). B) Structures of *S. aureus* AIP-I and the product trapped and cleaved AIP. C) LC-MS analysis of the cleavage solutions shown as TIC (total ion chromatogram). D) LC-MS analysis of the TFA cleavage solutions shown as EICs of m/z = $[M+H]^+$ of linear AIP sequence including a C-terminal cysteine amide (**S15**). E) Zoom in of the EICs. F) Mass spectrograms of the enriched blue and red peaks, appearing after **NAC** treatment but not after **Cys** treatment, showing that intact AIP can be trapped after treatment with **NAC** but not after treatment with **Cys**.

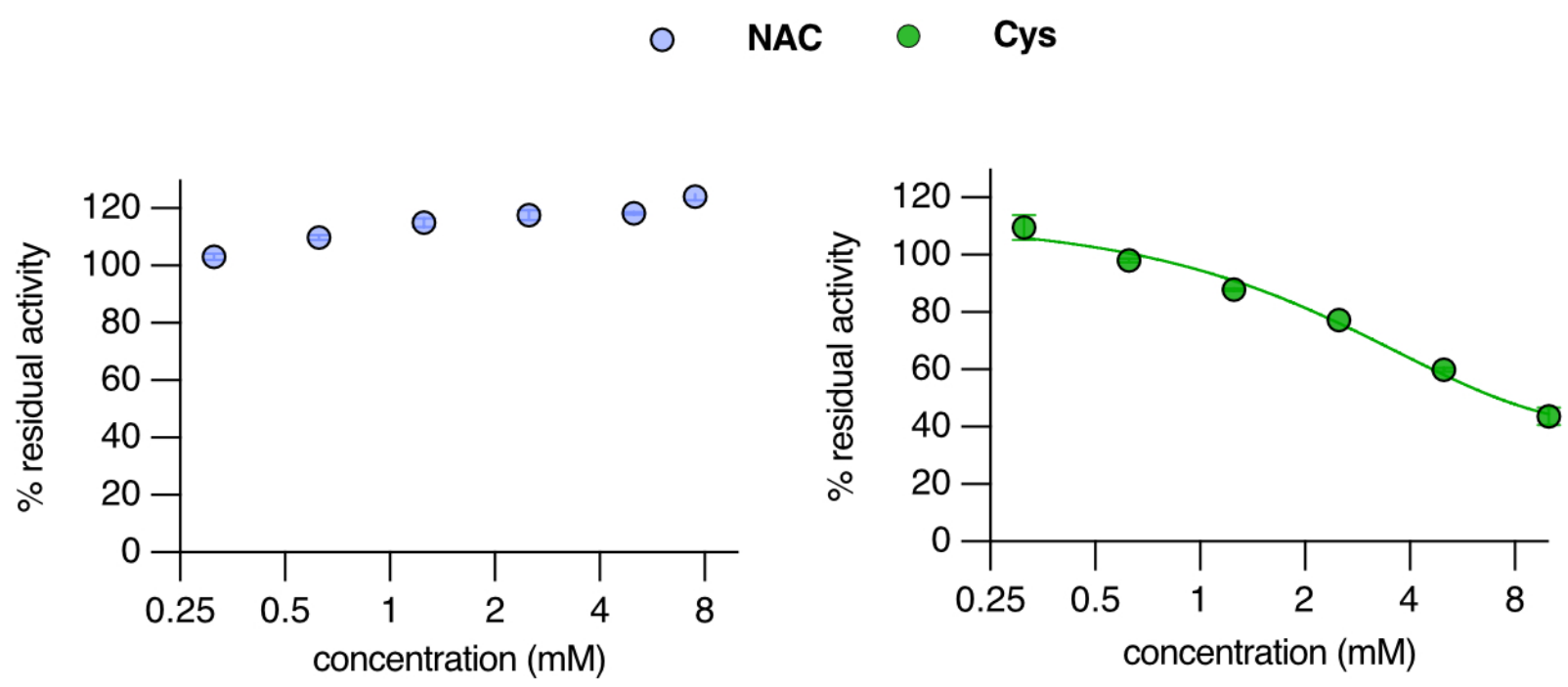


**Supporting Figure S4.** Evaluation of the inhibition of quorum sensing in P3-*yfp* reporter strain assays based on *S. aureus* specificity group *agr*-I. Dose-response curves for quorum sensing inhibition by **Cys** and **NAC.** At least two individual assays were performed in technical triplicate. Each curve represents the two biological replicates with $IC_{50}$ curves fitted by non-linear regression (variable slope) in GraphPad Prism; data points and error bars represent the mean ± SD.

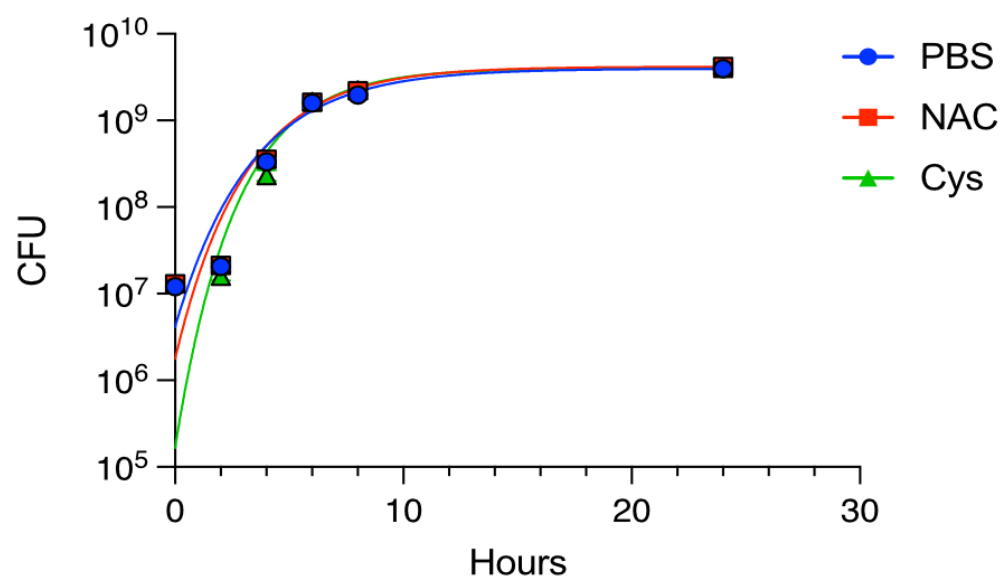


**Supplementary Figure S5. Independent CFU count for P3-yfp (*agr*-I) reporter strain.** Evaluation of how cysteine and NAC affect *S. aureus* growth. CFU counts of the P3-*yfp* (*agr*-I) reporter strain grown in TSB with PBS vehicle control, **NAC**, or **Cys** (5 mM) over 24 h. Mean ± SD, n = 3 biological replicates.

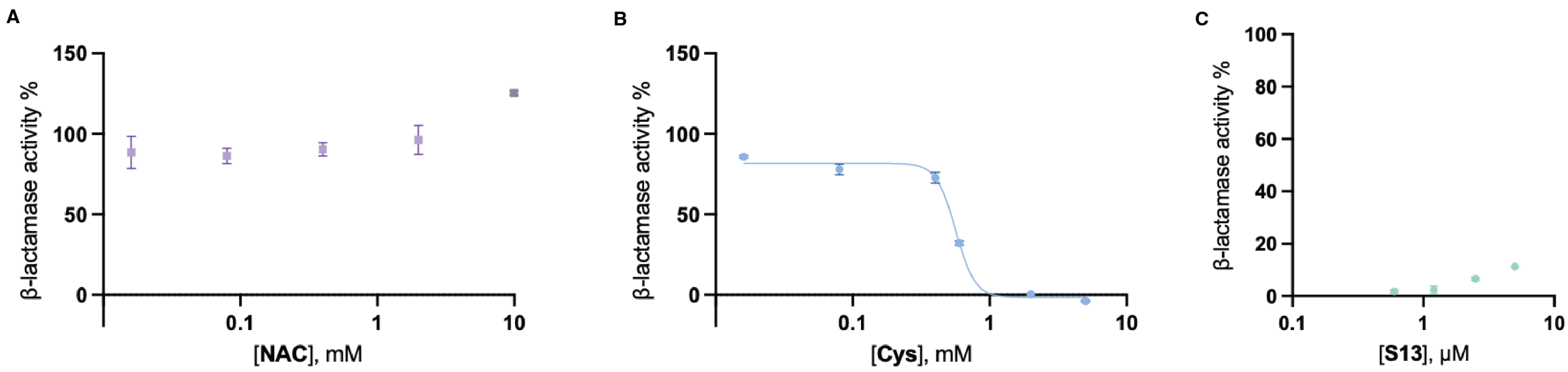


**Supplementary Figure S6.** Dose–response curves of the effect on quorum sensing after pre-incubating the activating AIP-I with varying concentrations of A) **NAC** (10 mM – 16 μM) or B) **Cys** (10 μM – 10 pM), determined in the β-lactamase activity *S. aureus* agr-I reporter strain. C) Activation properties of the linearized AIP with terminal cysteine (**S13**) (8 μM – 0.6 μM) determined through β-lactamase activity using the *S. aureus* agr-I reporter strain without native AIP-I. The curves were generated from three individual assays performed in technical duplicate and the error bars represent the standard deviation of the mean (SD).

# Materials and Methods

## Reagents and materials

Bacteria were cultured in tryptic soya broth (TSB) medium and on tryptic soya agar (TSA) plates (both Oxoid) supplemented with 10 µg/ml chloramphenicol (Sigma-Aldrich) when appropriate. *N*α-Fmoc protected and *N*α-Boc protected amino acids, 2-(1*H*-Benzotriazol-1-yl)-1,1,3,3-tetramethyluronoium hexafluorophosphate (HBTU) and 2-(1*H*-7-Azabenzotriazol-1-yl)-1,1,3,3-tetramethyluronoium hexafluorphosphate (HATU) were obtained from ChemImpex and PepChem. Aminomethyl ChemMatrix resin was obtained from PCAS BioMatrix. Aminomethyl PEGA resin, *N*,*N*-diisopropylethylamine, 4-nitrophenylchloroformate and maleic acid (qNMR grade) were obtained from Sigma-Aldrich. Nitrocefin was obtained from Oxoid. All other chemicals used were obtained in the highest available purity from CombiBlocks or IrisBiotech. All solvents used were of analytical grade and purchased from Fisher Scientific.

## Analysis and purification

Ultra-high-performance liquid chromatography (UPLC) mass spectrometry (MS) analyses were performed on a Phenomenex Kinetex column (1.7 µm, 100 Å, 50 × 2.10 mm) using a Waters Acquity ultra-HPLC system. A gradient with eluent C (0.1% HCOOH in water) and eluent D (0.1% HCOOH in MeCN) rising linearly from 0 to 95% of D over 5.20 min at a flow rate of 0.6 mL min$^{-1}$ was applied to analyse reaction mixtures. Preparative HPLC purification was performed on a C18 Phenomenex Luna column (5 µm, 100 Å, 250 × 20 mm) using an Agilent 1260 LC system equipped with a diode array ultraviolet detector. Various gradients with eluent A and eluent B at a flow rate of 20 mL min$^{-1}$ were applied for the purification. Fractions containing the purified target peptide were identified using UPLC-MS or matrix assisted laser desorption ionisation–time of flight mass spectrometry (MALDI-TOF MS). Selected fractions were pooled and lyophilized. MALDI-TOF mass spectra were recorded with a Bruker microflex bench-top MALDI using a matrix of alpha-cyano-4-hydroxycinnamic acid or 2,5-dihydroxybenzoic acid in water/MeCN (1:1, v/v) containing 0.1% TFA. The observed *m/z* corresponded to the monoisotopic ions, unless otherwise stated. High-resolution mass spectrometry (HRMS) was recorded either on a QExactive Orbitrap mass spectrometer (Thermo Scientific, Bremen, Germany) equipped with a SMALDI5 ion source (TransMIT GmbH, Giessen, Germany), or on a Bruker Solarix WR by either matrix assisted laser desorption/ionization (MALDI) or electrospray ionization (ESI). Nuclear magnetic resonance (NMR) spectra were recorded at 298 K using a Bruker Avance III HD ($^{1}$H NMR and $^{13}$C NMR recorded at 600 MHz and 150 MHz, respectively). Chemical shifts are reported in parts per million (ppm) relative to the deuterated solvent peak of DMSO-$d_6$ ($\delta_H$ = 2.50 ppm; $\delta_C$ = 39.52 ppm) as internal standard.

## HPLC assays

### Evaluation of AIP destruction by HPLC analysis

Native chemical ligation (NCL) kinetics were monitored by UPLC using reaction mixtures containing the respective Cys analogues (40 equiv) in phosphate buffer (50 mM, pH 7.4) with 40% MeCN in an Eppendorf tube at 37 °C on a water bath. At defined time points, 100 µL aliquots of the reaction mixture were withdrawn and quenched by either (Assay type-1) the addition of hydrazine solution for 5 min, followed by acidification with 50% aqueous TFA or (Assay type-2) the addition 50% aqueous TFA. Samples were then filtered (0.4 µm) and analyzed by UPLC on

a C18 Agilent InfinityLab Poroshell 120 column (2.7 µm, 100´ 3.0 mm) using an Agilent 1260 Infinity II series system equipped with a diode array UV detector. A gradient with eluent A (water–MeCN–TFA, 95:5:0.1, v/v/v) and eluent B (0.1% TFA in MeCN), rising linearly from 20–50% B over 5 min was applied at a flow rate of 1.2 mL $min^{-1}$ at 40 °C ($\lambda$ = 215 nm). Reaction progress was quantified from the ratio of peak integrals corresponding to the linear hydrazide starting material ($t_R \approx 3.0$ min for Ss-AIP) and the later-eluting ligated product. Acidified samples remained stable and could be stored prior to analysis. Stock solutions were prepared as described above and pre-warmed buffer–MeCN mixtures were used for all assays.

**Native chemical ligation (NCL) trapping**

*Sample preparation:* Synthetic AIP (SA-AIP-I) was added in 5 mL TSB to a final concentration of 10 µM. Before proceeding to native chemical ligation (NCL) trapping, the solution was preincubated with 10 mM Cys or NAC for 30 min at 37 ºC.

*Resin preparation:* for each prepared supernatant sample (~5 mL), Fmoc-Cys(*S*-*t*Bu)-Rink-PEGA resin (50 mg) was deprotected in a 5.0 mL polypropylene syringe with a fritted disk. The resin was swelled for 15 min in DMF and washed with DMF (5 × 1 min). Then, the resin was treated with piperidine in DMF (1:4, v/v, 2.0 mL) (1 × 2 min and 1 × 20 min) and washed with DMF (5 × 1 min). Next, the disulfide protecting group on cysteine was reduced with 10 mM 1,4-dithiothreitol (DTT) in DMF–$H_2O$–*i*$Pr_2$NEt (95:2.5:2.5, v/v/v, 1 mL) (3 × 10 min) and was subsequently washed with DMF (3 × 1 min), MeOH (3 × 1 min), and H2O (3 × 1 min).

*NCL trapping:* An aqueous tris(2-carboxyethyl)phosphine hydrochloride (TCEP) solution (1 mL, 0.5 M, pH 7.0) was then added, followed by the deprotected Cys-Rink-PEGA resin. The resin-containing supernatant was incubated at 37 °C overnight and the supernatant was then removed under suction in a 10 mL polypropylene syringe equipped with a fritted disk. The resin was washed with DMF (3 × 1 min), H2O (3 × 1 min), and DMF (3 × 1 min) and was subsequently treated with DTT (2 mL; 10 mM) in DMF–$H_2O$–*i*$Pr_2$NEt) (95:2.5:2.5, v/v/v) for 30 min at 37 °C, to reduce potential disulfides formed between linear peptides trapped on-resin. After the reduction, the resin was washed with DMF (3 × 1 min), MeOH (3 × 1 min), and CH2Cl2 (3 × 1 min) and was dried under suction for 15 min. The dried resin was treated with a cleavage cocktail (2.0 mL, TFA–MilliQ water, 97:3, v/v) for 2 hrs at room temperature. The cleavage solution was collected, and the resin was rinsed with neat TFA (1.0 mL). The combined TFA fractions were evaporated to near dryness under a stream of $N_2$, and the residue was reconstituted in MeCN–$H_2O$ (100 µL, 1:1, v/v) and filtered (0.22 µm).

*LC-MS analysis:* the resulting solutions were analyzed by LC-MS by applying a gradient of solvent A (0.1% HCOOH in water) and solvent B (0.1% HCOOH in MeCN), rising linearly from 0 to 100% of B over 10.0 min at a flow rate of 0.6 mL min-1 and with an injection volume of 10 µL. Total ion chromatograms (TIC) were analyzed by displaying extracted ion chromatograms (EIC) of *m/z* [M+H]+ values of the possible linear AIPs, based on the AgrD sequence with an additional C-terminal cysteine amide.

**Determination of Thiol Concentration Using DTNB (Ellman's Reagent)**

The concentrations of Cys analogue stock solutions were determined spectrophotometrically using 5,5′-dithiobis(2-nitrobenzoic acid) (DTNB, Ellman's reagent) and a calibration curve generated from *N*-acetylcysteine (NAC) standards. A 50 mM DTNB solution was prepared in phosphate buffer (0.1 M, pH 8.0), while NAC stock solutions were prepared in phosphate buffer (0.1 M, pH 7.4). Serial two-fold dilutions of NAC were prepared to generate

calibration standards ranging from 1.0 to 0.0167 mM final concentration. Standard samples were prepared by mixing phosphate buffer (0.1 M, pH 8.0), DTNB solution (final concentration 5.0 mM), and NAC solution, followed by vortexing and incubation for 1 min at room temperature. Absorbance was measured at 412 nm on a NanoDrop spectrophotometer without baseline correction. Each concentration was measured in duplicate, and the full calibration procedure was repeated independently three times.

Cys analogue stock solutions were prepared by dissolving the corresponding compounds in degassed phosphate buffer (0.1 M, pH 8.0 for hydrochloride salts or pH 7.4 for zwitterionic compounds) to an approximate concentration of 200 mM. Thiol concentrations were then determined using the DTNB assay under the same conditions as the NAC standards, and the concentrations were calculated from the corresponding calibration curve. The solutions were subsequently diluted to 100 mM with degassed buffer, aliquoted (100 µL), and stored frozen until use.

## Bacterial assays and murine skin model

### Fluorescence reporter assay for screening *agr*-interference of peptides

Peptides were evaluated for the ability to interfere with *agr*-mediated quorum sensing in *S. aureus* (AH1677, AH430, AH1747 and AH1872 for *agr*-I–IV, respectively)[5] using reporter strains expressing yellow fluorescent protein (YFP) upon *agr* activation. Overnight cultures of the reporter strains were grown in TSB medium containing chloramphenicol (CAM, 10 µg/mL) and diluted 1:100 in fresh TSB medium containing the same antibiotic. Assays were performed in sterile black 96-well plates with clear bottom. All compounds were screened at concentrations from 10 mM in two-fold dilution. DMSO stock solutions of were diluted in TSB media and added (15 µL) in technical triplicate to the 96-well plate followed by diluted bacterial overnight cultures (135 µL). Control wells for 100% *agr* activity were wells replacing the test compound solution with TSB medium (15 µL). Wells containing 150 µL TSB medium were used to measure background fluorescence. The 96-well plates were incubated in a humidified incubator at 37°C shaking at 1000 rpm for 22–24 h and fluorescence (for GFP: excitation 479 nm, emission 520 nm; for YFP: excitation 500 nm, emission 541 nm; automatic gain) and $OD_{600}$ values were subsequently measured using a plate-reader. Background fluorescence was subtracted from all wells and further normalized to the corresponding $OD_{600}$ value of the respective wells. Average fluorescence of control wells was used as relative measure for 100% activation of the *agr*-circuit and bar graphs were generated using GraphPad Prism 10.4 software. All assays were performed in at least biological duplicate.

### Fluorescence reporter assay for $IC_{50}$ determination

Overnight cultures of the reporter strains were grown in TSB medium containing chloramphenicol (CAM, 10 µg/mL for *S. aureus*) and diluted 1:100 in fresh TSB medium containing the same antibiotic. Assays were performed in sterile black 96-well plates with clear bottom. Peptide solutions (15 µL) in 1:5 serial dilutions from DMSO stock solutions (1 mM) in TSB medium were added to the 96-well plate in technical duplicate followed by diluted bacterial overnight cultures (135 µL). Control wells for 100% *agr* activity were wells replacing the peptide solution with TSB medium (15 µL). Wells containing 150 µL TSB medium were used to measure background fluorescence. The 96-well plates were incubated in a humidified incubator at 37 °C shaking at 1000 rpm for 22–24 h and fluorescence (GFP: excitation 479 nm, emission 520 nm; automatic gain) and $OD_{600}$ values were

subsequently measured using a plate-reader. Background fluorescence was subtracted from all wells and further normalized to the corresponding $OD_{600}$ value of the respective wells. Average fluorescence of control wells was used as relative measure for 100% activation of the *agr*-circuit. Relative *agr* activity was plotted to obtain $IC_{50}$ values by non-linear regression with variable slope using GraphPad Prism 10.4 software. All assays were performed in biological triplicate.

**Fluorescence reporter assay for *agr* deactivation of virulent *S. aureus***

A fluorescent reporter strain of *S. aureus* JE2 containing a plasmid with a *spa::GFP* promoter was constructed by transforming the strain with pALC1741.[6] Cultures of the reporter strain were grown from single colonies in TSB medium containing chloramphenicol (CAM, 10 µg/mL) and *S. aureus* AIP-I (100 nM or 50 nM) to $OD_{600}$ = 0.2–0.3 (early exponential phase) at 37 °C. Assays were performed in sterile black 96-well plates with clear bottom. Compound solutions (15 µL), prepared as 1:2 serial dilutions in 0.1 M $NaH_2PO_4$ buffer (pH 7.4) from 200 mM stock solutions in 0.1 M $NaH_2PO_4$ (pH 8.0) and filtered through a sterile filter, were added to 96-well plates in technical triplicate, followed by the addition of induced bacterial cultures (135 µL). Control wells were uninduced cultures containing no *S. aureus* AIP-I. The 96-well plates were incubated in a plate reader at 37 °C shaking at 283 rpm and fluorescence (GFP: excitation 479 nm, emission 520 nm; automatic gain) and $OD_{600}$ values were continuously measured over 22 hours. Final fluorescence measurements were normalized to the corresponding $OD_{600}$ value of the respective wells and the average fluorescence of control wells induced with AIP-1 and inhibited by addition of AIP-$III_{D4A}$ (100 nM) were used as relative measure for 100% GFP expression.

***In vivo* MRSA murine skin infection model**

The mouse model was performed under contract at Statens Serum Institut (DK) essentially as previously described.[7] In brief, eight to ten-week-old Balb/c female mice (Taconic Denmark) mice were used for all experiments [n = 16 for vehicle, n = 8 for Fucidin® (2% fusidic acid ointment) treatment, and n = 8 for test compounds **CA** and **11**]. All animal experiments were approved by the National Committee of Animal Ethics, Denmark. Mice were anaesthetized and the hair was removed on a 2 $cm^2$ skin area on the back and thereafter was the outermost layer of the skin scraped off with a dermal curette to obtain a 1 $cm^2$ superficial skin lesion. For vehicle control and Fucidin® treatment, 10 µL inoculum containing approximately $10^7$ CFU of methilicin-resistant *S. aureus* (MRSA43484) were spread on the skin lesions. For test compound treatments, 10 µL of the same inoculum containing **CA** or **11** (10 mM prepared from a 100 mM DMSO stock solution of **CA**·HCl or **11**·HCl immediately before application) were spread on the skin lesions. After the applied inoculum had dried, the mice were placed in a cage and kept in a warming cabinet until fully awake. The topical skin treatment with Fucidin® was initiated one day after inoculation (day 2, 3, 4) by spreading 50 µL of Fucidin® on the inoculated skin area once a day. Skin lesion size and body weight was measured on day 1, 2, and 4 of all mice. Statistical testing was conducted using one-way analysis of variance (ANOVA) with multiple comparisons (Dunnett's test) and alpha level 0.05.

**β-Lactamase assay for $IC_{50}$ determination against *S. aureus* agr-I**

The β-lactamase reporter strain RN10829 (P2-agrA:P3-blaZ), with pagrC-I, substituting the native agr locus with a chromosomal integration of P2-agrA and P3-blaZ and a plasmid from which a wild-type variant of the corresponding AgrC is expressed, was used to assess inhibition and activation of the AgrC receptor via β-lactamase activity in response to varying concentrations of the QS modulating small molecules. Overnight cultures of the

reporter strains in TSB medium were diluted 1:250 in fresh TSB medium and grown to $OD_{600}$= 0.35–0.40 (early exponential phase) at 37 °C. Solutions of **Cys** or **NAC** (10 µL) in 1:2 serial dilutions from phosphate buffer stock solutions (100 mM) in TSB medium (final concentrations = 10 mM–0.6 mM) were preincubated with cognate AIP (1:1) from a 10 µM TBS stock (DMSO <1%) for 30 min, except for the testing of activation by **S13**, where no additional AIP activator was added. After the incubation, the solutions were added to each well of a clear 96-well plate (20 µL) followed by 80 µL of bacterial cells. Control wells for 100% β-lactamase activity were wells replacing **NAC/Cys** solutions with TSB medium (10 µL), and control wells for 0% β-lactamase activity were wells replacing both peptide and cognate AIP solutions with TSB medium (20 µL). The 96-well plates were incubated at 37 °C shaking at 1100 rpm for 1 h and immediately frozen down at −80 °C to minimize growth during nitrocefin treatment. Next, the 96-well plates were thawed, and $OD_{600}$ values were determined using a plate reader followed by the addition of 50 µL of nitrocefin solution to the wells (final concentration = 33.3 µg/mL). β-Lactamase activity was monitored at $OD_{486}$ every 35 s for 30 min at 37 °C using a plate-reader. Linear nitrocefin conversion rates were plotted to obtain $IC_{50}$ values by non-linear regression with variable slope using GraphPad Prism 10.4 software.

**Independent measurement of cell growth**

For independent measurement of cell growth and viability in presence of cysteine and NAC, we grew the P3-*yfp* (*agr*-I) reporter strain in presence of the compounds in a plate-based setup as used for monitoring QS activity. Briefly, overnight cultures of the reporter strain were diluted 100x in fresh TSB and dispensed into wells containing **Cys** or **NAC** (5 mM final concentration) including a buffer (PBS) control in two technical replicates. The microtiter plate was incubated in a humidified incubator at 37 °C, 1000 rpm. CFU counts were retrieved at 0, 2, 4, 6, 8, and 24 hours by ten-fold serial dilution in 0.9% NaCl, spotting on TSA and colony counting after 16 hours of growth at 37 °C. Three biological replicates were included in the viability assays.

## Synthesis of protected 2-amino-thiol precursors

**Benzyl *N*-(*tert*-butoxycarbonyl)-*S*-trityl-*L*-cysteinate (S1).** Boc-*L*-Cys(Trt)-OH (927 mg, 2.00 mmol, 1.00 equiv.) and *i*-$Pr_2NEt$ (1.05 mL, 6.00 mmol, 3.00 equiv.) were dissolved in anhydrous $CH_2Cl_2$ (20 mL) cooled to 0 °C using an ice/water bath. Benzyl bromide (0.71 mL, 6.00 mmol, 3.00 equiv.) was added to the stirred solution and the reaction mixture was allowed to warm to room temperature and stirred overnight. Then the mixture was concentrated under reduced pressure, and the remaining residue was dissolved in EtOAc (30 mL). The organic phase was washed with 5% citric acid solution (2 × 40 mL), saturated $NaHCO_3$ solution (2 × 40 mL) and brine (2 × 40 mL), dried over anhydrous $MgSO_4$, filtered and concentrated under reduced pressure. The crude product was purified by flash column chromatography (Hep/EtOAc = 4/1) to afford the compound as white foam (662 mg, 1.20 mmol, 60%), which was used without further purification.

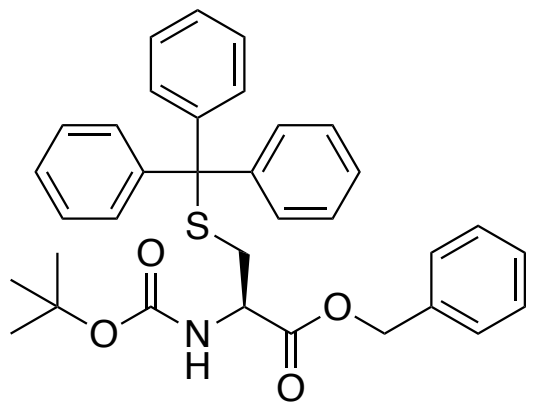

**2-Amino-2-oxoethyl *N*-(*tert*-butoxycarbonyl)-*S*-trityl-*L*-cysteinate (S2).** Boc-*L*-Cys(Trt)-OH (927 mg, 2.00 mmol, 1.00 equiv.) and *i*-$Pr_2NEt$ (1.05 mL, 6.00 mmol, 3.00 equiv.) were dissolved in anhydrous $CH_2Cl_2$ (20 mL) cooled to 0 °C using an ice/water bath. Iodoacetamide (1.11 g, 6.00 mmol, 3.00 equiv.) was added to the stirred solution and the reaction mixture was allowed to warm to room temperature and stirred overnight. Then the mixture was concentrated under reduced presure, sand the remaining residue was dissolved in EtOAc (30 mL). The organic phase was washed with 5% citric acid solution (2 × 40 mL), saturated $NaHCO_3$ solution (2 × 40 mL) and brine (2 × 40 mL), dried over anhydrous $MgSO_4$, filtered and concentrated under reduced pressure. The crude product was purified by flash column chromatography (Hep/EtOAc = 2/1) to afford the compound as white foam (287 mg, 0.55 mmol, 28%), which was used without further purification.

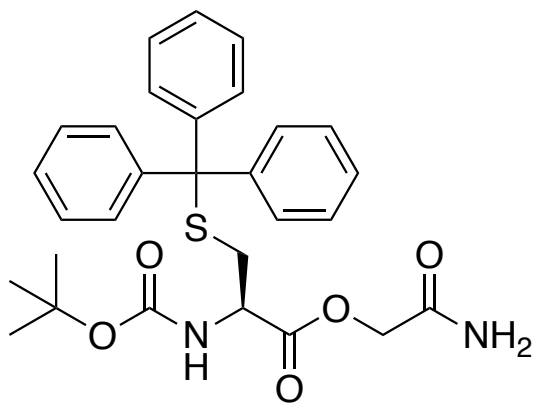

**Cyclohexyl *N*-(*tert*-butoxycarbonyl)-*S*-trityl-*L*-cysteinate (S3).** Boc-L-Cys(Trt)-OH (927 mg, 2.00 mmol, 1.00 equiv.), cyclohexanol (425 mg, 2.20 mmol, 1.10 equiv.) and 4-dimethylaminopyridine (DMAP) (41.5 mg, 0.34 mmol, 0.17 equiv.) were dissolved in anhydrous $CH_2Cl_2$ (20 mL) and cooled to 0 °C using an ice/water bath. EDC·HCl (460 mg, 2.40 mmol, 2.40 equiv.) was added to the stirred solution and the reaction mixture was allowed to warm to room temperature and stirred overnight. Then the mixture was concentrated under reduced pressure and the remaining residue was dissolved in EtOAc (30 mL). The organic phase was washed with 5% citric acid solution (2 × 40 mL), saturated $NaHCO_3$ solution (2 × 40 mL) and brine (2 × 40 mL), dried over anhydrous $MgSO_4$ and filtered. Concentration under reduced pressure afforded the compound as white foam (830 mg, 1.52 mmol, 76%), which was used without further purification.

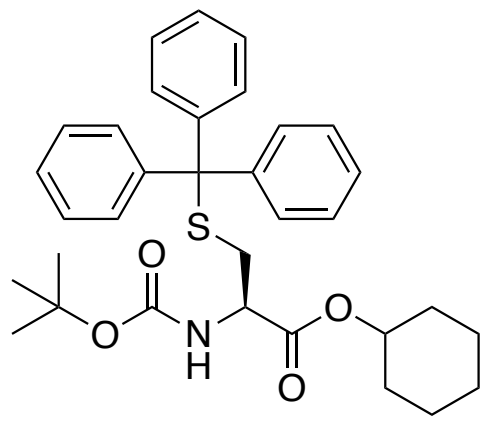

***tert*-Butyl (*R*)-(1-hydroxy-3-(tritylthio)propan-2-yl)carbamate (S4).** $K_2CO_3$ (2.07 g, 15.0 mmol, 3.00 equiv.) was added to a solution of Boc-*L*-Cys(Trt)-OH (2.32 g, 5.00 mmol, 1.00 equiv.) in anhydrous DMF (17 mL) and the slurry was stirred for 1.5 h. Iodomethane (1.39 mL, 15.0 mmol, 3.00 equiv.) was added dropwise and the reaction mixture was stirred overnight. After confirmed reaction completion via thin-layer chromatography (TLC) (0.1% AcOH in heptane–EtOAc = 3:1 or 1:1), the reaction mixture was concentrated under reduced pressure, and the remaining residue was dissolved in EtOAc (50 mL). The organic phase was washed with water (50 mL) and the aqueous phase back extracted with EtOAc (3 × 50 mL). The combined organic layers were washed with aqueous 5% $Na_2SO_3$ solution (3 × 100 mL), brine (250 mL), dried over anhydrous $MgSO_4$, filtered, and concentrated under reduced pressure. The crude methyl ester and LiCl (530 mg, 12.5 mmol, 2.50 equiv.) were dissolved in anhydrous THF (15 mL) and cooled to 0 °C using an ice/water bath. $NaBH_4$ (472.5 mg, 12.5 mmol, 2.50 equiv.) was added to the stirred solution and after 15 min EtOH (20 mL) was added to the reaction mixture, which was stirred for an additional 1 h at 0 °C. The reaction mixture was allowed to warm to room temperature and stirred for 3 h after which more $NaBH_4$ was added (472.5 mg, 12.5 mmol, 2.50 equiv.) and the reaction mixture was stirred overnight. After confirmed reaction completion via TLC (heptane–EtOAc = 3:1 or 1:1), water (7.5 mL) was added followed by careful addition ($H_2$ release) of statured $NH_4Cl$ solution (20 mL) and EtOAc (50 mL). The aqueous and organic layers were separated and the aqueous layer was back extracted with EtOAc (3 × 50 mL). The combined organic layers were washed with brine (250 mL), dried over anhydrous $MgSO_4$ and filtered. Concentration under reduced pressure afforded the amino

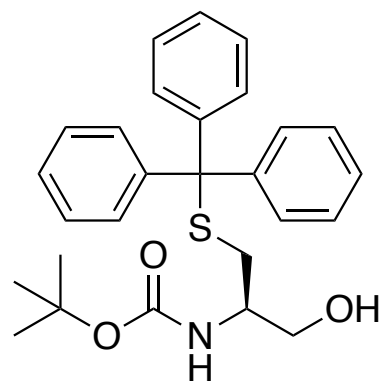

alcohol as an off-white foam (2.16 g, 4.81 mmol, 96%), which was used without further purification. $^1$H NMR (400 MHz, $CDCl_3$) $\delta$ = 1.34 (s, 9H), 2.28 – 2.41 (m, 2H), 3.35 – 3.47 (m, 3H), 4.54 – 4.68 (m, 1H), 7.11 – 7.24 (m, 10H), 7.30–7.39 (m, 5H). Data are in accordance with previous report.[2]

**General procedure A: EDC/HOBt-mediated amidation (Scheme S3).** Boc-*L*-Cys(Trt)-OH (927 mg, 2.00 mmol, 1.00 equiv.), 1-hydroxybenzotriazole (HOBt) (295 mg, 2.20 mmol, 1.10 equiv.), 1-ethyl-3-(3-dimethylaminopropyl)carbodiimide-hydrochloride (EDC·HCl) (422 mg, 2.20 mmol, 1.10 equiv.) and amine (2.20 mmol, 1.10 equiv.) were dissolved in anhydrous $CH_2Cl_2$ (20 mL) and cooled to 0 °C using an ice/water bath. *i*-$Pr_2NEt$ (1.05 mL, 6.00 mmol, 3.00 equiv.) was added to the stirred solution and the reaction mixture was allowed to warm to room temperature over 30 min and stirred overnight. Then the mixture was concentrated under reduced pressure and the remaining residue dissolved in EtOAc (30 mL). The organic phase was washed with 1 M $KHSO_4$ solution (2 × 40 mL), saturated $NaHCO_3$ solution (2 × 40 mL), brine (2 × 40 mL), dried over anhydrous $MgSO_4$, filtered and concentrated under reduced pressure. The crude cysteine amide was either used without further purification or purified by flash column chromatography or automated column chromatography.

**General procedure B: $POCl_3$-mediated amidation (Scheme S3).** Boc-*L*-Cys(Trt)-OH (927 mg, 2.00 mmol, 1.00 equiv.) and amine (2.20 mmol, 1.10 equiv.) were dissolved in anhydrous THF (20 mL) and the solution to 0 °C using an ice/water bath. The to stirred solution was added pyridine (1.60 mL, 20.0 mmol, 10.0 equiv.), followed by dropwise addition of $POCl_3$ (0.75 mL, 8.00 mmol, 4.00 equiv.). The reaction mixture was allowed to warm to room temperature over 30 min and was stirred for additional 90 min. The reaction mixture was then the cooled using an ice/water bath and saturated $NaHCO_3$ (25 mL) solution was added slowly. The reaction mixture was allowed to warm to room temperature and concentrated to approx. 25 mL under reduced pressure. The resulting mixture was extracted with EtOAc (4 × 15 mL) and the combined organic layers were washed with 0.5 M HCl solution (3 × 25 mL) and saturated $NaHCO_3$ solution (3 × 25 mL). The combined aqueous layers were extracted with EtOAc (2 × 15 mL) and the combined organic layers were washed with brine (60 mL), dried over anhydrous $MgSO_4$, filtered and concentrated under reduced pressure. The crude cysteine amide was either used without further purification or purified by a short silica gel flash column chromatography.

***tert*-Butyl (*R*)-(1-(benzylamino)-1-oxo-3-(tritylthio)propan-2-yl)carbamate (S5).** The protected cysteine analogue was prepared according to the general procedure (A) for EDC/HOBt-mediated amidation using benzyl amine (0.24 mL, 2.20 mmol, 1.10 equiv.) as amine reagent. The crude product was purified by flash column chromatography (Hep/EtOAc = 2/1) to afford the compound as white foam (795 mg, 1.44 mmol, 72%). Major conformer (conformers observed in a ratio of 1:0.25) $^1$H NMR (400 MHz, $CDCl_3$) $\delta$ = 1.37 (s, 9H), 2.54 (dd, $J$ = 13.0, 5.3 Hz, 1H), 2.74 (dd, $J$ = 13.0, 7.0 Hz, 1H), 3.80 – 3.90 (m, 1H), 4.31 – 4.40 (m, 2H), 4.77 (s, 1H), 6.20 – 6.29 (m, 2H), 7.16–7.31 (m, 15H), 7.37–7.41 (m, 5H). Data are in accordance with previous report.[3]

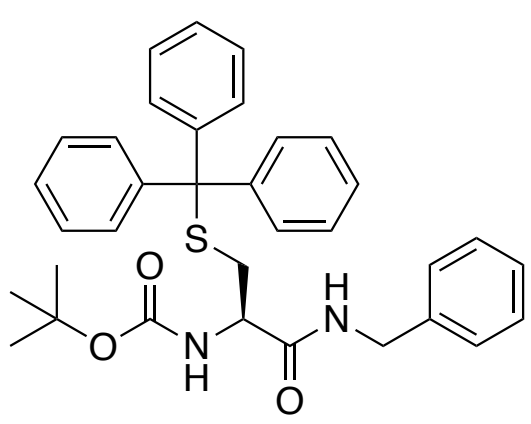

***tert*-Butyl (*R*)-(12-oxo-16,16,16-triphenyl-2,5,8-trioxa-15-thia-11-azahexadecan-13-yl)carbamate (S6).** The protected cysteine analogue **Sxx** was prepared according the general procedure (A) for EDC/HOBt-mediated amidation using 2-(2-(2-methoxyethoxy)ethoxy)ethan-1-amine (359 mg, 2.20 mmol, 1.10 equiv.) as amine reagent. The crude product was purified by automated column chromatography ($SiO_2$, gradient: Hep/EtOAc = 5/1 to 2/1) to afford the compound as a colorless oil (825 mg, 1.36 mmol, 68%). HRMS (ESI/Q-TOF) *m/z*: $[M+H]^+$ Calcd for $C_{34}H_{45}N_2O_6S^+$ 609.2993; found. Major conformer (conformers observed in a ratio of 1:0.35) $^1$H NMR (400 MHz, $CDCl_3$) $\delta$= 1.32 (s, 9H), 2.43 (dd, $J$ = 12.6, 5.4 Hz, 1H), 2.54 – 2.63 (m, 1H), 3.25 – 3.33 (m, 4H), 3.34 – 3.56 (m, 10H), 3.75 – 3.88 (m, 1H), 4.79 – 4.88 (m, 1H), 6.36 (t, $J$ = 5.5 Hz, 1H), 7.09 – 7.35 (m, 15H).

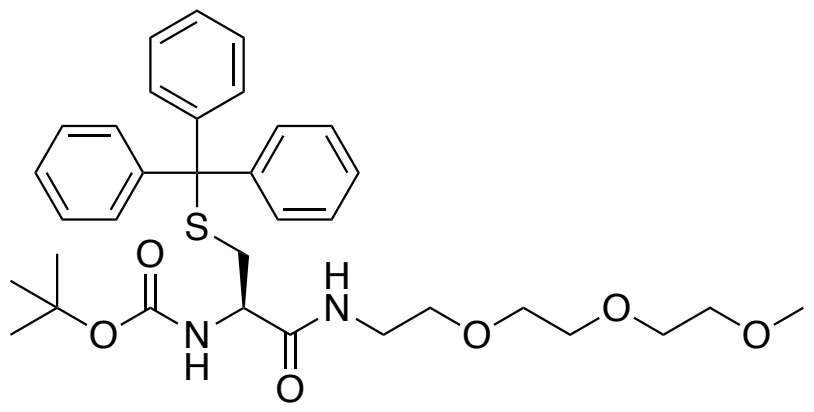

***tert*-Butyl (*R*)-(1-(butylamino)-1-oxo-3-(tritylthio)propan-2-yl)carbamate (S7).** The protected cysteine analogue was prepared according to the general procedure (A) for general procedure (A) for EDC/HOBt-mediated amidation using *n*-butylamine (0.18 mL, 2.20 mmol, 1.10 equiv.) as amine reagent. Aqueous work-up afforded the compound as white foam (899 mg, 1.73 mmol, 87%), which was used without further purification. Major conformer (conformers observed in a ratio of 1:0.33) $^1$H NMR (400 MHz, $CDCl_3$) $\delta$= 0.89 (t, $J$ = 7.3 Hz, 3H), 1.23 – 1.46 (m, 4H), 1.41 (s, 9H), 2.51 (dd, $J$ = 12.8, 5.5 Hz, 1H), 2.71 (dd, $J$ = 12.8, 7.1 Hz, 1H), 3.13 – 3.22 (m, 2H), 3.77 – 3.86 (m, 1H), 4.74 – 4.84 (m, 1H), 5.87 – 5.98 (m, 1H), 71.9 – 7.44 (m, 15H).

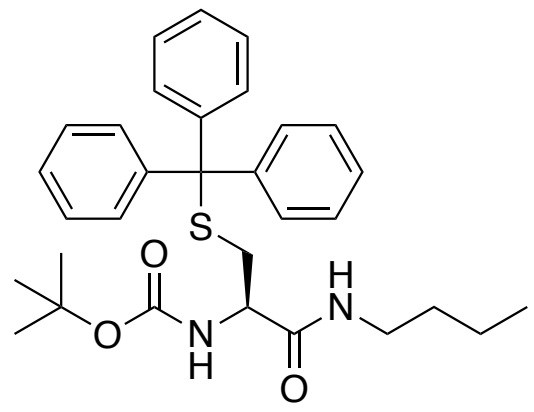

***tert*-Butyl (*R*)-(1-oxo-1-(piperidin-1-yl)-3-(tritylthio)propan-2-yl)carbamate (S8).** The protected cysteine analogue was prepared according to the general procedure (A) for EDC/HOBt-mediated amidation using piperidine (0.22 mL, 2.20 mmol, 1.10 equiv.) as amine reagent. The crude product was purified by flash column chromatography (Hep/EtOAc = 3/1) to afford the compound as white foam (905 mg, 1.71 mmol, 85%). $^1$H NMR (400 MHz, $CDCl_3$) $\delta$= 1.24 – 1.35 (m, 1H), 1.42 (s, 9H), 1.44 – 1.52 (m, 2H), 1.51 – 1.60 (m, 2H), 2.35 (dd, $J$ = 12.3, 7.4 Hz, 1H), 2.48 (dd, $J$ = 12.3, 5.1 Hz, 1H), 3.05 (m, 1H), 3.16 (m, 1H), 3.42 – 3.51 (m, 2H), 4.53 – 4.60 (m, 1H), 5.36 (d, $J$ = 8.8 Hz, 1H), 7.16 – 7.22 (m, 3H), 7.23 – 7.30 (m, 6H), 7.37 – 7.42 (m, 6H). Data are in accordance with previous report.[8]

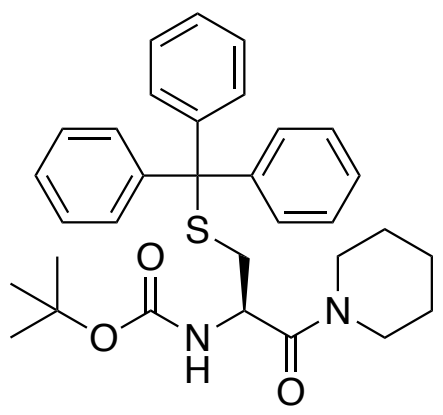

***tert*-Butyl (*R*)-(1-oxo-1-(phenylamino)-3-(tritylthio)propan-2-yl)carbamate (S9).** The protected cysteine analogue was prepared according to the general procedure (A) for EDC/HOBt-mediated amidation using aniline (0.20 mL, 2.20 mmol, 1.10 equiv.) as amine reagent. Aqueous work-up afforded the compound as white foam (930 mg, 1.73 mmol, 87%), which was used without further purification. Major conformer (conformers observed in a ratio of 1:0.15) $^1$H NMR (400 MHz, $CDCl_3$) $\delta$= 1.43 (s, 9H), 2.60 – 2.72 (m, 1H), 2.80 (dd, $J$ = 13.3, 7.2 Hz, 1H), 3.86 – 3.97 (m, 1H), 4.83 (d, $J$ = 7.5 Hz, 1H), 7.05 – 7.16 (m, 1H), 7.19 – 7.36 (m, 13H), 7.39 – 7.48 (m, 6H), 7.95 – 8.10 (m, 1H). Data are in accordance with previous report.[4]

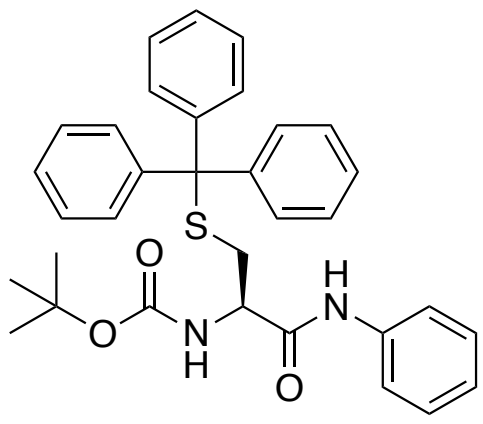

***tert*-Butyl (*R*)-4-(2-((*tert*-butoxycarbonyl)amino)-3-(tritylthio)propanamido)benzoate (S10).** The protected cysteine analogue was prepared according to the general procedure (B) for $POCl_3$-mediated amidation using *tert*-butyl 4-aminobenzoate (425 mg, 2.20 mmol, 1.10 equiv.) as amine reagent. Aqueous work-up afforded the compound as off-white foam (1.03 g, 1.61 mmol, 81%), which was used without further purification. Major conformer (conformers observed in a ratio of 1:0.15) $^1$H NMR (400 MHz, $CDCl_3$) $\delta$ = 1.43 (s, 9H), 1.58 (s, 9H), 2.66 (dd, $J$ = 13.2, 5.4 Hz, 1H), 2.80 (dd, $J$ = 13.2, 7.2 Hz, 1H), 3.87 – 3.97 (m, 1H), 4.84 (d, $J$ = 7.3 Hz, 1H), 7.20 – 7.34 (m, 10H), 7.41 – 7.47 (m, 5H), 7.50 (d, $J$ = 8.8 Hz, 2H), 7.91 (d, $J$ = 8.8 Hz, 2H), 8.38 (s, 1H).

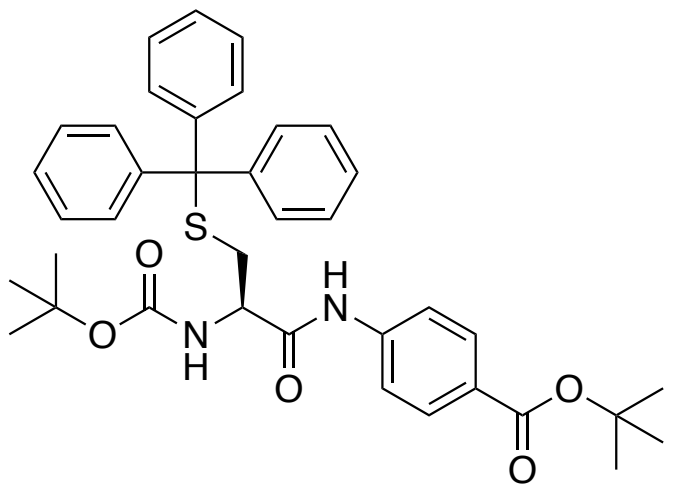

***tert*-Butyl (*R*)-(1-oxo-1-((3,4,5-trimethoxyphenyl)amino)-3-(tritylthio)propan-2-yl)carbamate (S11).** The protected cysteine analogue was prepared according to the general procedure (A) for EDC/HOBt-mediated amidation using 3,4,5-trimethoxyaniline (403 mg, 2.20 mmol, 1.10 equiv.) as amine reagent. The crude product was purified by automated column chromatography ($SiO_2$, gradient: Hep/EtOAc = 5/1 to 2/1) to afford the compound as an off-white foam (331 mg, 0.53 mmol, 26%). Major conformer (conformers observed in a ratio of 1:0.14) $^1$H NMR (400 MHz, $CDCl_3$) $\delta$ = 1.36 (s, 9H), 2.59 (dd, $J$ = 13.3, 5.4 Hz, 1H), 2.71 (dd, $J$ = 13.3, 7.2 Hz, 1H), 3.71 (s, 3H), 3.74 (s, 6H), 3.80 – 3.88 (m, 1H), 4.72 (s, 1H), 6.67 (s, 2H), 7.11 – 7.46 (m, 15H), 8.06 (s, 1H).

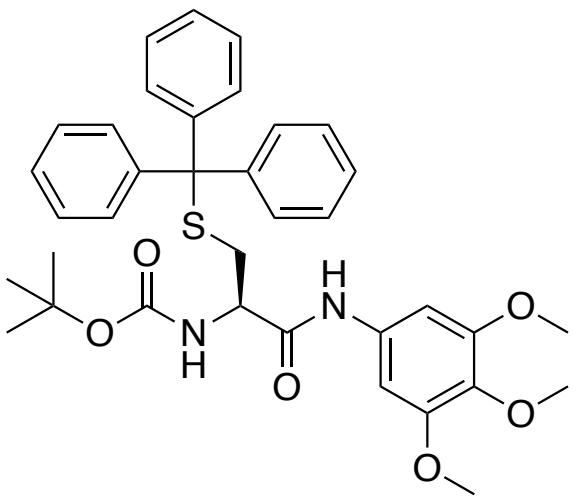

***tert*-Butyl (*R*)-(1-amino-1-oxo-3-(tritylthio)propan-2-yl)carbamate (S12).** The protected cysteine analogue was prepared according to the general procedure (A) for EDC/HOBt-mediated amidation using ammonium carbonate (576 mg, 6.00 mmol, 3.00 equiv.) as amine reagent. Aqueous work-up afforded the compound as white foam (795 mg, 1.72 mmol, 86%), which was used without further purification. $^1$H NMR (400 MHz, $CDCl_3$) $\delta$ = 1.38 (s, 9H), 2.33 (d, $J$ = 6.9 Hz, 2H), 3.93 (q, $J$ = 7.3 Hz, 1H), 6.81 (d, $J$ = 8.6 Hz, 1H), 7.05 (s, 1H), 7.18 (s, 1H), 7.22 – 7.40 (m, 15H).

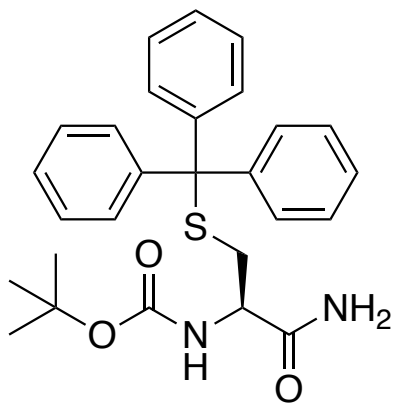

## Synthesis of hydrochloride salts of 2-aminothiol compounds

**General procedure C: TFA-mediated deprotection and hydrochloride salt formation (Scheme S4).** To a stirred solution of protected 2-amino-thiol **S1–S12** (0.5 mmol, 1.00 equiv.) and triisopropylsilane (1.02 mL, 5.00 mL, 10.0 equiv.) in $CH_2Cl_2$ (8.0 mL) was dropwise added trifluoroacetic acid (TFA) (1.15 mL, 15.0 mmol, 30.0 equiv.) and the reaction mixture was stirred at room temperature for 1 hour. After full consumption of the starting material, was the solution reduced to approx. 1.0–1.5 mL using a stream of $N_2$. To the remaining residue was added water (10 mL) followed by diethyl ether (10 mL) and the phases were separated. The aqueous layer was filtered through a syringe filter (0.43 μm) and purified by preparative RP-HPLC (gradient: eluent B 0% to 95% over 20 min, C18). The fractions containing the desired 2-amino-thiol were pooled and lyophilized. The lyophilized TFA salt was dissolved in 1 M HCl solution (3 mL) and lyophilized again to afford the hydrochloride salt of the desired 2-amino-thiol compound.

**Benzyl *L*-cysteinate hydrochloride (1).** The hydrochloride salt was prepared according to the general procedure (C) for TFA-mediated deprotection and hydrochloride salt formation starting from protected 2-amino thiol **S1** (277 mg, 0.50 mmol, 1.00 equiv.). HPLC purification and subsequent lyophilization from HCl afforded the compound as white solid (73.3 mg, 0.30 mmol, 60% for hydrochloride salt). HRMS *m/z*: $[M+H]^+$ Calcd for $C_{10}H_{14}NO_2S^+$: 212.0740; found: 212.0739. $^1$H NMR (400 MHz, DMSO-*d₆*) $\delta$ = 2.96 – 3.14 (m, 2H), 4.41 – 4.46 (m, 1H), 5.26 (s, 2H), 7.30 – 7.50 (m, 5H), 8.75 (br s, 3H). $^{13}$C NMR (101 MHz, DMSO-$d_6$) $\delta$ = 24.2, 53.9, 67.3, 128.2 (2C), 128.36, 128.44 (2C), 135.1, 167.6.

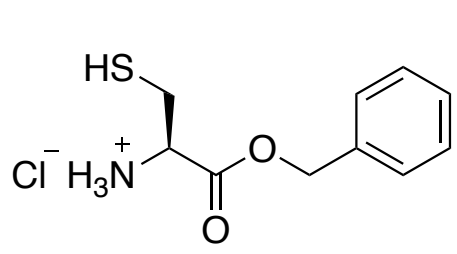

**2-Amino-2-oxoethyl *L*-cysteinate hydrochloride (2).** The hydrochloride salt was prepared according to the general procedure (C) for TFA-mediated deprotection and hydrochloride salt formation starting from protected 2-amino thiol **S2** (260 mg, 0.50 mmol, 1.00 equiv.). HPLC purification and subsequent lyophilization from HCl afforded the compound as white solid (79.4 mg, 0.37 mmol, 74% for hydrochloride salt). HRMS *m/z*: $[M+H]^+$ Calcd for $C_5H_{11}N_2O_3S^+$: 179.0485; found: 179.0486. $^1$H NMR (400 MHz, DMSO-$d_6$) $\delta$ = $^1$H NMR (400 MHz, DMSO-$d_6$) δ 3.01 – 3.16 (m, 2H), 3.21 (t, *J* = 8.8 Hz, 1H), 4.40 – 4.49 (m, 1H), 4.59 (d, *J* = 1.8 Hz, 3H), 7.43 (s, 1H), 7.71 (s, 1H), 8.84 (br s, 3H). $^{13}$C NMR (101 MHz, DMSO-$d_6$) $\delta$ = 24.8, 54.3, 63.7, 167.6, 168.4.

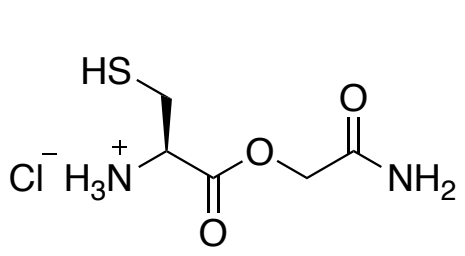

**Cyclohexyl *L*-cysteinate hydrochloride (3).** The hydrochloride salt was prepared according to the general procedure (C) for TFA-mediated deprotection and hydrochloride salt formation starting from protected 2-aminothiol **S3** (273 mg, 0.50 mmol, 1.00 equiv.). HPLC purification and subsequent lyophilization from HCl afforded the compound as white solid (96.0 mg, 0.40 mmol, 80% for hydrochloride salt). HRMS *m/z*: $[M+H]^+$ Calcd for $C_9H_{18}NO_2S^+$: 204.1053; found: 204.1054. $^1$H NMR (400 MHz, DMSO-$d_6$) $\delta$ = 1.23 – 1.41 (m, 3H), 1.42 – 1.55 (m, 3H), 1.62 – 1.74 (m, 2H), 1.74 – 1.85 (m, 2H), 2.92 – 3.17 (m, 3H), 4.29 – 4.35 (m, 1H), 4.83 (tt, *J* = 8.2, 3.7 Hz, 0H), 8.73 (br s, 3H). $^{13}$C NMR (101 MHz, DMSO-$d_6$) $\delta$ = 22.7 (2C), 24.3, 24.7, 30.7, 30.7, 53.8, 74.2, 167.1.

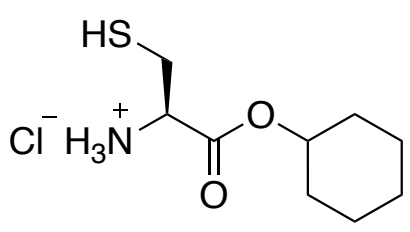

**(*R*)-2-Amino-3-mercaptopropan-1-ol hydrochloride (4).** The hydrochloride salt was prepared according to the general procedure (C) for TFA-mediated deprotection and hydrochloride salt formation starting from protected 2-aminothiol **S4** (225 mg, 0.50 mmol, 1.00 equiv.). HPLC purification and subsequent lyophilization from HCl afforded the compound as colorless oil (50.0 mg, 0.35 mmol, 70% for hydrochloride salt). $^1$H NMR (400 MHz, DMSO-$d_6$) $\delta$ = 2.71 (dd, *J* = 8.7, 6.4 Hz, 2H), 2.94 (t, *J* = 8.7 Hz, 1H), 3.08 – 3.18 (m, 1H), 3.57 – 3.68 (m, 2H), 8.15 (br s, 3H). $^{13}$C NMR (101 MHz, DMSO-$d_6$) $\delta$ = 22.8, 54.6, 59.1.

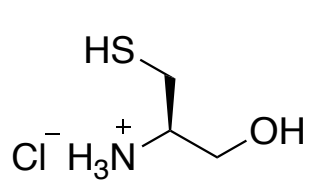

**(*R*)-2-Amino-*N*-benzyl-3-mercaptopropanamide hydrochloride (5).** The hydrochloride salt was prepared according to the general procedure (C) for TFA-mediated deprotection and hydrochloride salt formation starting from protected 2-aminothiol **S5** (275 mg, 0.50 mmol, 1.00 equiv.). HPLC purification and subsequent lyophilization from HCl afforded the compound as colorless solid (94.0 mg, 0.38 mmol, 76% for hydrochloride salt). HRMS *m/z*: $[M+H]^+$ Calcd for $C_{10}H_{15}N_2OS^+$: 211.0900; found: 211.0900. $^1$H NMR (400 MHz, DMSO-$d_6$) $\delta$ = 2.72 – 2.82 (m, 1H), 2.97 – 3.06 (m, 2H), 4.01 – 4.08 (m, 1H), 4.29 – 4.41 (m, 2H), 7.21 – 7.29 (m, 1H), 7.30 – 7.36 (m, 4H), 8.47 (br s, 3H), 9.11 – 9.20 (m, 1H). $^{13}$C NMR (101 MHz, DMSO-$d_6$) $\delta$ = 25.1, 42.4, 54.0, 127.0, 127.4 (2C), 128.3 (2C), 128.3, 138.5, 166.7.

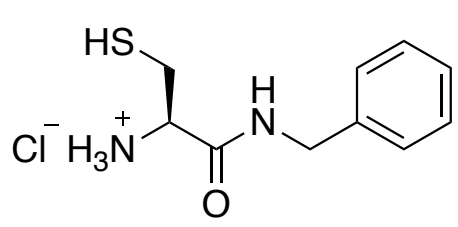

**(*R*)-2-Amino-3-mercapto-*N*-(2-(2-(2-methoxyethoxy)ethoxy)ethyl)propanamide hydrochloride (6).** The

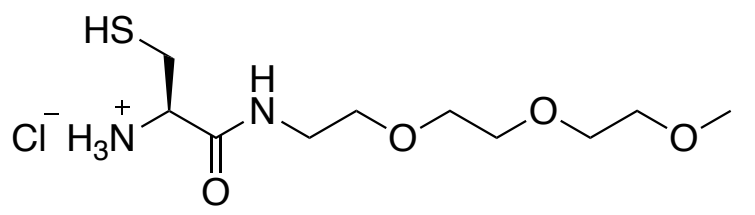

hydrochloride salt was prepared according to the general procedure (C) for TFA-mediated deprotection and hydrochloride salt formation starting from protected 2-aminothiol **S6** (304 mg, 0.50 mmol, 1.00 equiv.). HPLC purification and subsequent lyophilization from HCl afforded the compound as a colorless oil (121 mg, 0.40 mmol, 80% for hydrochloride salt). HRMS *m/z*: $[M+H]^+$ Calcd for $C_{10}H_{23}N_2O_4S^+$: 267.1373; found: 267.1372. $^1H$ NMR (400 MHz, DMSO-$d_6$) $\delta$ = 2.61 – 2.70 (m, 1H), 2.87 – 3.01 (m, 2H), 3.19 – 3.29 (m, 4H), 3.32 – 3.41 (m, 1H), 3.42 – 3.49 (m, 4H), 3.50 – 3.55 (m, 6H), 3.91 – 4.00 (m, 1H), 8.33 (br s, 3H), 8.56 – 8.65 (m, 1H).

**(*R*)-2-Amino-*N*-butyl-3-mercaptopropanamide hydrochloride (7).** The hydrochloride salt was prepared

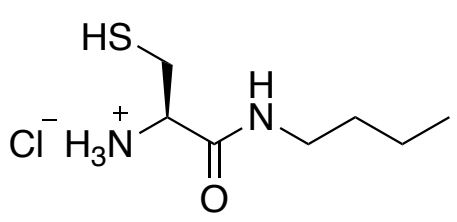

according to the general procedure (C) for TFA-mediated deprotection and hydrochloride salt formation starting from protected 2-aminothiol **S7** (259 mg, 0.50 mmol, 1.00 equiv.). HPLC purification and subsequent lyophilization from HCl afforded the compound as colorless oil (71.3 mg, 0.34 mmol, 69% for hydrochloride salt). HRMS *m/z*: $[M+H]^+$ Calcd for $C_7H_{17}N_2OS^+$: 177.1056; found: 177.1057. $^1H$ NMR (400 MHz, DMSO-$d_6$) $\delta$ = 0.87 (t, $J$ = 7.3 Hz, 3H), 1.27 – 1.35 (m, 2H), 1.39 – 1.46 (m, 2H), 2.69 – 2.76 (m, 1H), 2.89 – 3.01 (m, 2H), 3.03 – 3.12 (m, 1H), 3.12 – 3.21 (m, 1H), 3.89 – 3.96 (m, 1H), 8.38 (br s, 3H), 8.55 – 8.64 (m, 1H). $^{13}C$ NMR (101 MHz, DMSO-$d_6$) $\delta$ = 13.6, 19.5, 25.1, 30.9, 38.5, 53.9, 166.4.

**(*R*)-2-Amino-3-mercapto-1-(piperidin-1-yl)propan-1-one hydrochloride (8).** The hydrochloride salt was

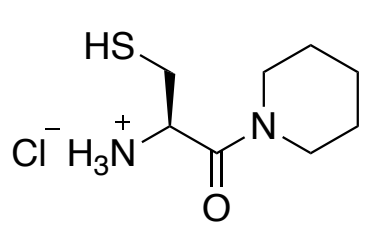

prepared according to the general procedure (C) for TFA-mediated deprotection and hydrochloride salt formation starting from protected 2-aminothiol **S8** (265 mg, 0.50 mmol, 1.00 equiv.). HPLC purification and subsequent lyophilization from HCl afforded the compound as colorless oil (77.1 mg, 0.34 mmol, 69% for hydrochloride salt). HRMS *m/z*: $[M+H]^+$ Calcd for $C_8H_{17}N_2OS^+$: 189.1056; found: 189.1057. $^1H$ NMR (400 MHz, DMSO-$d_6$) $\delta$ = 1.41 – 1.66 (m, 6H), 2.76 – 2.86 (m, 1H), 2.87 – 2.96 (m, 1H), 3.00 – 3.09 (m, 1H), 3.38 – 3.58 (m, 4H), 4.44 – 4.54 (m, 1H), 8.38 (br s, 1H). $^{13}C$ NMR (101 MHz, DMSO-$d_6$) $\delta$ = 23.8, 24.7, 25.2, 25.9, 42.8, 46.0, 51.2, 165.0.

**(*R*)-2-Amino-3-mercapto-*N*-phenylpropanamide hydrochloride (9).** The hydrochloride salt was prepared

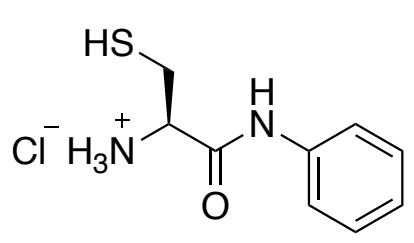

according to the general procedure (C) for TFA-mediated deprotection and hydrochloride salt formation starting from protected 2-amino thiol **S9** (269 mg, 0.50 mmol, 1.00 equiv.). HPLC purification and subsequent lyophilization from HCl afforded the compound as white solid (65.8 mg, 0.28 mmol, 56% for hydrochloride salt). HRMS *m/z*: $[M+H]^+$ Calcd for $C_9H_{13}N_2OS^+$: 197.0743; found: 197.0746. $^1H$ NMR (400 MHz, DMSO-$d_6$) $\delta$ = 2.90 (t, $J$ = 8.5 Hz, 1H), 3.03 – 3.15 (m, 2H), 4.20 – 4.28 (m, 1H), 7.11 (t, $J$ = 7.4 Hz, 1H), 7.35 (t, $J$ = 7.9 Hz, 2H), 7.66 (d, $J$ = 7.9 Hz, 2H), 8.54 (br s, 3H), 10.93 (s, 1H). $^{13}C$ NMR (101 MHz, DMSO-$d_6$) $\delta$ = 25.1, 54.7, 119.5 (2C), 124.0, 128.8 (2C), 138.2, 165.5. Data are in accordance with previous report.[4]

**(*R*)-4-(2-Amino-3-mercaptopropanamido)benzoic acid hydrochloride (10).** The hydrochloride salt was

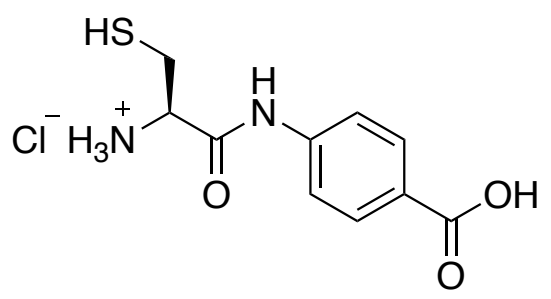

prepared according to the general procedure (C) for TFA-mediated deprotection and hydrochloride salt formation starting from protected 2-aminothiol **S10** (319 mg, 0.50 mmol, 1.00 equiv.). HPLC purification and subsequent lyophilization from HCl afforded the compound as white solid (91.3 mg, 0.33 mmol, 66% for hydrochloride salt). HRMS *m/z*: $[M+H]^+$ Calcd for $C_{10}H_{13}N_2O_3S^+$: 241.0641; found: 241.0643. $^1$H NMR (400 MHz, DMSO-$d_6$) $\delta$ = 2.87 – 2.99 (m, 1H), 3.03 – 3.19 (m, 2H), 4.26 – 4.34 (m, 1H), 7.78 (d, $J$ = 8.8 Hz, 2H), 7.93 (d, $J$ = 8.8 Hz, 2H), 8.56 (br s, 3H), 11.30 (s, 1H), 12.78 (s, 1H). $^{13}$C NMR (101 MHz, DMSO-$d_6$) $\delta$ = 25.0, 54.8, 118.9 (2C), 126.0, 130.4 (2C), 142.1, 166.0, 166.8.

**(*R*)-2-Amino-3-mercapto-*N*-(3,4,5-trimethoxyphenyl)propanamide hydrochloride (11).** The hydrochloride salt

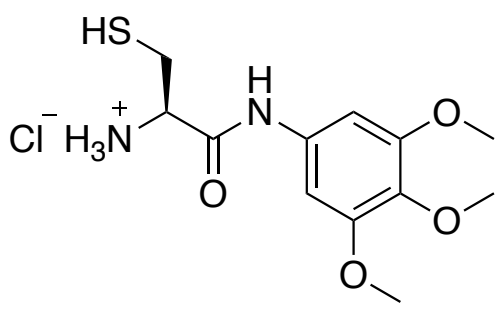

was prepared according to the general procedure (C) for TFA-mediated deprotection and hydrochloride salt formation starting from protected 2-aminothiol **S11** (314 mg, 0.50 mmol, 1.00 equiv.). HPLC purification and subsequent lyophilization from HCl afforded the compound as white solid (114 mg, 0.35 mmol, 70% for hydrochloride salt). HRMS *m/z*: $[M+H]^+$ Calcd for $C_{12}H_{19}N_2O_4S^+$: 287.1060; found: 287.1063. $^1$H NMR (400 MHz, DMSO-$d_6$) $\delta$ = 2.83 – 2.96 (m, 1H), 3.03 – 3.13 (m, 2H), 3.63 (s, 3H), 3.75 (s, 6H), 4.18 (t, $J$ = 5.7 Hz, 1H), 7.05 (s, 2H), 8.52 (br s, 3H), 10.83 (s, 1H). $^{13}$C NMR (101 MHz, DMSO-$d_6$) $\delta$ = 25.1, 54.8, 55.7 (2C), 60.1, 97.3 (2C), 134.0, 134.2, 152.7 (2C), 165.2.

**(*R*)-2-Amino-3-mercaptopropanamide hydrochloride (12).** The hydrochloride salt was prepared according to the

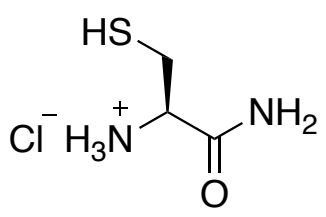

general procedure (C) for TFA-mediated deprotection and hydrochloride salt formation starting from protected 2-aminothiol **S12** (231 mg, 0.50 mmol, 1.00 equiv.). HPLC purification and subsequent lyophilization from HCl afforded the compound as white solid (43.1 mg, 0.28 mmol, 56% for hydrochloride salt). HRMS *m/z*: $[M+H]^+$ Calcd for $C_3H_9N_2OS^+$: 121.0430; found: 121.0433. $^1$H NMR (400 MHz, DMSO-$d_6$) $\delta$ = 2.72 – 2.87 (m, 1H), 3.00 (d, $J$ = 5.3 Hz, 2H), 3.95 (t, $J$ = 5.3 Hz, 1H), 7.61 (s, 1H), 8.00 (s, 1H), 8.36 (br s, 3H). $^{13}$C NMR (101 MHz, DMSO-$d_6$) $\delta$ = 25.0, 53.9, 168.4.

## Synthesis of peptide S13

The linear AIP-I peptide, containing an additional C-terminal cysteine residue (**S13**), was synthesized in 40 µmol scale on Fmoc-Cys(Trt)-loaded TentaGel® S PHB resin (Rapp Polymere). Synthesis was carried out on automated peptide synthesizer (Biotage SyroWave™) using iterative coupling and deprotection steps. The coupling of the first amino acid was performed for 90 min at room temperature with short vortexing intervals (10 s) using Fmoc-Met-OH in DMF (5.00 equiv to the resin loading, 0.5 M), HATU in DMF (4.90 equiv, 0.5 M) and *i*-$Pr_2$NEt in NMP (10.0 equiv, 2.0 M) at a final concentration of 0.2 M for Fmoc-Met-OH. The coupling was followed by washing of the resin with DMF (5 × 1 min) and the procedure was repeated to achieve a double coupling. Subsequent coupling steps were performed for 40 min at room temperature with short vortexing intervals (10 s) using stock solutions of Fmoc-AA-OH (Fmoc-Tyr(tBu)-OH, Fmoc-Ser(tBu)-OH, Fmoc-Thr(tBu)-OH, Fmoc-Cys(Trt)-OH, Fmoc-Asp(tBu)-OH, Fmoc-Phe-OH, Fmoc-Ile-OH) in DMF (5.00 equiv to the resin loading, 0.5 M), HBTU in DMF (4.90 equiv, 0.5 M) and *i*-$Pr_2$NEt in NMP (10.0 equiv, 2.0 M) at a final concentration of 0.2 M for Fmoc-AA-OH.

The coupling reaction was followed by washing of the resin with DMF (5 × 1 min) and the procedure was repeated to achieve a double coupling.

Fmoc removal was performed in two stages: 1) piperidine in DMF (2:3, v/v) for 3 min and 2) piperidine in DMF (1:4, v/v) for 12 min with short vortexing intervals (10 s). The deprotection was followed by washing of the resin with DMF (6 × 1 min).

Following peptide elongation, the resin was transferred to a polypropylene syringe equipped with a fritted disk and washed with DMF (3 × 1 min), MeOH (3 × 1 min), and $CH_2Cl_2$ (3 × 1 min) and dried under suction for 10 min. The dried resin was treated with 4 mL of a cleavage solution (TFA–$H_2O$–*i*-$Pr_2$Net, 95:2.5:2.5, v/v) for 2 h at room temperature to obtain the fully deprotected peptide (**S13**) from the resin. The cleavage solution was collected and the resin rinsed with neat TFA (1 mL) which was combined with the cleavage solution. The solution was evaporated to near dryness under a stream of $N_2$ and the crude peptide triturated with ice-cold $Et_2O$ (20 mL) and centrifuged for 5 min (1000 rpm). The $Et_2O$ phase was decanted and the process repeated once more. Subsequently, the remaining residue was dissolved in ($H_2O$–MeCN, 1:1, v/v) and purified by preparative HPLC to afford the pure linear peptide after lyophilization.

## Supporting references


[1] Y. X. Tan, D. S. Peters, S. I. Walsh, M. Holcomb, D. Santos-Martins, S. Forli, F. E. Romesberg, *J Nat Prod* **2020**, *83*, 2112-2121.

[2] M. E. Jung, S. W. Yi, *Tetrahedron Lett* **2012**, *53*, 4216-4220.

[3] Y. Kato, T. Kuroda, Y. Huang, R. Ohta, Y. Goto, H. Suga, *Angew Chem Int Ed Engl* **2020**, *59*, 684-688.

[4] K. Li, W. Wang, J. Gao, *Angew Chem Int Ed Engl* **2020**, *59*, 14246-14250.

[5] C. L. Malone, B. R. Boles, K. J. Lauderdale, M. Thoendel, J. S. Kavanaugh, A. R. Horswill, *J Microbiol Methods* **2009**, *77*, 251-260.

[6] L. I. Kupferwasser, M. R. Yeaman, C. C. Nast, D. Kupferwasser, Y. Q. Xiong, M. Palma, A. L. Cheung, A. S. Bayer, *J Clin Invest* **2003**, *112*, 222-233.

[7] a) C. Vingsbo Lundberg, N. Frimodt-Moller, *Int J Antimicrob Agents* **2013**, *42*, 272-275; b) B. H. Gless, B. S. Sereika-Bejder, I. Jensen, M. S. Bojer, K. Tsiko, S. H. Schmied, L. Vitolo, B. Toledo-Silva, S. De Vliegher, H. Ingmer, C. A. Olsen, *mBio* **2025**, *16*, e0096725.

[8] H. Y. Shiu, M. K. Wong, C. M. Che, *Chem Commun* **2011**, *47*, 4367-4369.

## Copies of NMR spectra of final compounds

**Benzyl *L*-cysteinate hydrochloride (1)**

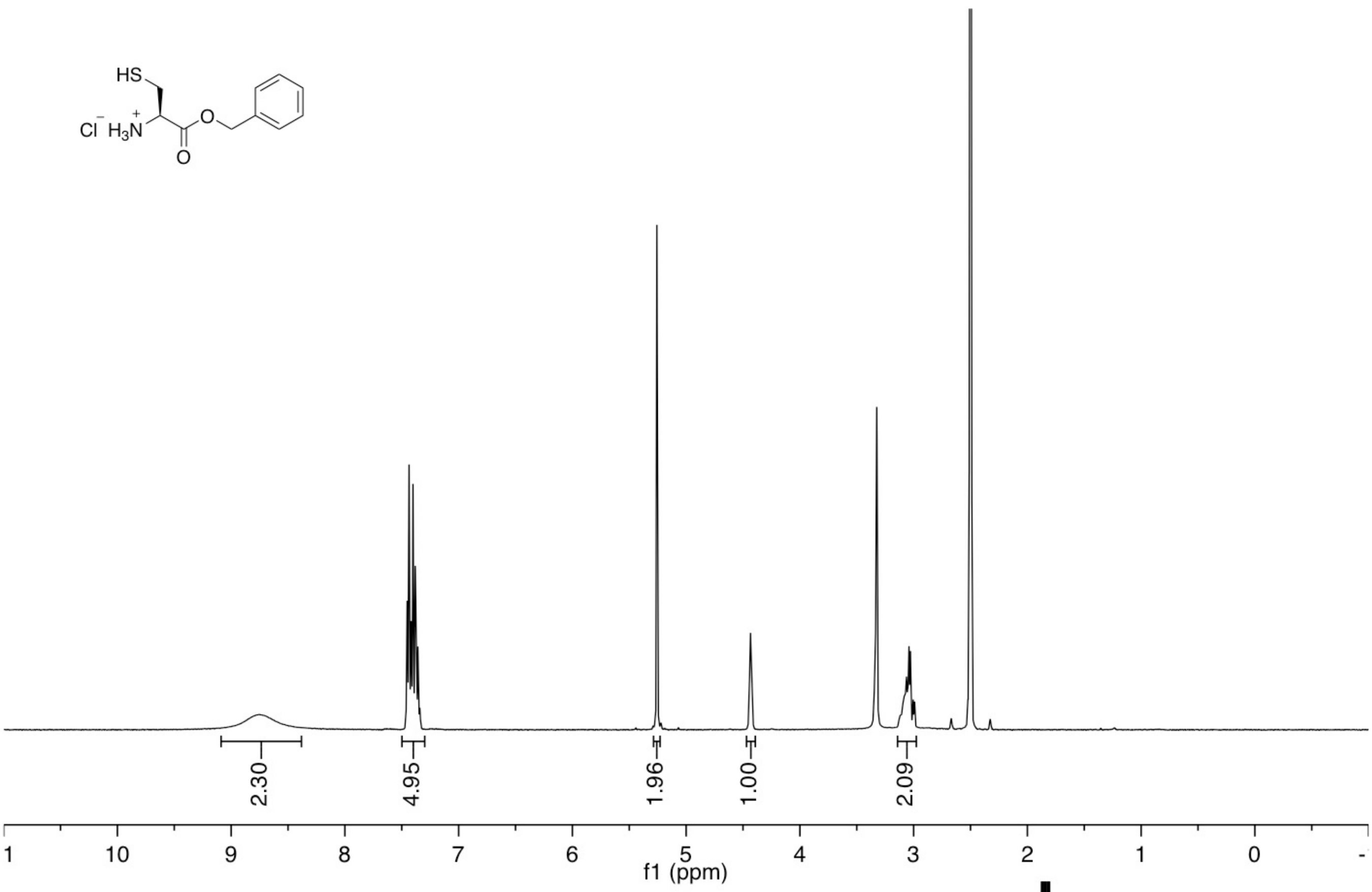


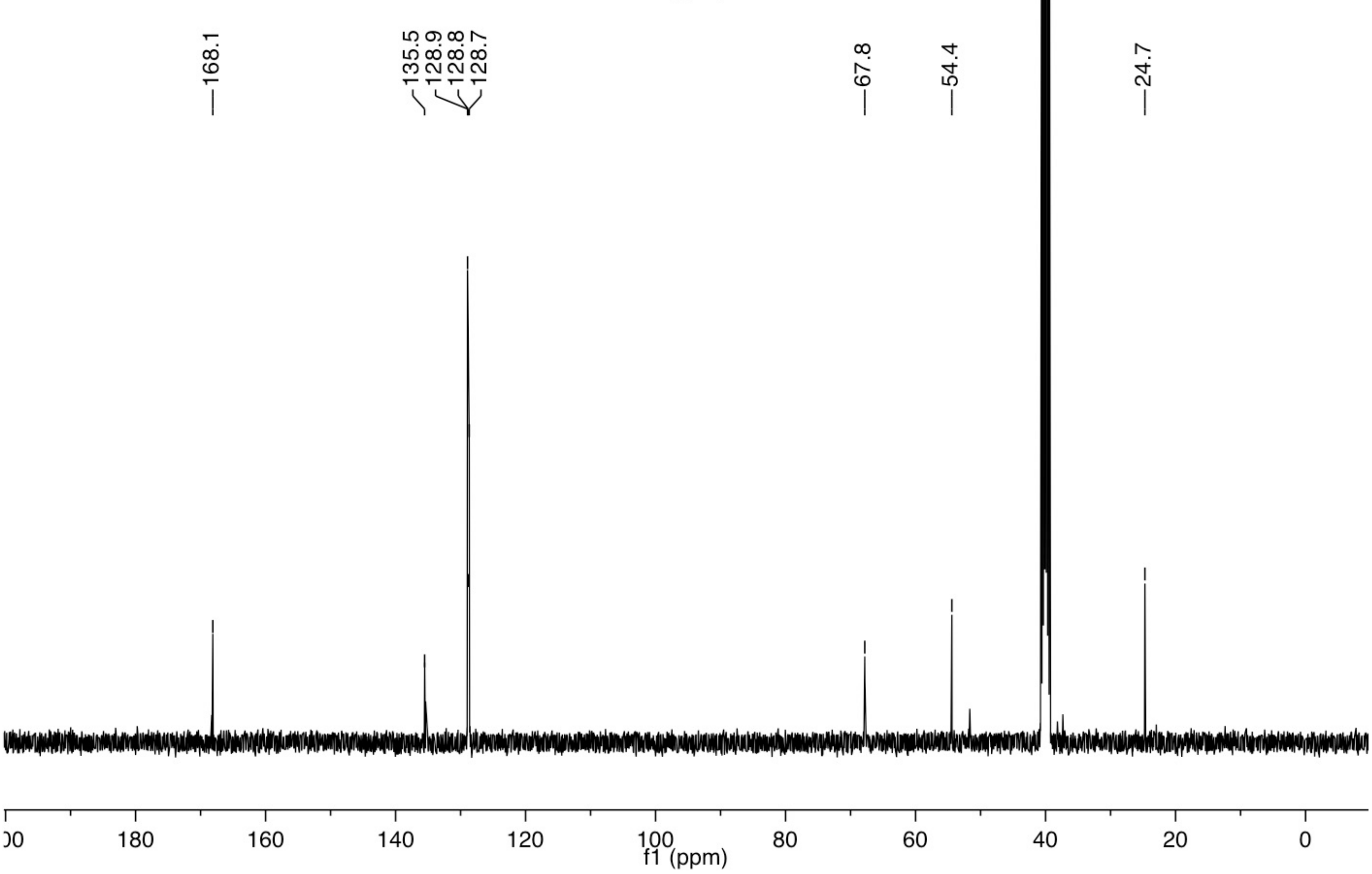

**2-Amino-2-oxoethyl *L*-cysteinate hydrochloride (2)**

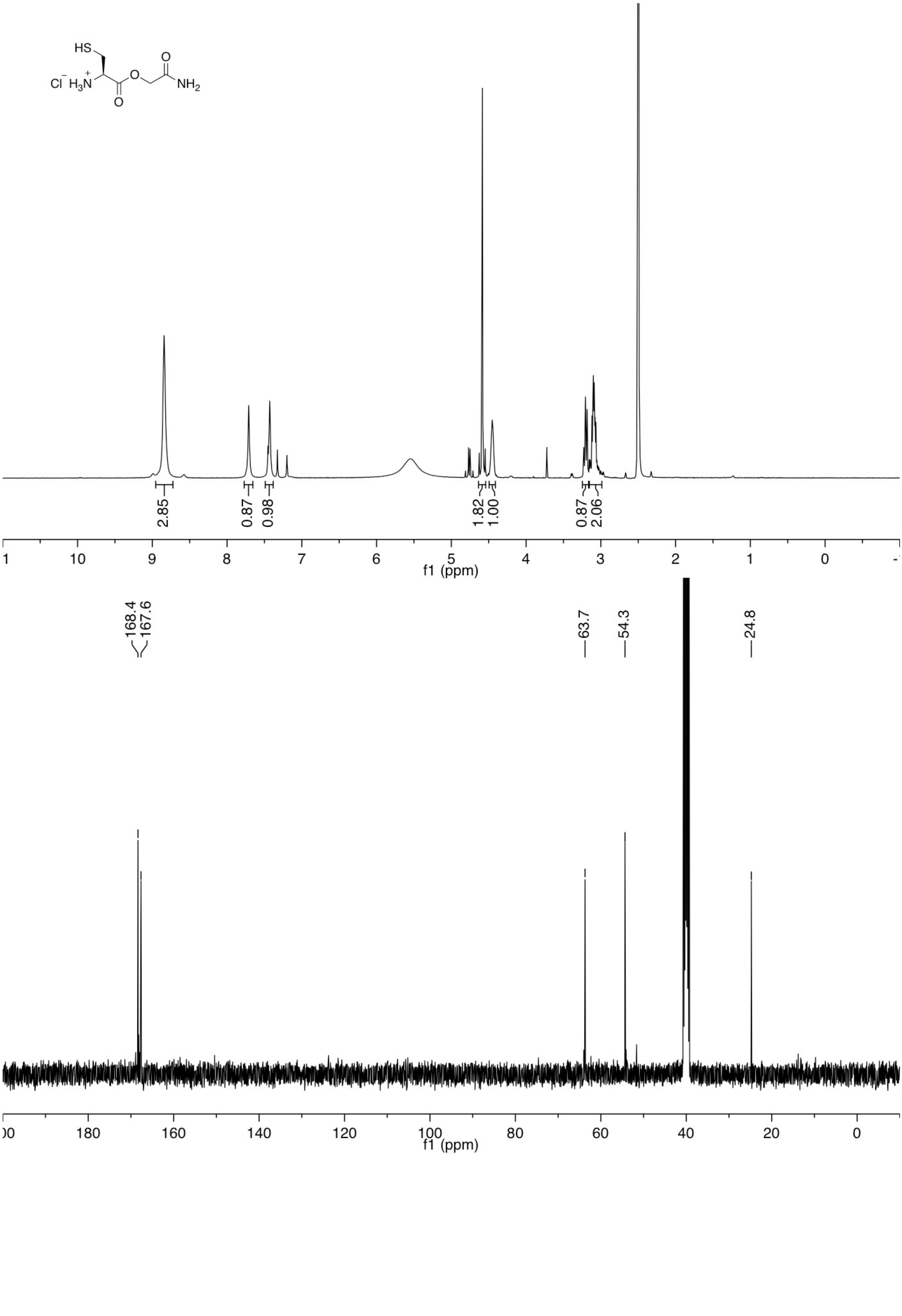

**Cyclohexyl *L*-cysteinate hydrochloride (3)**

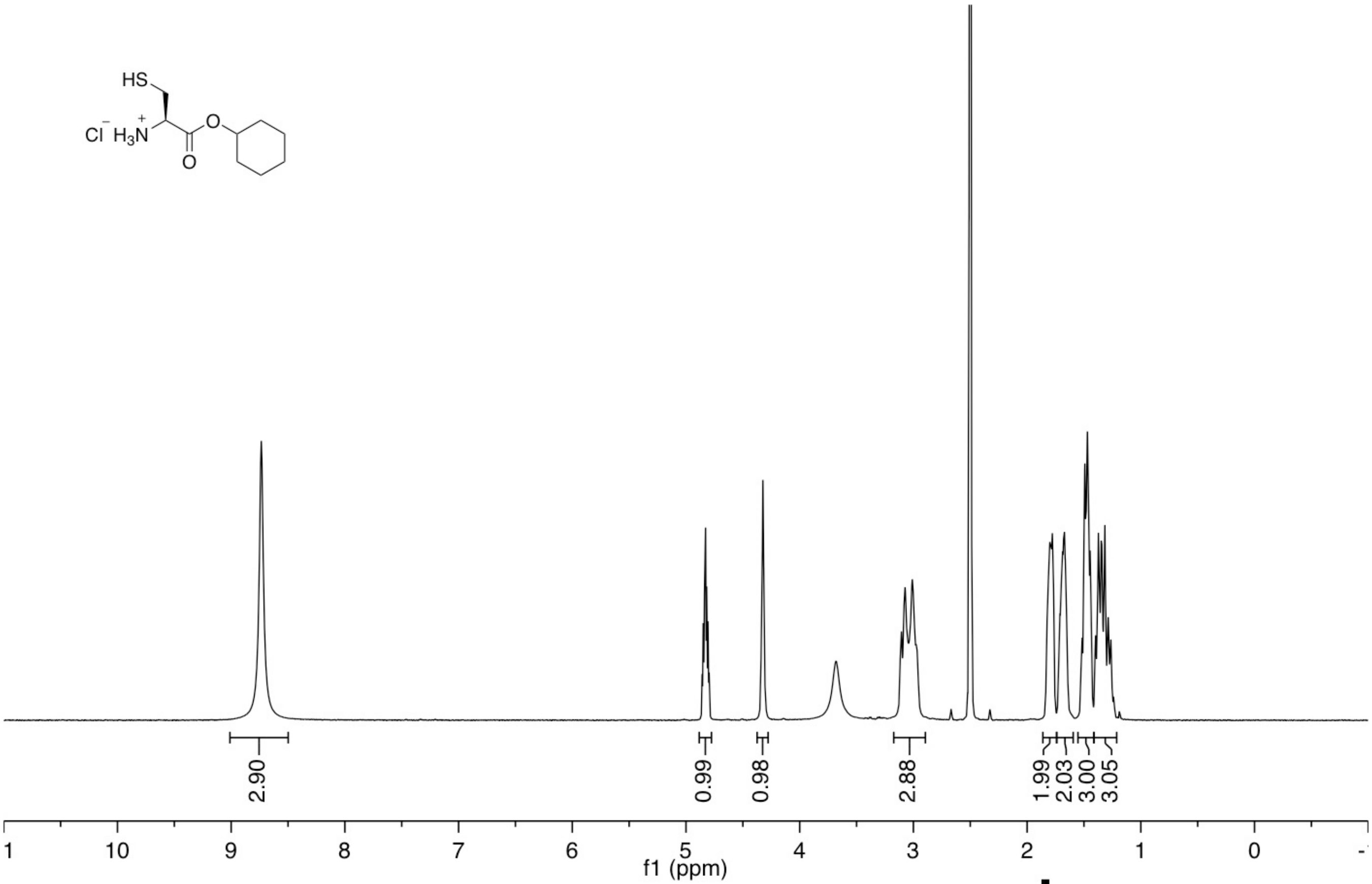


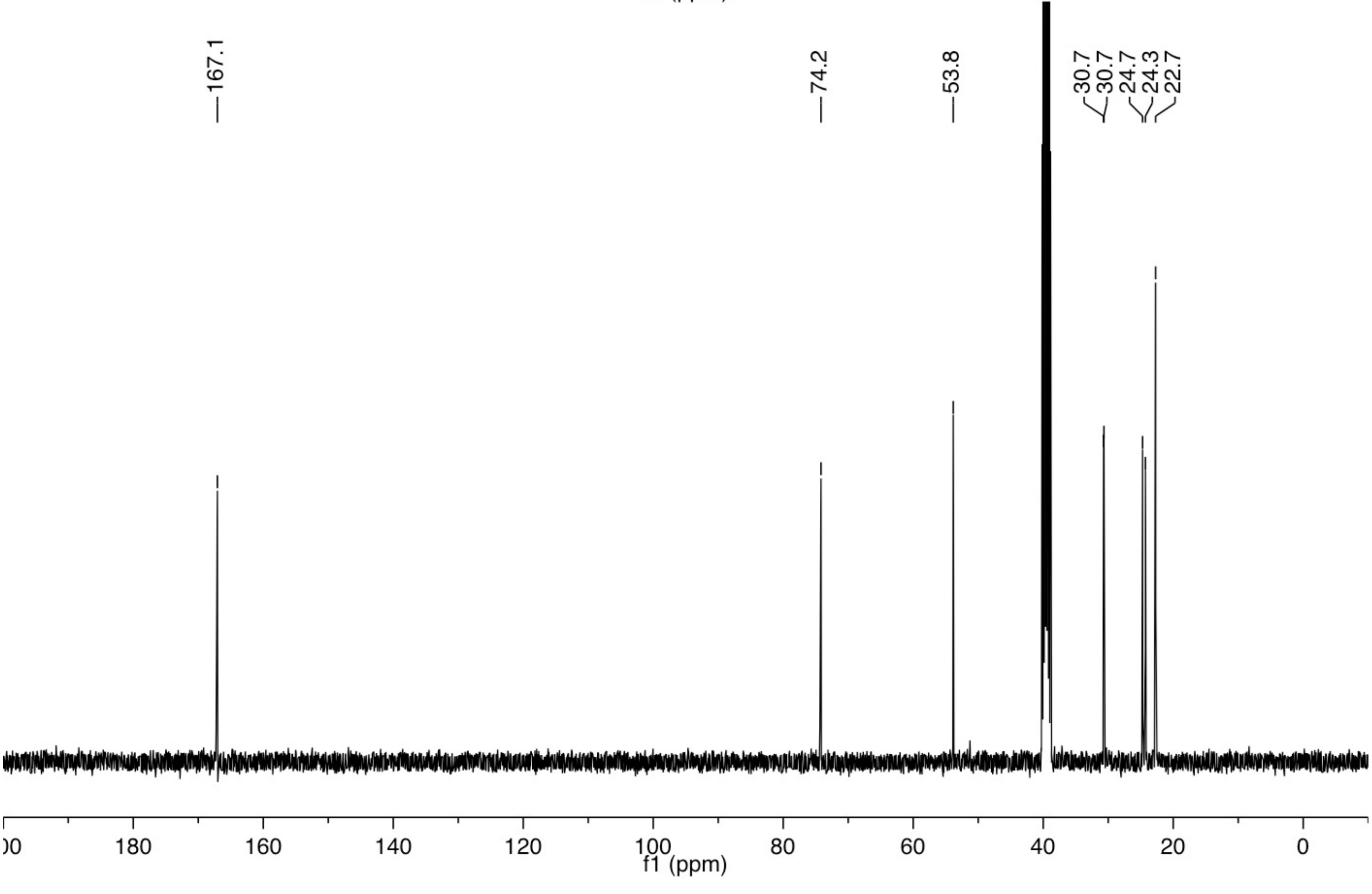

**(*R*)-2-Amino-3-mercaptopropan-1-ol hydrochloride (4)**

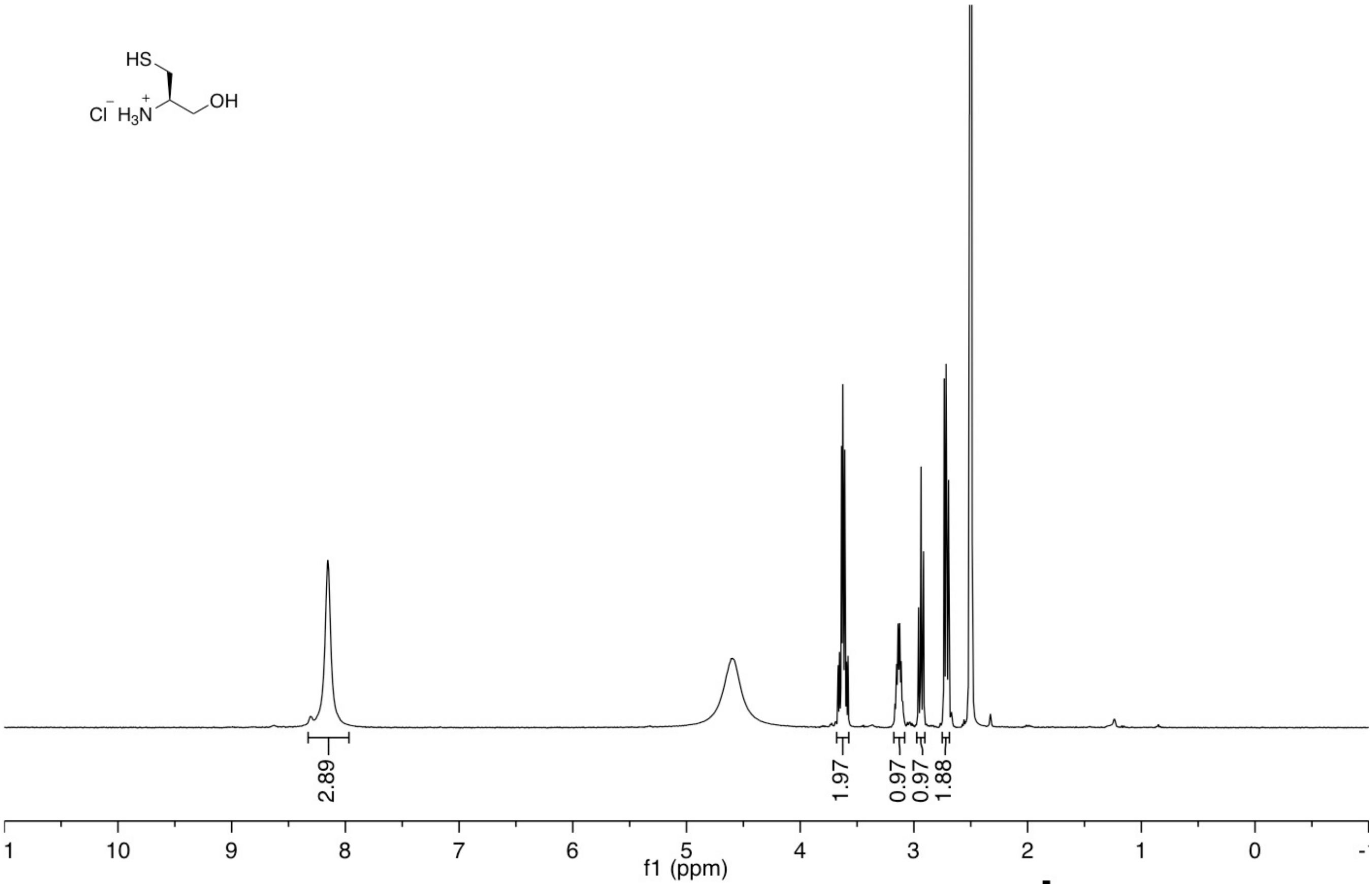


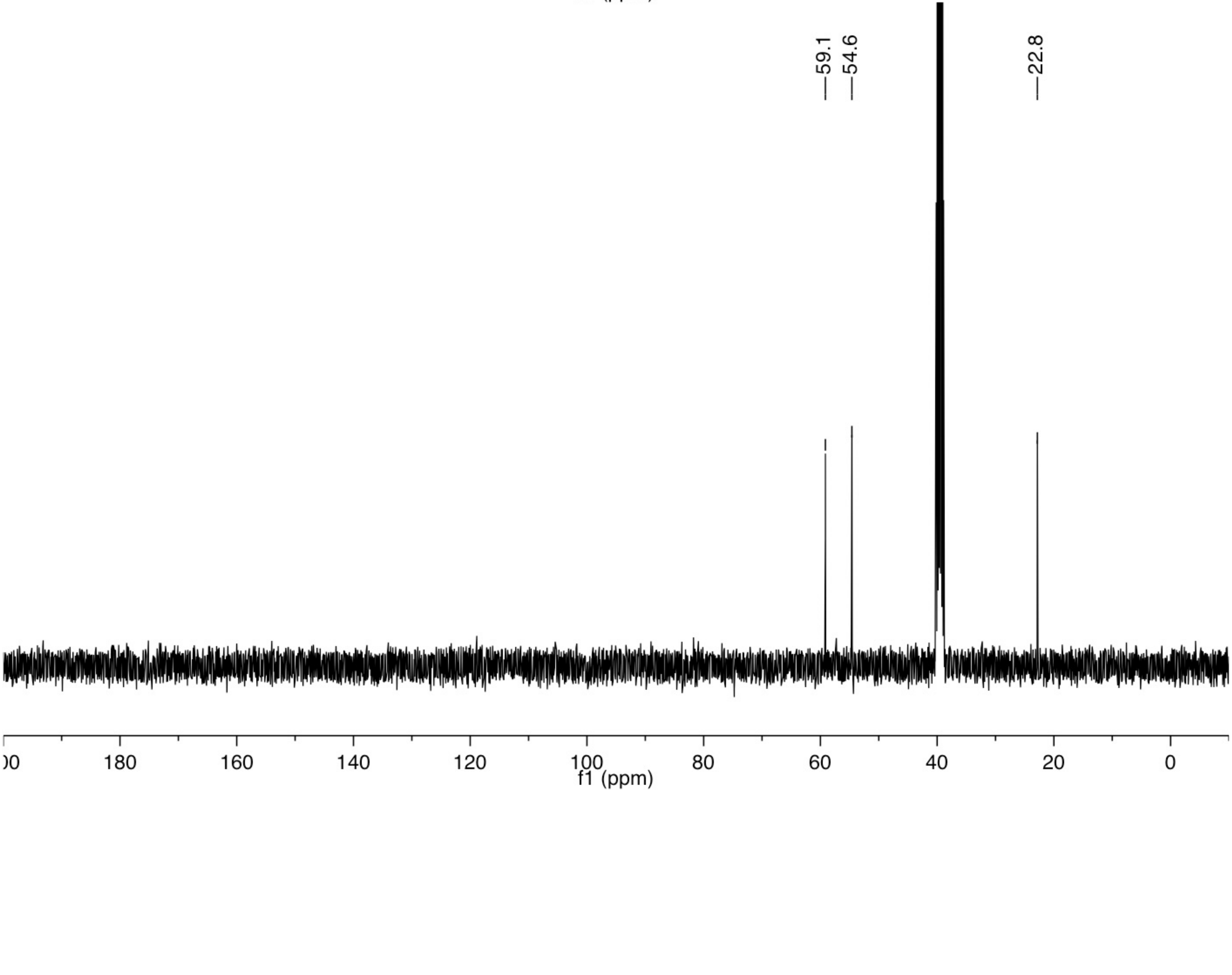

**(*R*)-2-Amino-*N*-benzyl-3-mercaptopropanamide hydrochloride (5)**

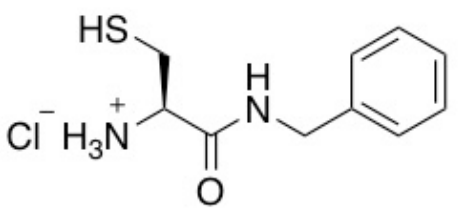


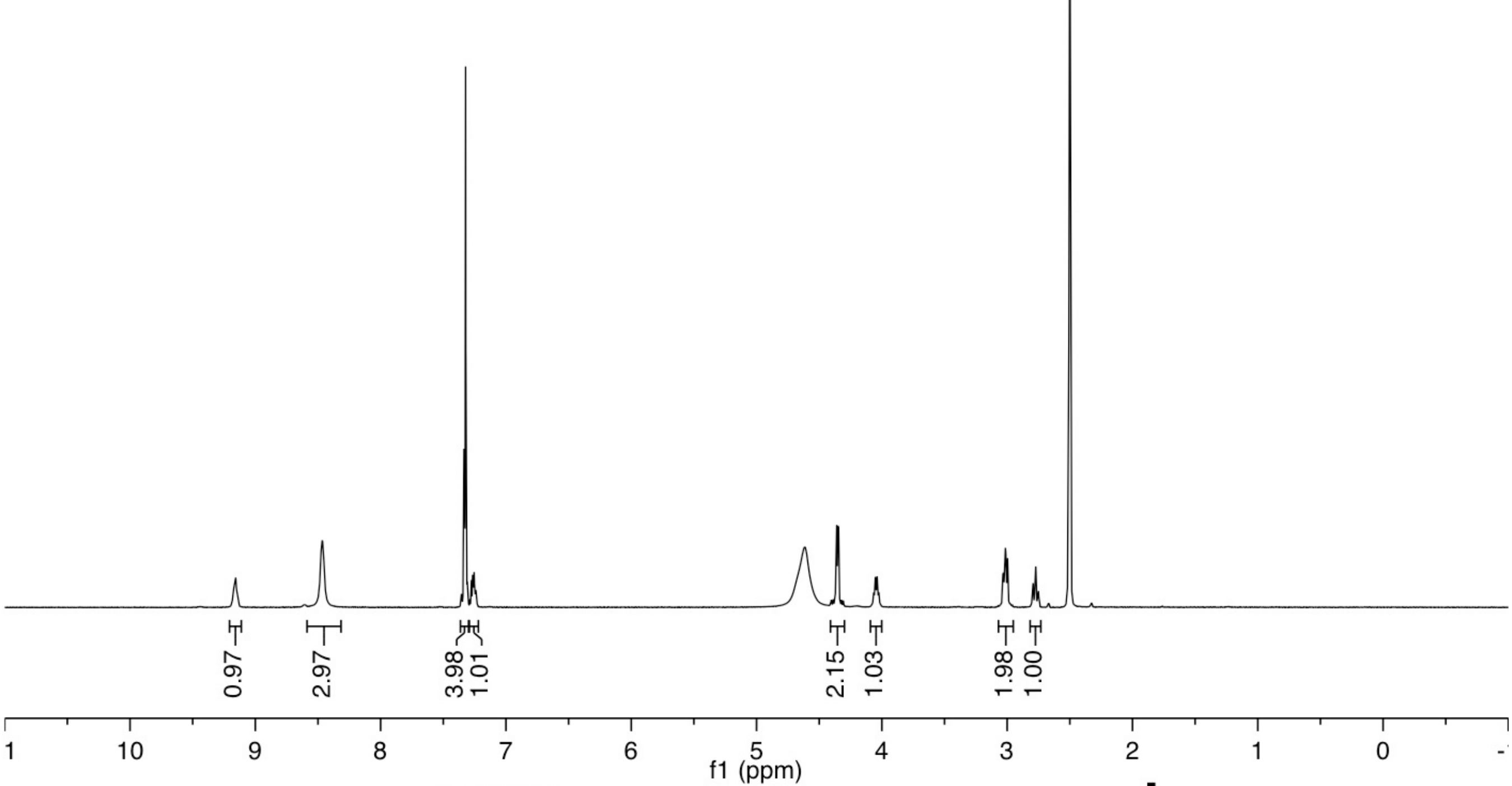


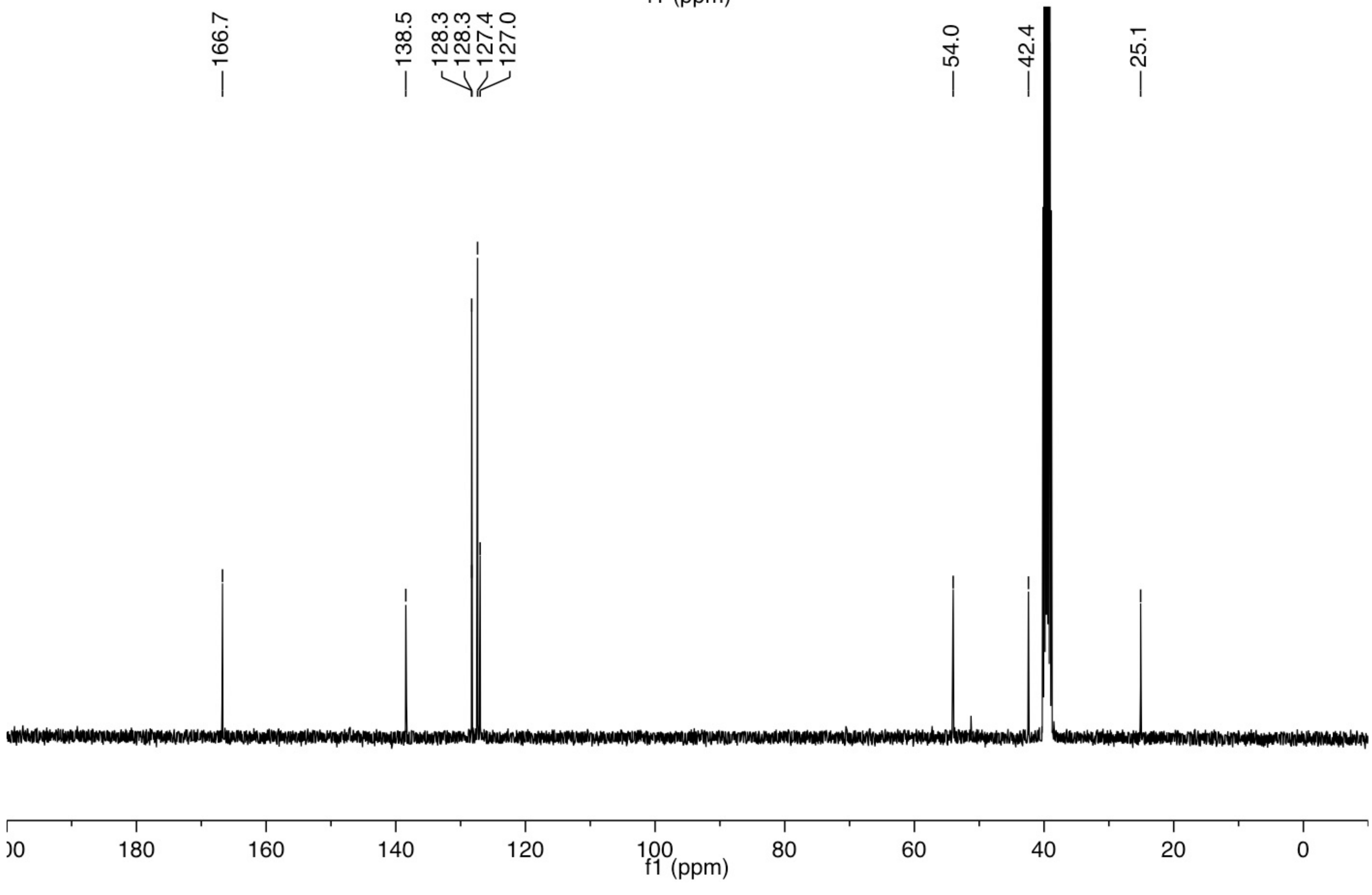

**(*R*)-2-Amino-3-mercapto-*N*-(2-(2-(2-methoxyethoxy)ethoxy)ethyl)propanamide hydrochloride (6)**

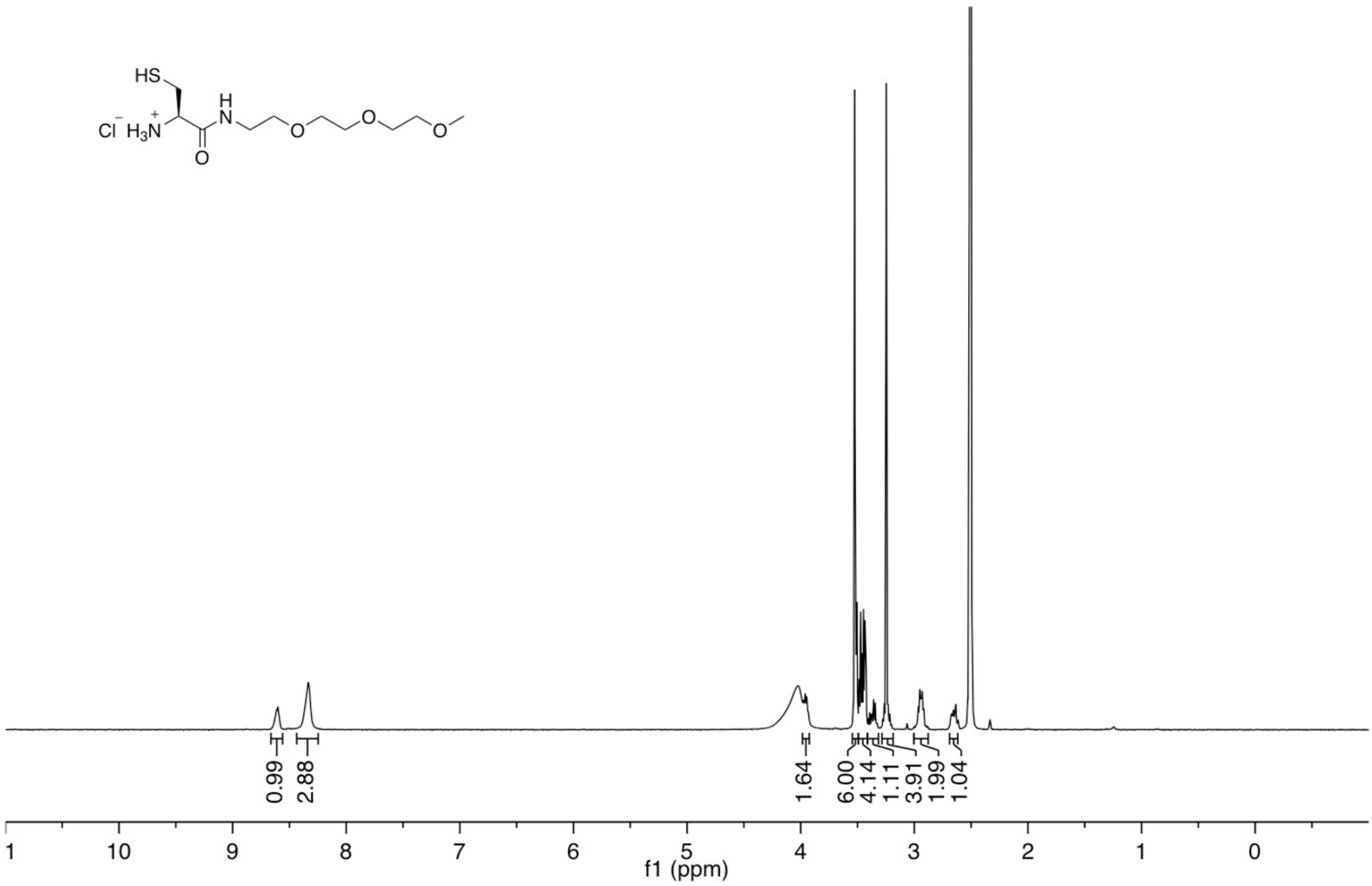


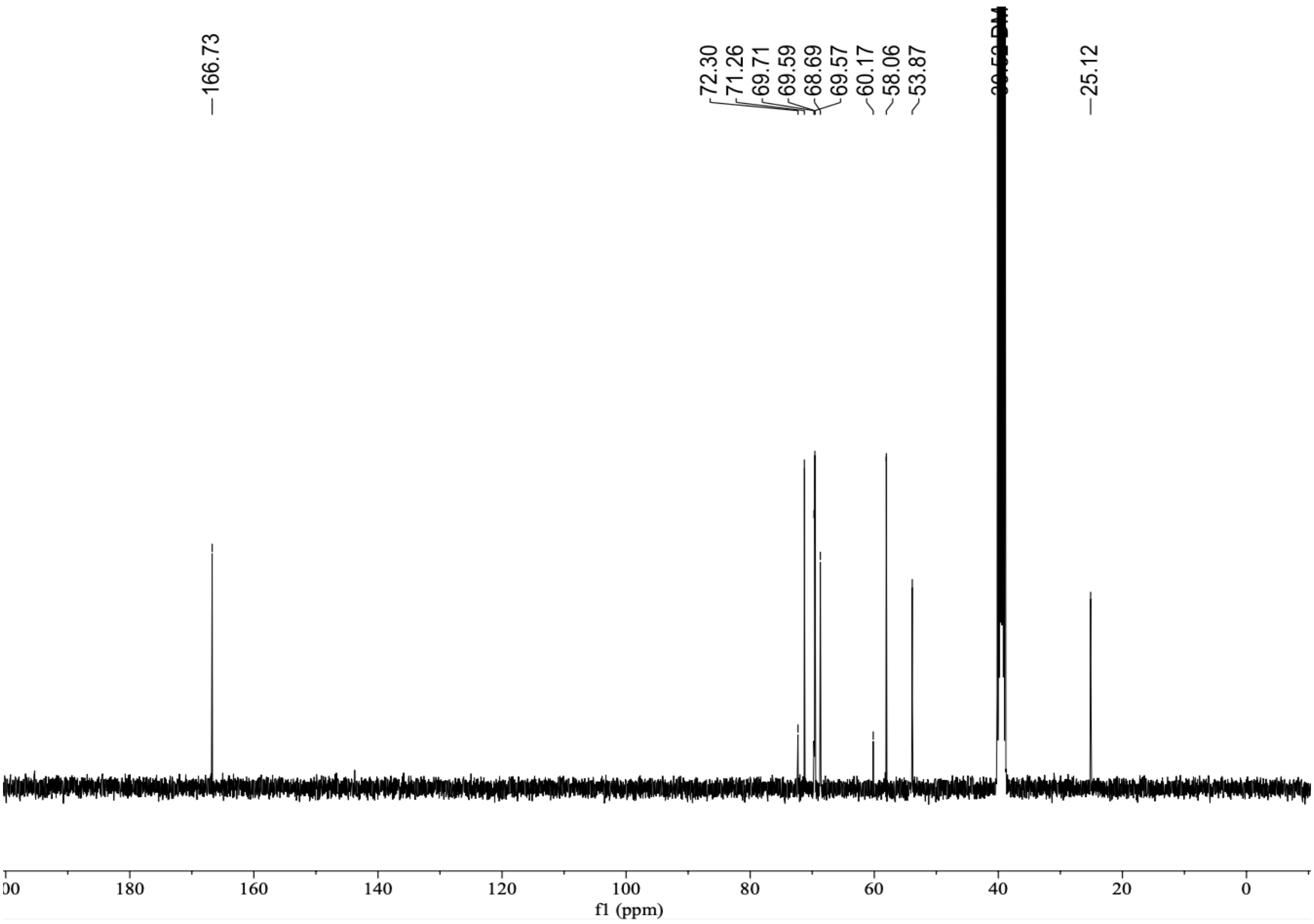

**(*R*)-2-Amino-*N*-butyl-3-mercaptopropanamide hydrochloride (7)**

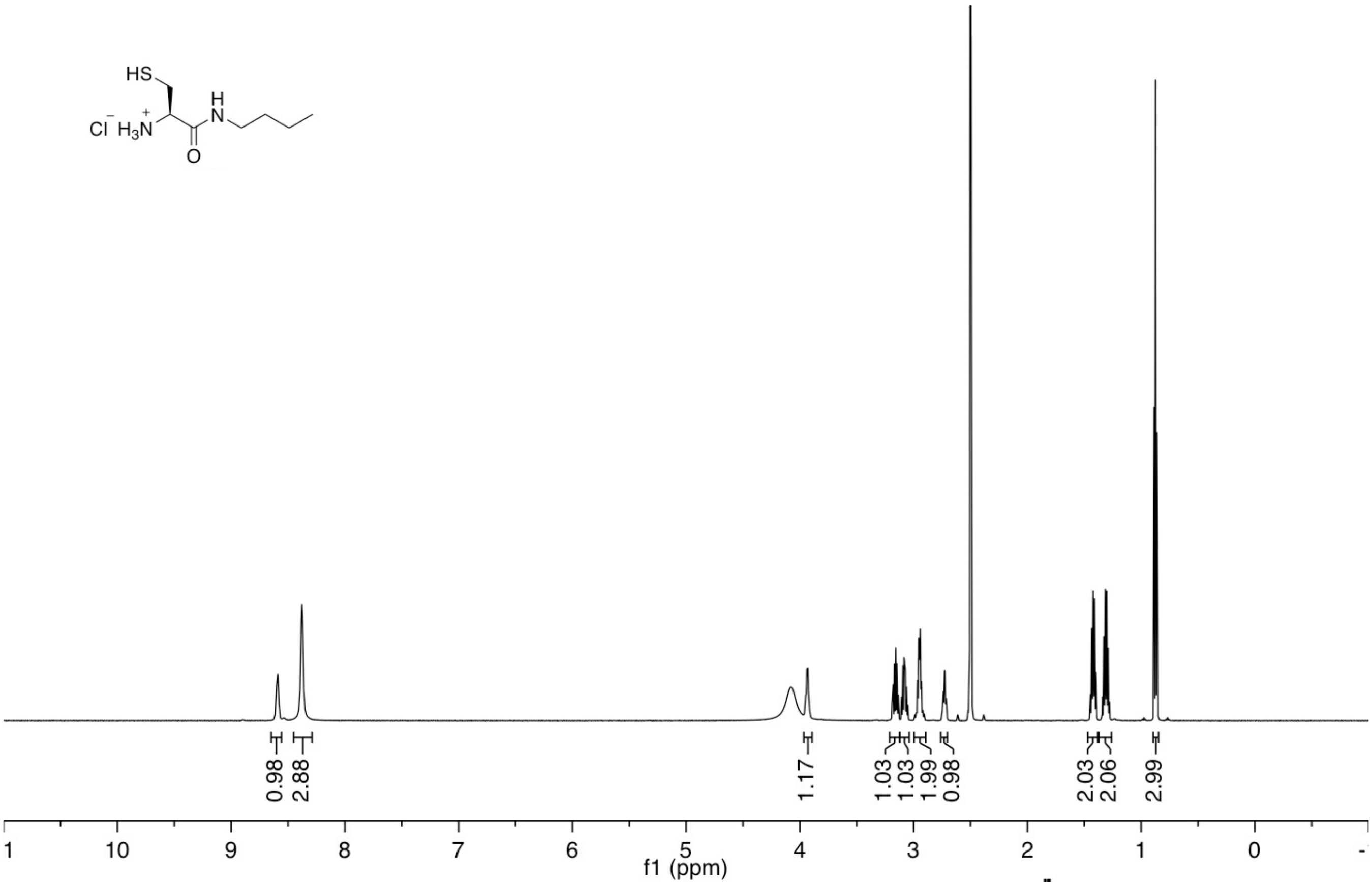


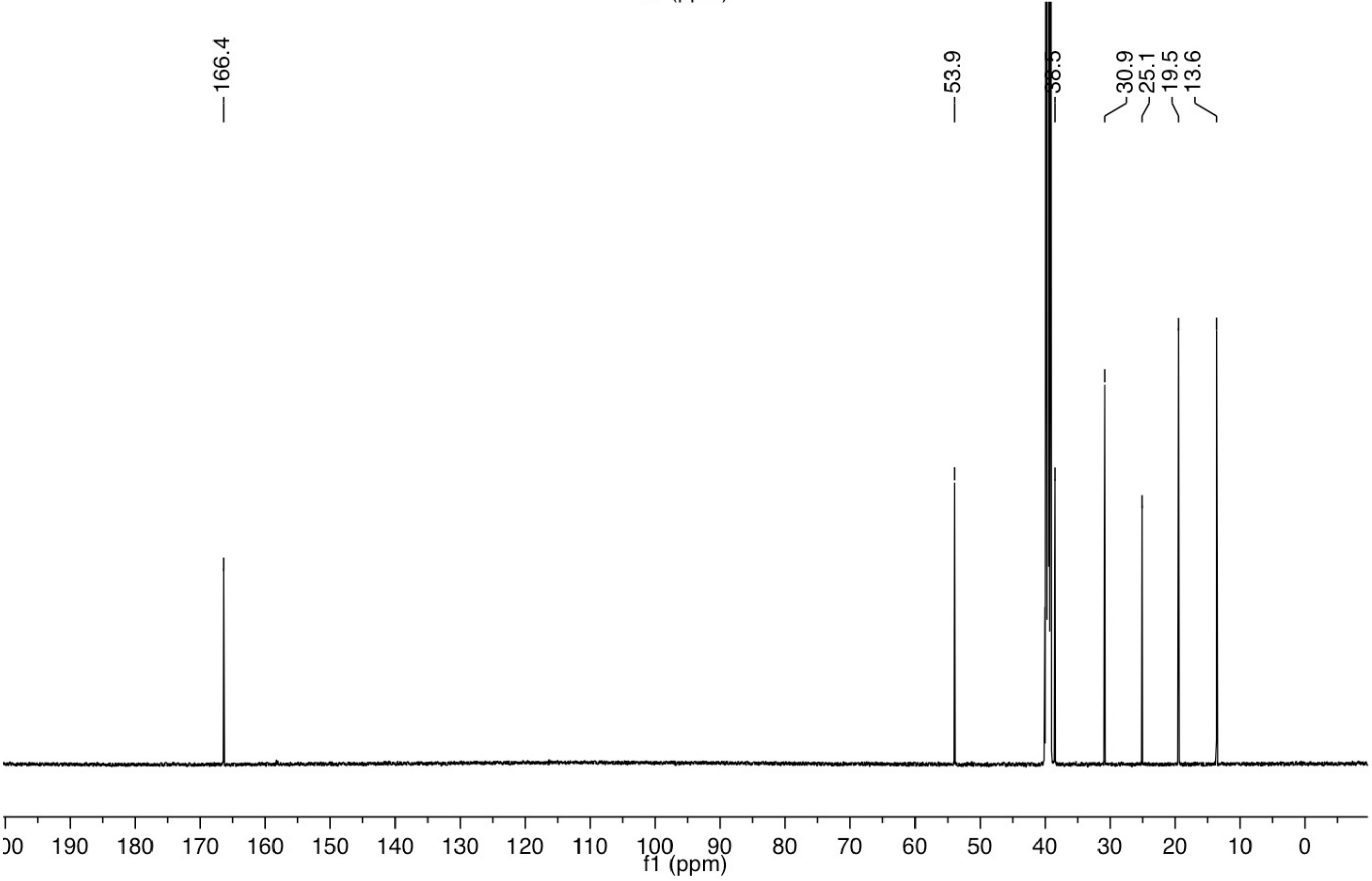

**(*R*)-2-Amino-3-mercapto-1-(piperidin-1-yl)propan-1-one hydrochloride (8)**

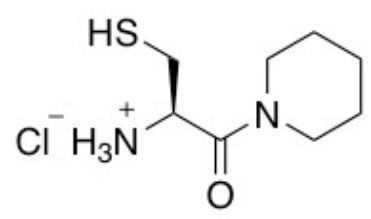


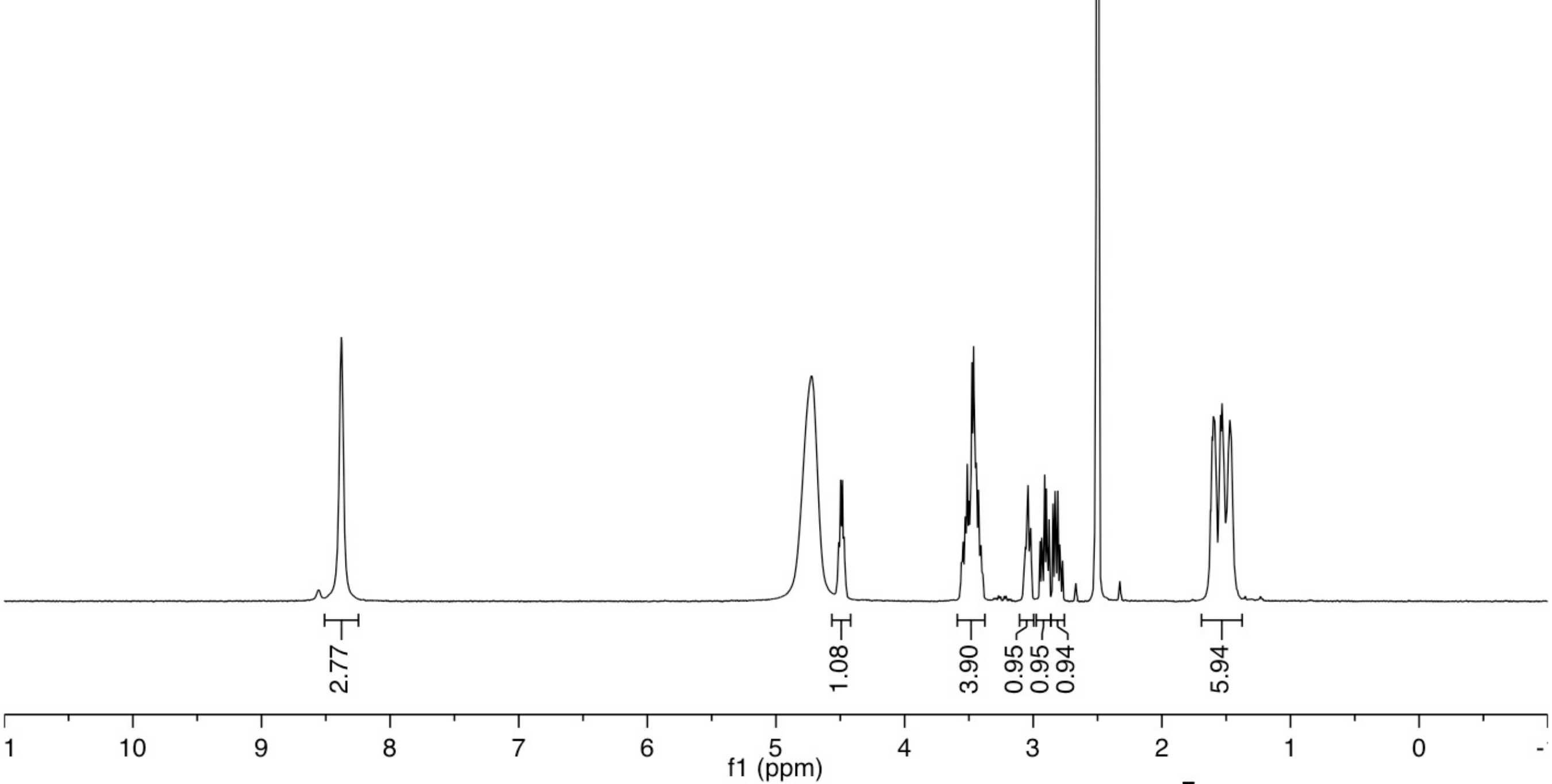


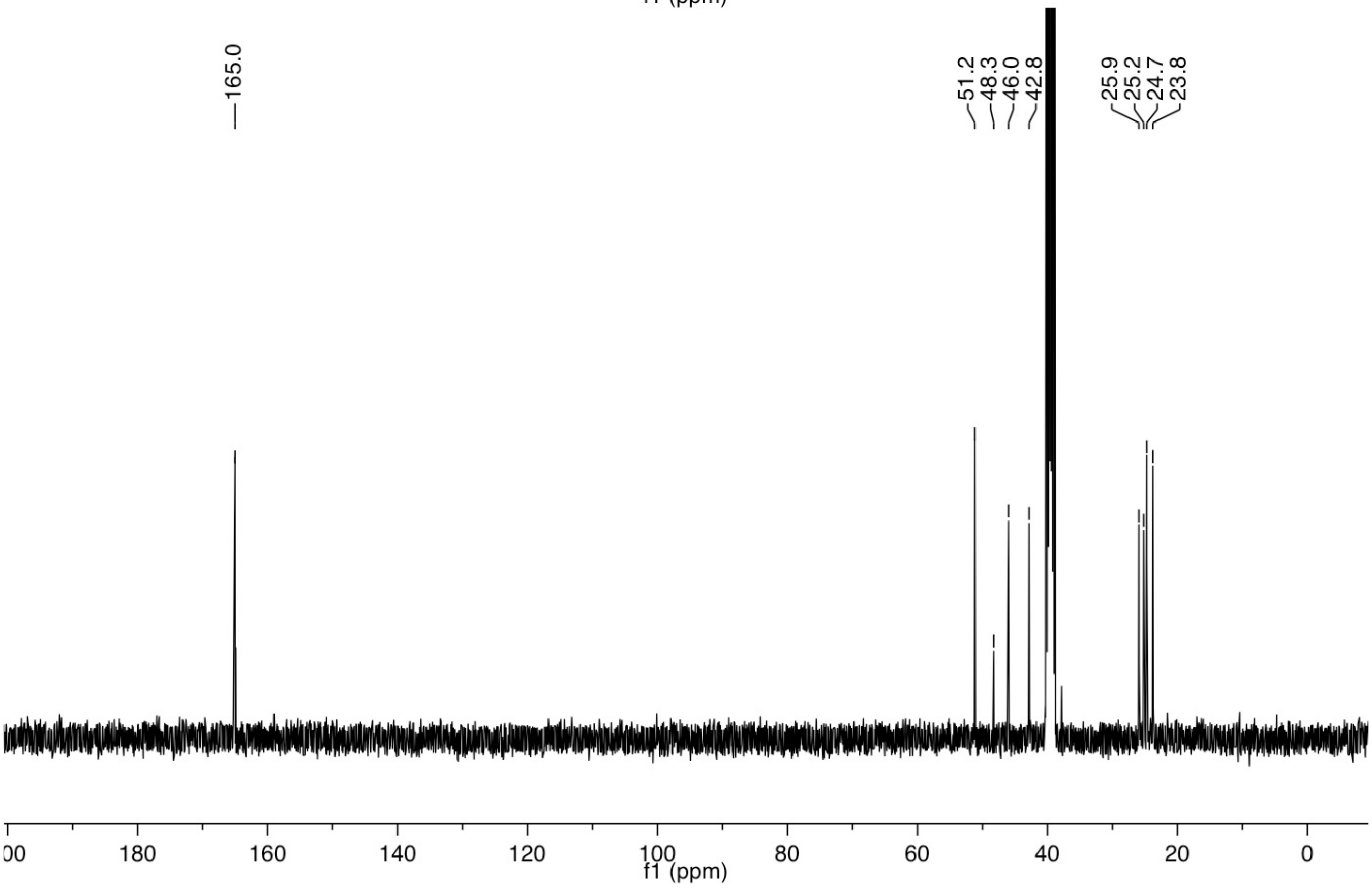

**(*R*)-2-Amino-3-mercapto-*N*-phenylpropanamide hydrochloride (9)**

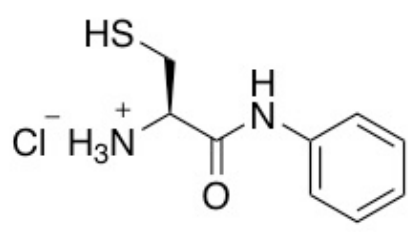


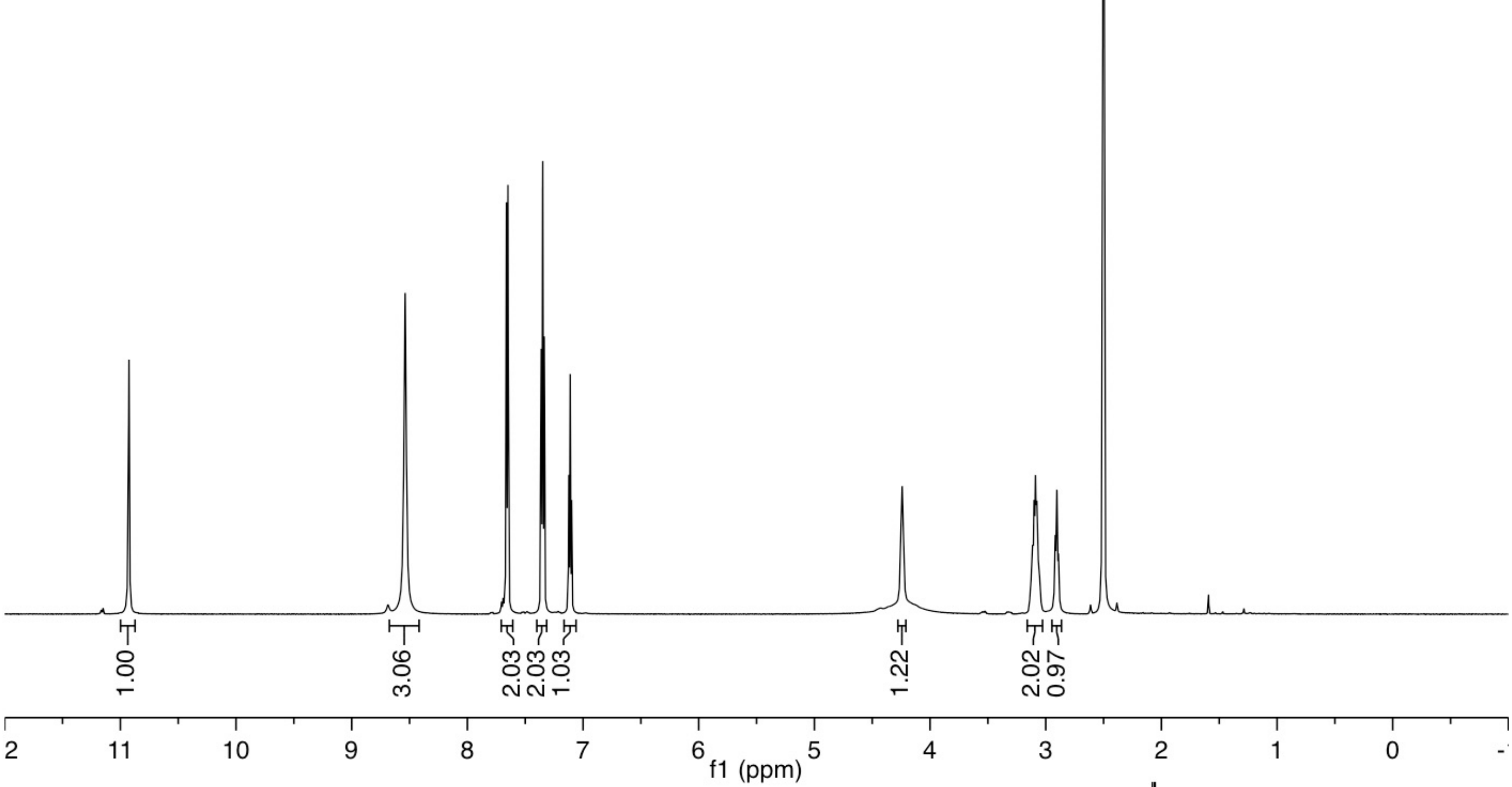


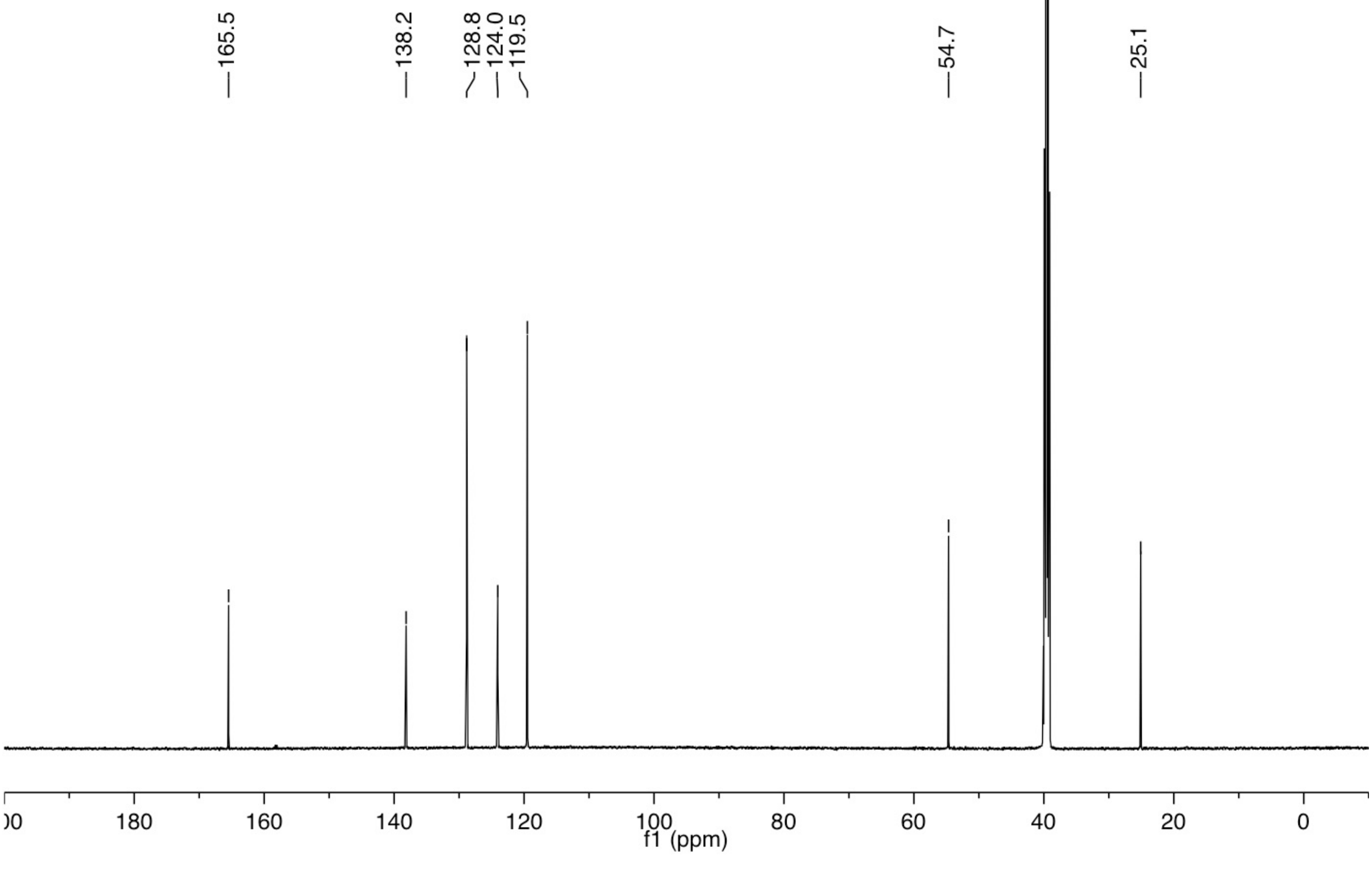

**(*R*)-4-(2-Amino-3-mercaptopropanamido)benzoic acid hydrochloride (10)**

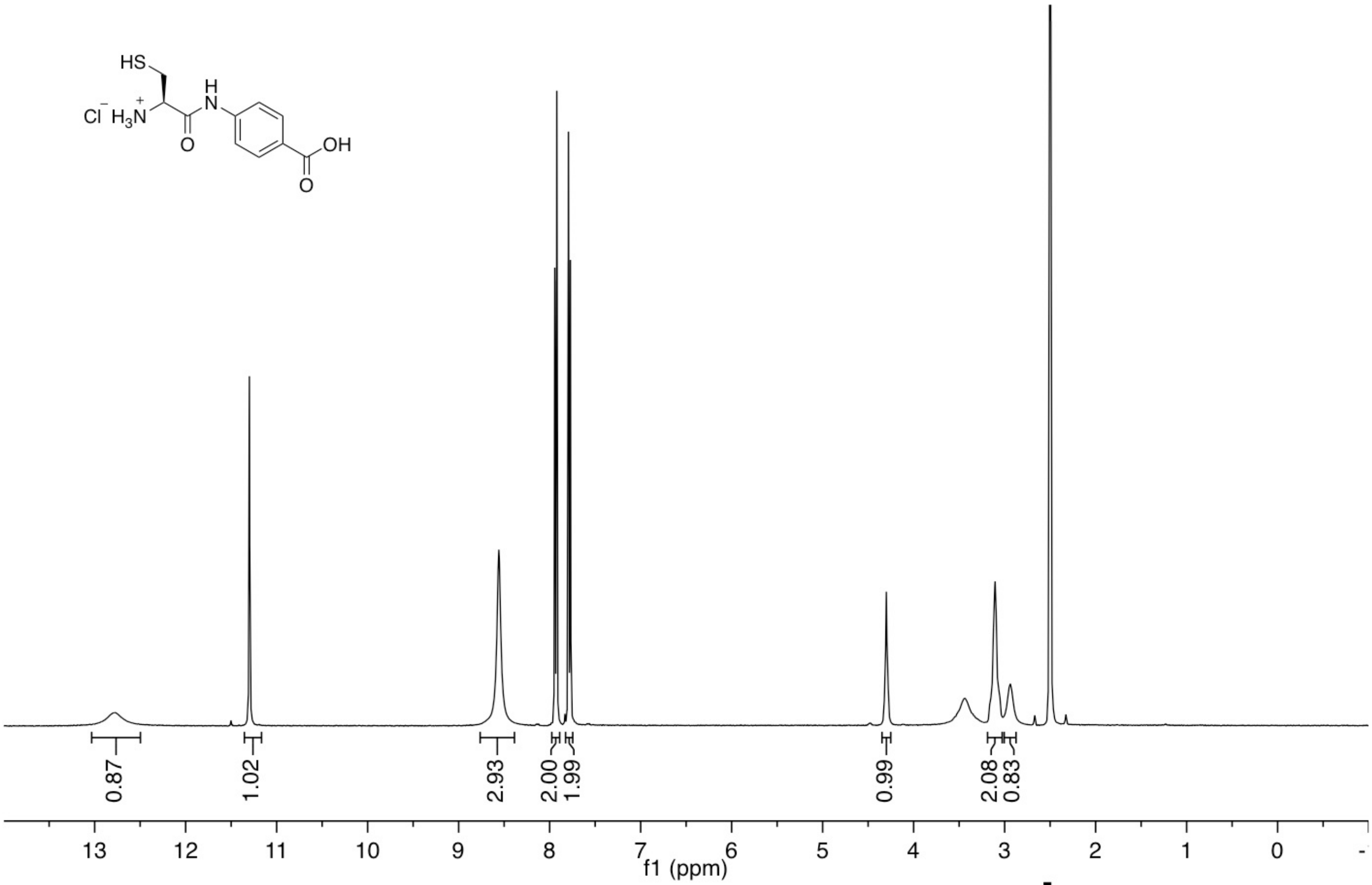


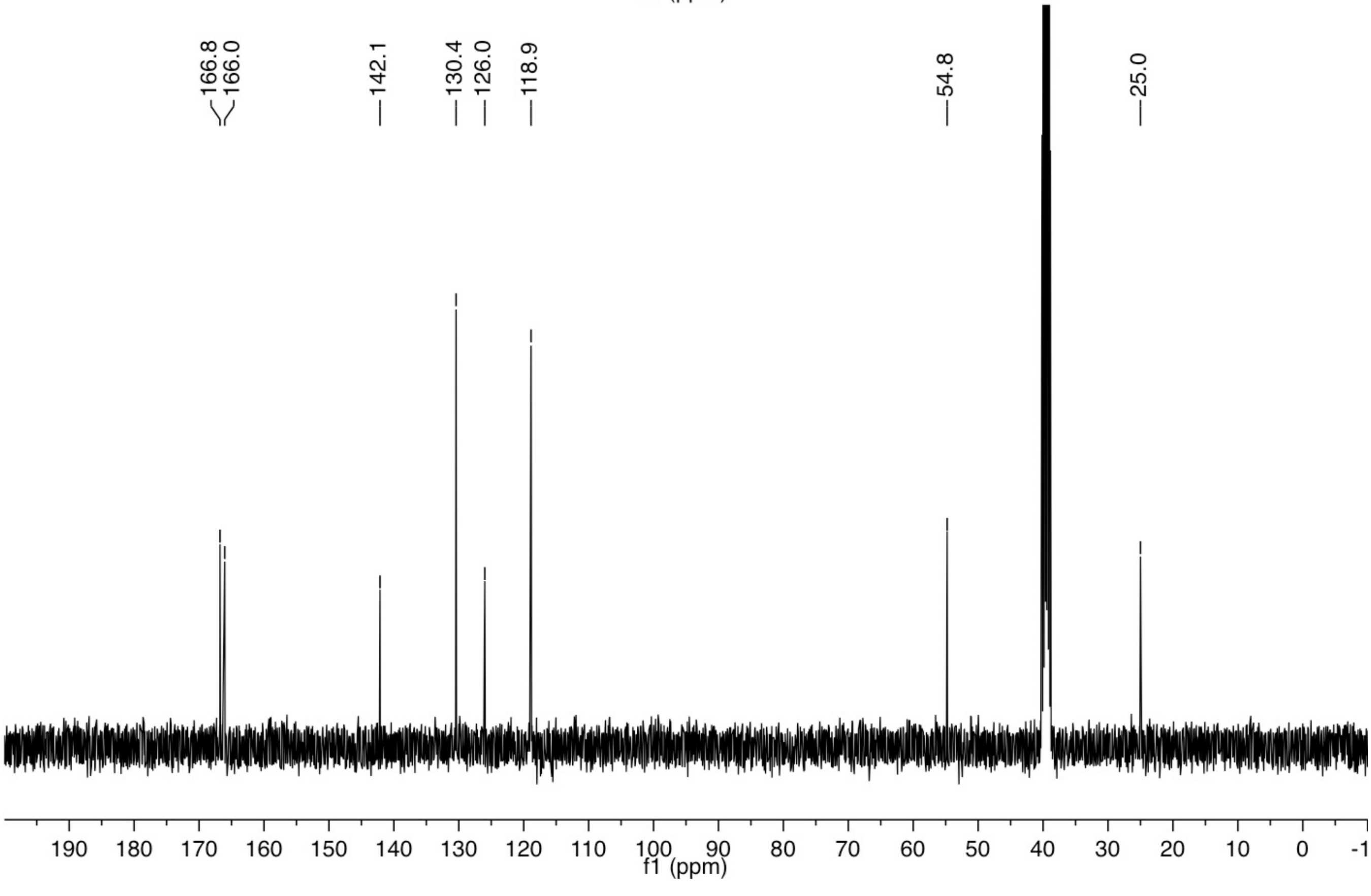

**(*R*)-2-Amino-3-mercapto-*N*-(3,4,5-trimethoxyphenyl)propanamide hydrochloride (11)**

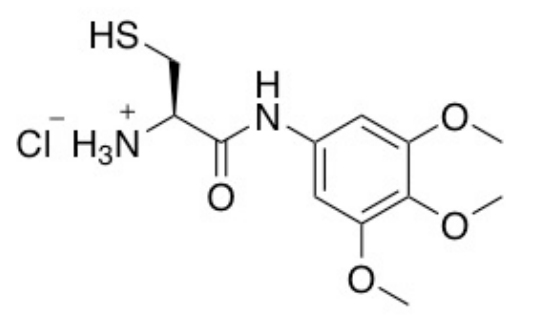


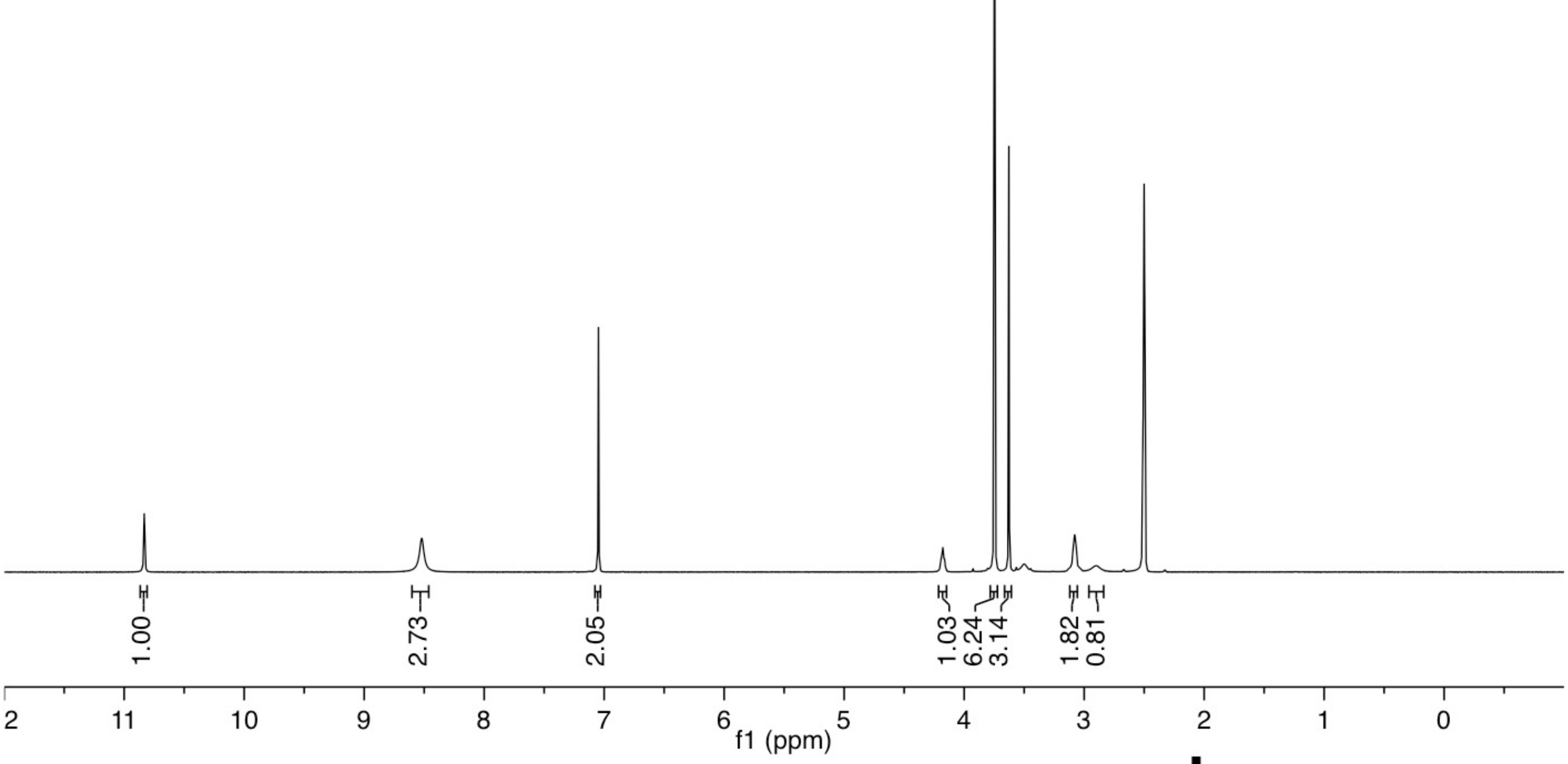


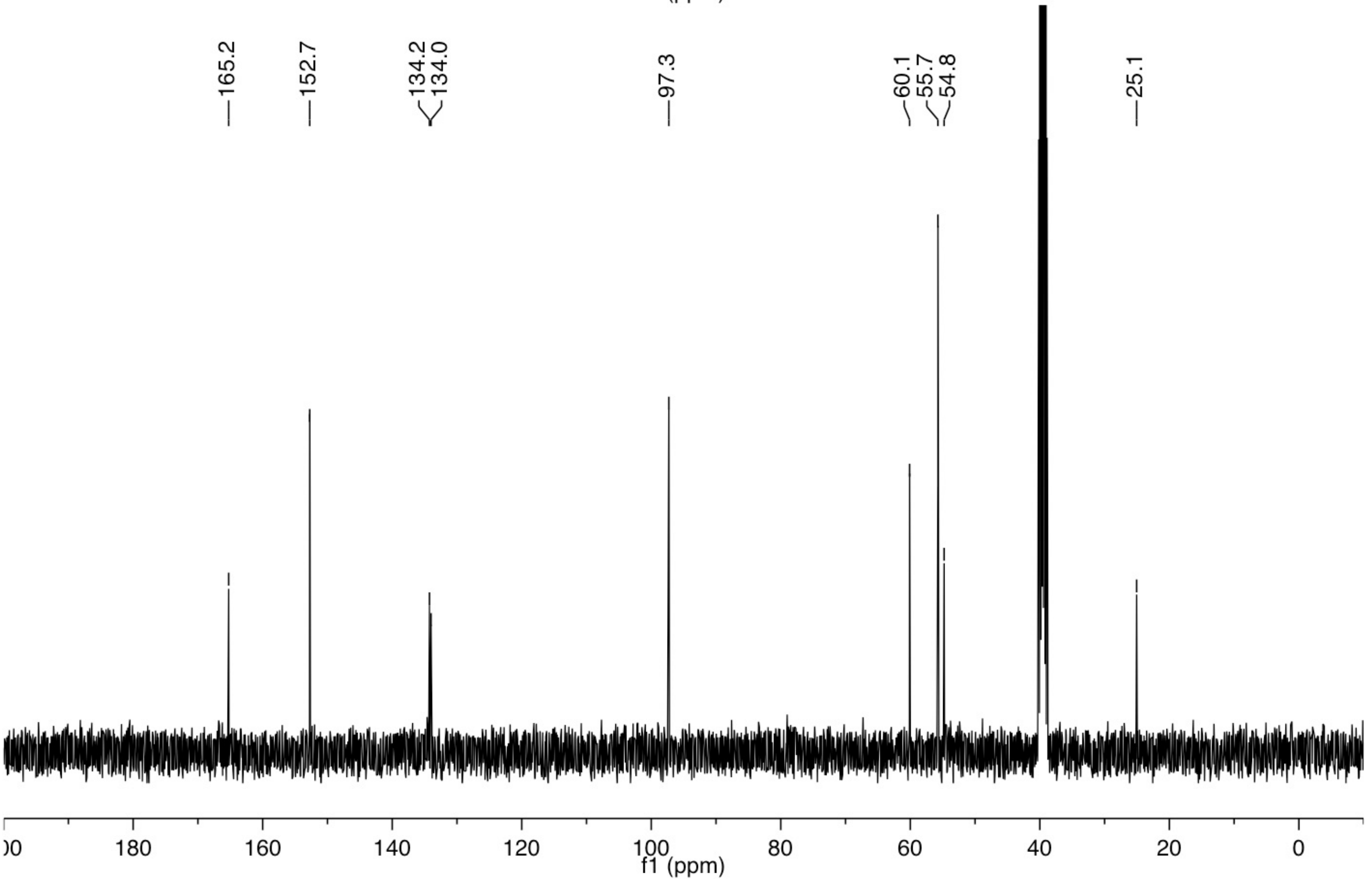

**(*R*)-2-Amino-3-mercaptopropanamide hydrochloride (12)**

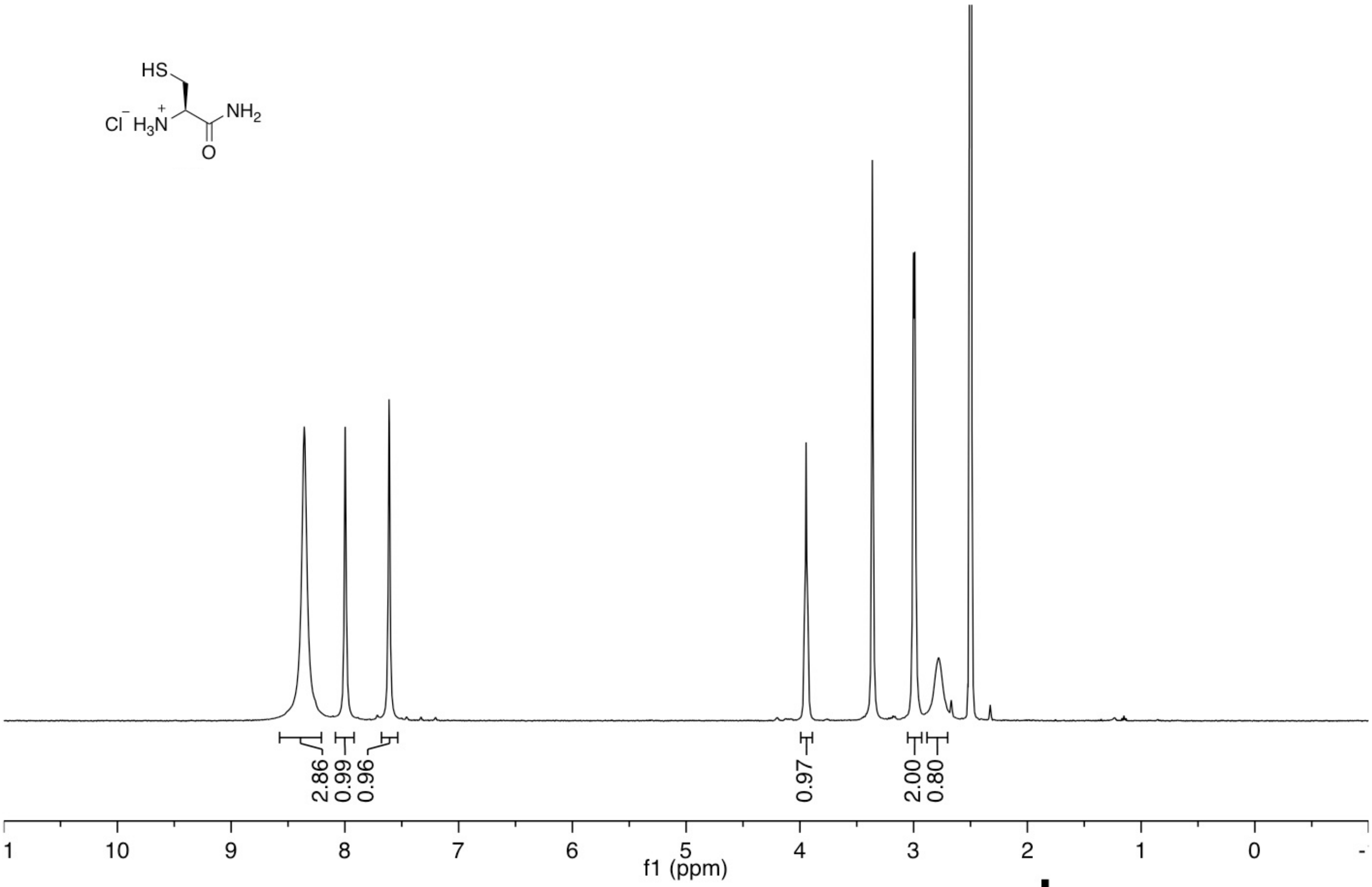


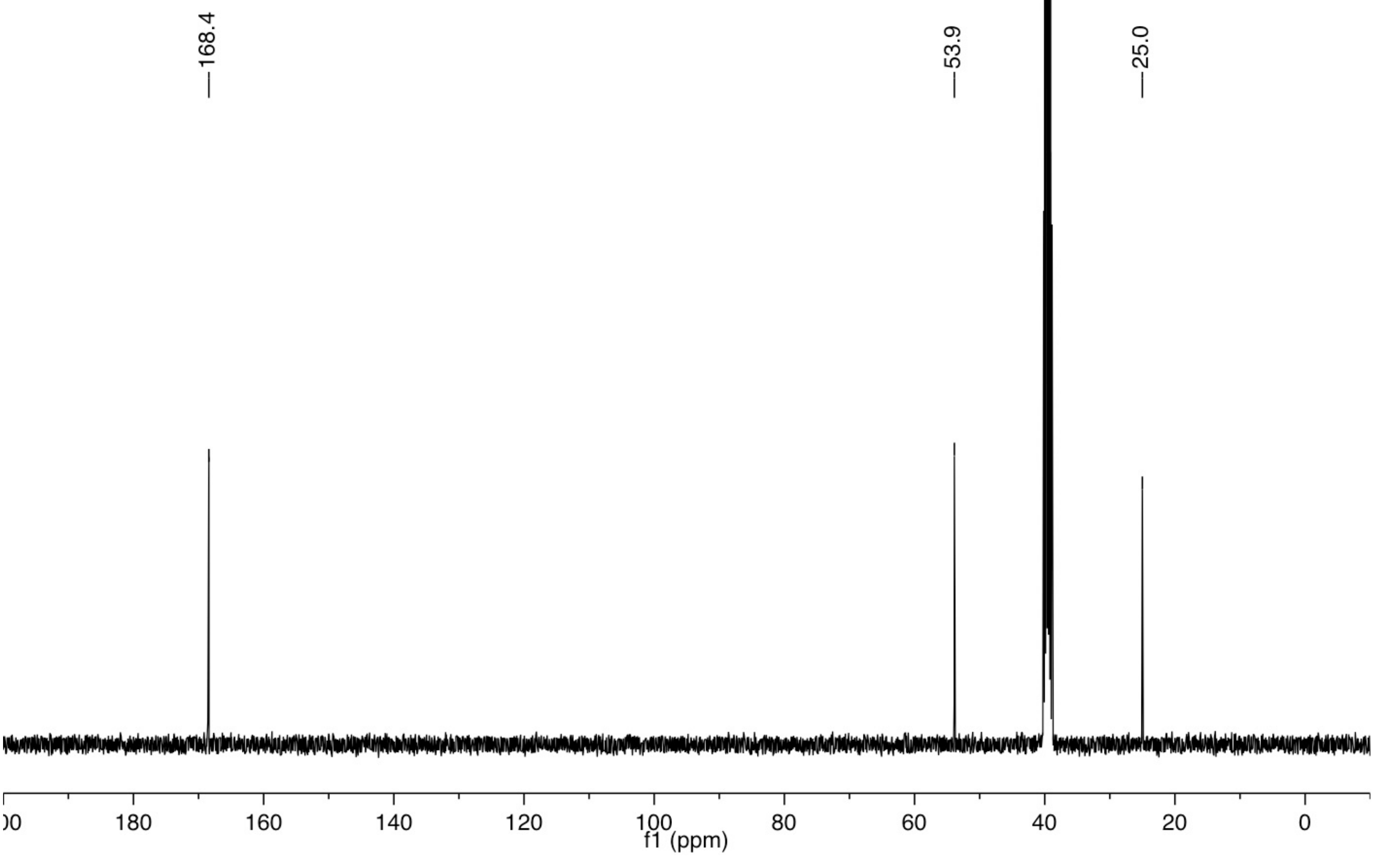

## Copies of HPLC chromatograms

**Compound 1** (>98% at 215 nm)

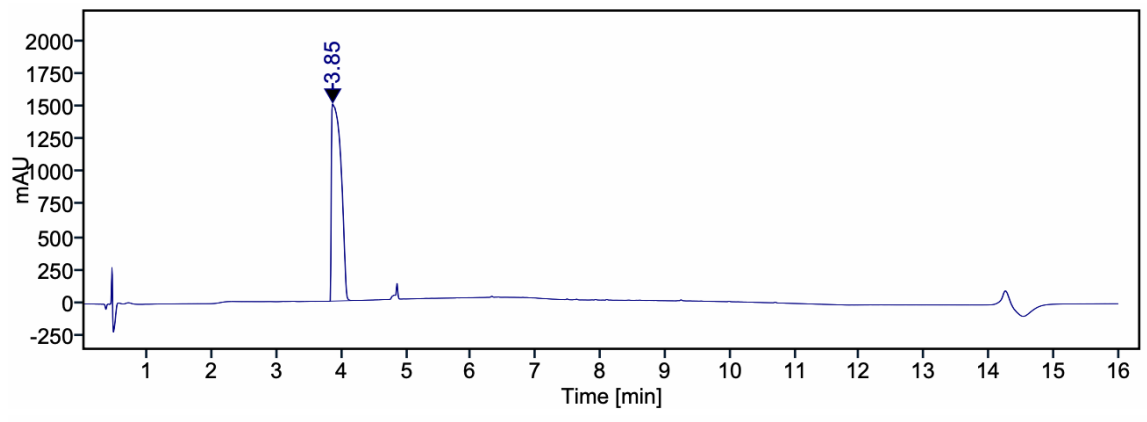


**Compound 5** (96% at 215 nm)

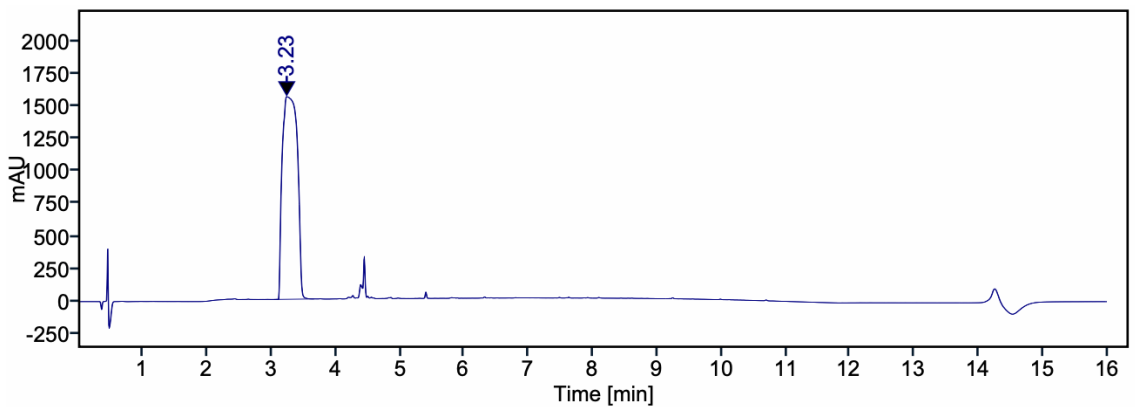


**Compound 10** (>98% at 215 nm)

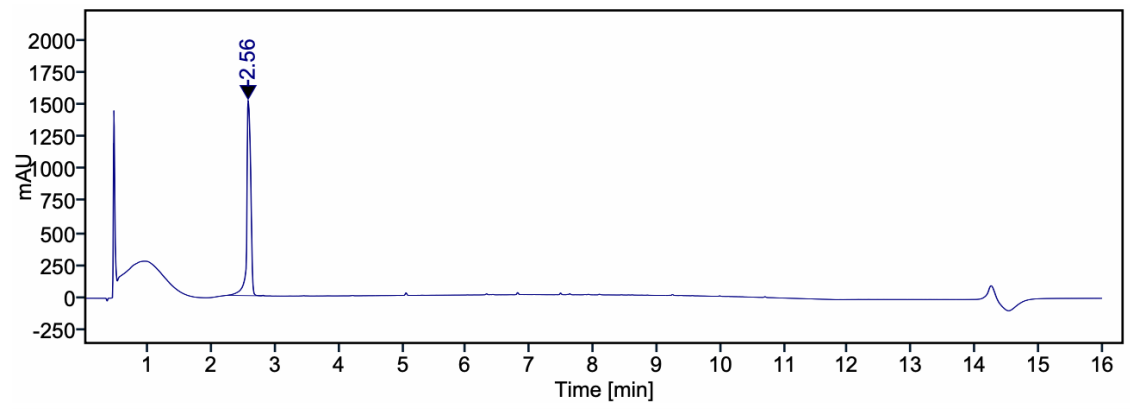


**Compound 11** (>98% at 215 nm)

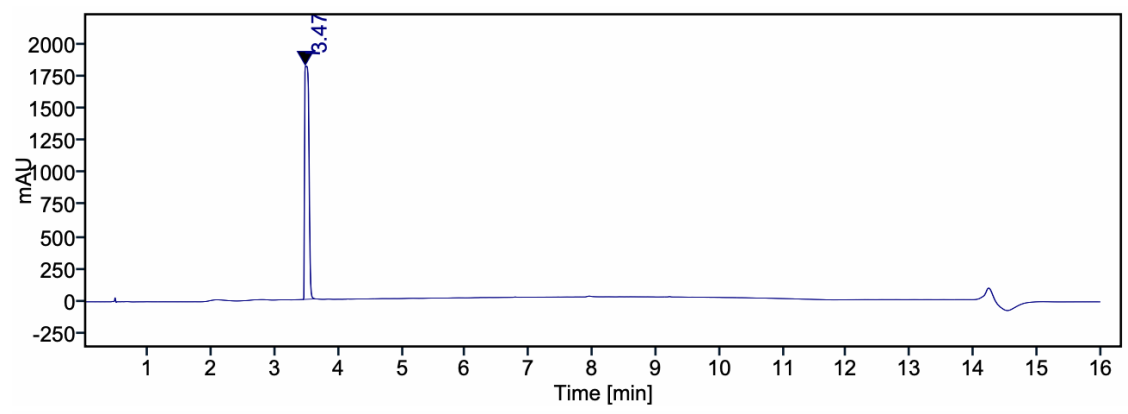

Supplement: Supplementary file 1 [file ANIE-65-e9489770-s001.pdf]
